# Effectiveness and cost-effectiveness analysis of 11 treatment paths, seven first-line and three second-line treatments for Chinese patients with advanced wild-type squamous non-small cell lung cancer: A sequential model

**DOI:** 10.3389/fpubh.2023.1051484

**Published:** 2023-02-24

**Authors:** Mingye Zhao, Taihang Shao, Zhuoyuan Chi, Wenxi Tang

**Affiliations:** ^1^Department of Pharmacoeconomics, School of International Pharmaceutical Business, China Pharmaceutical University, Nanjing, Jiangsu, China; ^2^Center for Pharmacoeconomics and Outcomes Research, China Pharmaceutical University, Nanjing, Jiangsu, China

**Keywords:** advanced squamous non-small cell lung cancer, cost-effectiveness, treatment sequence, sequential model, non-proportional hazard models

## Abstract

**Background:**

A total of 11 treatment sequences for advanced wild-type squamous non-small cell lung cancer are recommended by Chinese Society of Clinical Oncology Guidelines, consisting of seven first-line and three second-line treatments. Five of these treatments were newly approved in China between 2021 and 2022. We evaluated the effectiveness and cost-effectiveness of these strategies from the Chinese healthcare system perspective.

**Methods:**

Network meta-analysis with non-proportional hazards was used to calculate the relative efficacy between interventions. A sequential model was developed to estimate costs and quality-adjusted life years (QALY) for treatment sequences with first-line platinum- and paclitaxel-based chemotherapy (SC) with or without nedaplatin, tislelizumab, camrelizumab, sintilimab, sugemalimab or pembrolizumab, followed by second-line docetaxel, tislelizumab or nivolumab. SC and docetaxel were used as comparators for first-line and second-line treatments, respectively. QALY and incremental cost-effectiveness ratio (ICER) were used to evaluate effectiveness and cost-effectiveness, respectively. Cost-effective threshold was set as USD 19,091. Subgroup analysis was conducted to determine the best first-line and second-line therapy.

**Results:**

Pembrolizumab + SC, followed by docetaxel (PED) was the most effective treatment sequence. QALYs for patients received SC, nedaplatin + SC, tislelizumab + SC, sintilimab + SC, camrelizumab + SC, sugemalimab + SC, pembrolizumab + SC followed by docetaxel were 0.866, 0.906, 1.179, 1.266, 1.179, 1.266, 1.603, 1.721, 1.807; QALYs for SC, nedaplatin + SC followed by tislelizumab were 1.283, 1.301; QALYs for SC, nedaplatin + SC followed by nivolumab were 1.353, 1.389. Camrelizumab + SC, followed by docetaxel (CAD) was the most cost-effective. Compared to SC with or without nedaplatin, tislelizumab, or sintilimab followed by docetaxel, ICERs of CAD were USD 12,276, 13,210, 6,974, 9,421/QALY, respectively. Compared with nedaplatin or SC followed by tislelizumab, the ICERs of CAD were USD 4,183, 2,804/QALY; CAD was dominant compared with nedaplatin or SC followed by nivolumab; The ICER of sugemalimab + SC followed by docetaxel and PED were USD 522,023, 481,639/QALY compared with CAD. Pembrolizumab + SC and camrelizumab + SC were the most effective and cost-effective first-line options, respectively; tislelizumab was the most effective and cost-effective second-line therapy. Tislelizumab used in second-line was more effective than first-line, no significant differences between their cost-effectiveness. Sensitivity and scenario analysis confirmed robustness of the results.

**Conclusions:**

PED and CAD are the most effective and cost-effective treatment sequence, respectively; pembrolizumab + SC and camrelizumab + SC are the most effective and cost-effective first-line choice, respectively; tislelizumab is the most effective and cost-effective second-line choice.

## Highlights

- What is already known about the topic?

Non-small-cell lung cancer (NSCLC) poses a significant burden on patients and the healthcare system owing to decreased quality of life, substantial economic burden. A total of 11 treatment sequences for advanced wild-type squamous non-small cell lung cancer are recommended by Chinese Society of Clinical Oncology Guidelines, consisting of seven first-line and three second-line treatments, five of them were newly approved in Chinese between 2021 and 2022.

- What does the paper add to existing knowledge?

First-line camrelizumab plus carboplatin and paclitaxel, followed by second-line docetaxel is the optimal treatment sequence in cost-effectiveness, while pembrolizumab plus carboplatin and paclitaxel (SC), followed by second-line docetaxel is the optimal treatment sequence in effectiveness. Pembrolizumab plus SC (P + C) and camrelizumab plus SC (CA + C) are the most effective and cost-effective therapy among seven available first-line treatments, respectively (SC, nedaplatin, tislelizumab, camrelizumab, sintilimab, sugemalimab or pembrolizumab in combination with SC), tislelizumab is the best second-line choice compared to nivolumab and docetaxel both in effectiveness and cost-effectiveness.

- What insights does the paper provide for informing health care-related decision making?

We provided a novel mirco-simulation sequential model to determine the optimal therapeutic pathway as certain reference for future research. The current National Reimbursement Drug List (NRDL) negotiation attaches great importance to direct evidence between innovative treatments, traditional pharmacoeconomics research of innovative treatments vs. standard treatments may be no longer applicable. In the upcoming 2022 NRDL negotiation, our research will provide comprehensive evidence for drug access negotiation and price setting for the all first- or second-line treatments of sq-NSCLC.

## Introduction

The International Agency for Research on Cancer (https://www.iarc.who.int/) reported that, ~19.3 million new cancer cases and nearly 10 million cancer-related deaths occurred worldwide in 2020 (1). Lung cancer accounted for 11.4% of the new cancer cases, ranking second after breast cancer (11.7%), and 18% of new cancer-related deaths, ranking first among all cancers (1). Non-small cell lung cancer (NSCLC) accounted for 80–85% of all lung cancers (2, 3), and nearly one-third of patients with NSCLC are diagnosed with the squamous histological subtype (4). Treatment development for squamous NSCLC (sq-NSCLC) has been stagnated, owing to its unique histopathology and molecular characteristics (5).

Many chemotherapy drugs have been approved in China for treating sq-NSCLC, including cisplatin or carboplatin combined with gemcitabine, docetaxel, paclitaxel, or nedaplatin. Under chemotherapy treatment, patients with advanced sq-NSCLC have low survival rates, the median progression-free survival (PFS) of patients with stage IIIB–IV sq-NSCLC was ~4–6 months (6–16), and the median overall survival (OS) was 10–15 months (7–17), Programmed death-1 (PD-1) and programmed death-ligand 1 (PD-L1) immune checkpoint inhibitors are considered to be a breakthrough in the treatment of sq-NSCLC. PD-L1 is expressed in normal tissues but is overexpressed in various types of tumors. In NSCLC, PD-L1 expression levels were found to increase by 35–95% (18). Activation of immune cells increased the expression of the PD-1/PD-L1 immune checkpoint inhibitors and restored or even enhanced the ability of immune cells to kill tumor cells by blocking PD-1/PD-L1 expression (19). Many studies have shown that combining immunotherapy and chemotherapy can significantly improve PFS and OS in patients with stage IIIB–IV sq-NSCLC. Specifically, the median PFS was approximately 8–9 months, and the median OS was 15–18 months, both showed significant longer survival benefits than chemotherapy alone (10–15, 20). Many immune checkpoint inhibitors for treating advanced sq-NSCLC have been approved in China, including pembrolizumab, tislelizumab, camrelizumab, sintilimab, and sugemalimab, atezolizumab and nivolumab.

Although PD-1/PD-L1 inhibitors have improved outcomes in patients with metastatic diseases, they are also associated with significant higher cost. In current healthcare environments, policy makers, clinicians, and patients will all benefit from a sound framework for determining the benefits of different therapeutic choices in oncology based on both effectiveness and cost-effectiveness. The current National Reimbursement Drug List (NRDL) negotiation attaches great importance to direct evidence between innovative treatments, traditional pharmacoeconomics research of innovative treatments vs. standard treatments may be no longer applicable.

For the treatment of wild-type advanced sq-NSCLC, seven first-line treatments and three second-line treatments were first-level recommended by Clinical Oncology Guidelines 2022 (CSCO 2022) (21). Increasing in treatment options makes it more difficult to choose an effective and cost-effective clinical treatment path for clinicians and patients. More importantly, health policy makers are facing great challenges in drugs market access, market pricing, and rational allocation of health resources. Direct evidence between innovative treatments is more important for NRDL negotiation, therefore, there is an urgent need to systematically compare the effectiveness and cost-effectiveness of these treatments or sequential pathways, so as to promote clinical rational drug use, scientific formulation of health policy and rational allocation of medical resources. Therefore, evidence of systematic evaluation of same-type therapies is urgently needed. Therefore, we mainly aimed to evaluate the effectiveness and cost-effectiveness of currently available first-line therapies, second-line therapies and treatment sequences recommended by CSCO 2022 for patients with wild-type advanced sq-NSCLC (21).

## Materials and methods

### Target population and treatment strategies

**Figure 1:**
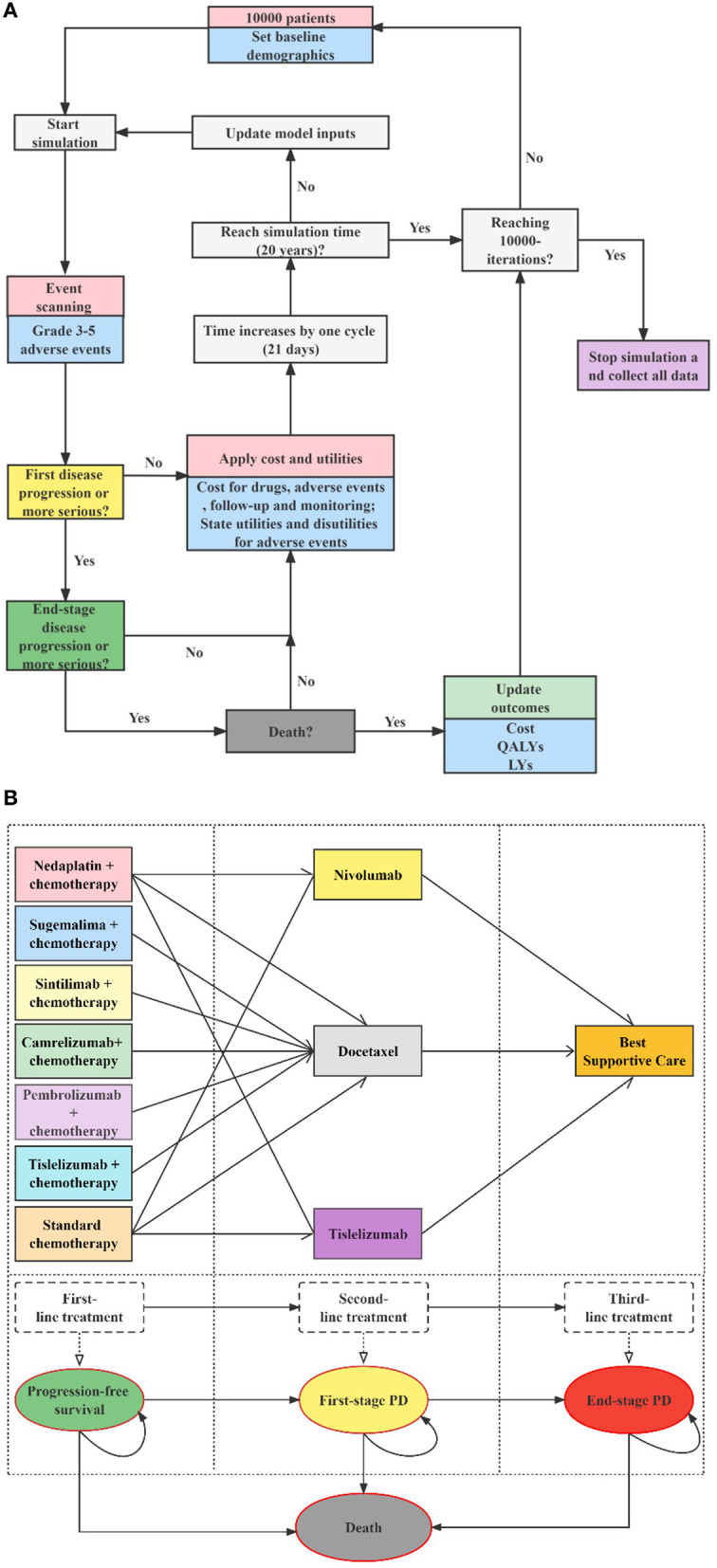
Decision analytic model and treatment strategies. **(A)** Microsimulation model structure (above), **(B)** Multi-state survival model for treatment strategies (below), PD, progressive disease; LYs, life-years; QALYs, quality-adjusted life years.

The target population was Chinese adults (aged ≥ 18 years) who had pathologically confirmed stage IIIB–IV wild-type sq-NSCLC with unlimited PD-L1 expression. The population received no previous systemic therapy. We modeled a hypothetical cohort with the same baseline characteristics as the patients enrolled in the original clinical trials. For dosage calculation, the body surface area and creatinine clearance rate were assumed as 1.72 m^2^ and 70 ml/min (22). According to the CSCO 2022 (21), the first-level recommended first-line regimens for performance status (PS) 0–1 patients with advanced sq-NSCLC and unlimited PD-L1 expression include cisplatin or carboplatin combined with gemcitabine, docetaxel, or paclitaxel (standard chemotherapy), nedaplatin combined with docetaxel (N + C), paclitaxel and platinum combined with pembrolizumab (P + C), paclitaxel and platinum combined with tislelizumab (T + C), paclitaxel and platinum combined with camrelizumab (CA + C), platinum combined with gemcitabine and sintilimab (SI + C), paclitaxel and platinum combined with sugemalimab (SU + C). Among these seven first-line therapies, T + C, CA + C, SI + C, and SU + C were newly approved for sq-NSCLC since 2021 in China. Nivolumab, tislelizumab and docetaxel are first-level recommended second-line treatments options for these patients, and tislelizumab was newly approved in 2022 for second-line treatment of sq-NSCLC. Because of the possible resistance among PD-1/PD-L1 drugs, few clinical applications and evidence, we did not consider cases where immune checkpoint inhibitors were used in the first- and second-line treatments simultaneously. Therefore, we assessed 11 treatment strategies (see Figure 1): 1. first-line N + C followed by second-line docetaxel (ND); 2. first-line N + C followed by second-line tislelizumab (NT); 3. first-line N + C followed by second-line nivolumab (NN) (16); 4. first-line standard chemotherapy followed by second-line docetaxel (CD); 5. first-line standard chemotherapy followed by second-line tislelizumab (CT); 6. first-line standard chemotherapy followed by second-line nivolumab (CN) (10–13, 16, 20); 7. first-line P + C followed by second-line docetaxel (PED) (13); 8. first-line SI + C followed by second-line docetaxel (SID) (12); 9. first-line CA + C followed by second-line docetaxel (CAD) (11); 10. first-line T + C followed by second-line docetaxel (TID) (20); 11. first-line SU + C followed by second-line docetaxel (SUD) (10). According to randomized clinical trials (RCTs) (23, 24), clinical diagnosis, and treatment experience (25, 26), the PS of patients with advanced sq-NSCLC tends to be poor after two-line active treatments. Therefore, the best supportive treatment (BSC) accounts for the largest proportion of third-line treatment, surpassing sum of other active treatments' proportions. Thus, patients with disease progression after the first- and second-line treatments were assumed to receive the BSC in this model. Standard chemotherapy and docetaxel were used as comparators for first-line and second-line treatments, respectively. We explored the impact of uncertainty about the third-line treatment on the results by scenario analysis. Specific medication, dosages, treatment durations are provided in the Supplementary material 1.

### Decision analytic model

We developed a sequential micro-simulation model in an academic medical setting with 21-day cycle length to compare different treatment strategies in the context of the Chinese healthcare system. The sequential model is a modification of the traditional partitioned survival model. In the traditional three-state partitioned survival model, post-progression treatment pathways are indistinguishable, and cycle costs for all PD stages can only be unique. However, for sq-NSCLC, the treatment of patients after progression follows certain treatment pathways, i.e., second-line treatment, then third-line... until finally they would receive best supportive care and end-of-life treatment. It is in this context that the sequential model is created, enabling accurate simulation of multiple lines of treatment pathways for patients, thereby improving the accuracy of cost and health. A cohort of 10,000 simulated patients with advanced sq-NSCLC experienced four states: PFS, first-stage progressed disease (PD), end-stage PD, and death. All the simulated patients began progression-free before receiving first-line therapies, and those with PD were followed up through second-line treatment, third-line treatment, and death. Details of the model structure and treatment strategies are shown in Figure 1, modeling process and validation are provided in Supplementary material 2. Microsoft Excel 2019 was used for model building. The reporting of the economic evaluation followed the ISPOR guideline Consolidated Health Economic Evaluation Reporting Standards (CHEERS) checklist (Supplementary material 4).

### Sources of treatment efficacy

Relative efficacy of the different treatments compared to the reference treatments were assessed by network meta-analysis (NMA). Briefly, we systematically searched PubMed, Embase, ClinicalTrials.Gov, European Society for Medical Oncology, American Society of Clinical Oncology, and World Conference on Lung Cancer databases as of May 2022 (27–31). Bayesian parametric survival NMA was used to synthesize survival data from eligible trials. Details of the eligibility criteria, search strategies are provided in Supplementary material 2. We conducted three NMAs in our study. For the NMA of first-line PFS, we estimated the time-varying hazard ratios (HRs) between the combination therapies N + C, P + C, T + C, CA + C, SI + C or SU + C and standard chemotherapy. Then, the expected survival curves for the combination therapies were derived by applying the HRs to the Kaplan-Meier survival curves for standard chemotherapy (reference treatment). The reference PFS curve for the first-line was derived from the CameL-sq, in which the final rate of the PFS was 5% (11). For this analysis, in the platinum- and paclitaxel-based chemotherapy regimens, cisplatin and carboplatin, and paclitaxell, gemcitabine, and docetaxel were not differentiated because their prices were similarly low and their survival outcomes were almost the same, and these drugs were used in similar capacities in common clinical practice (6, 32, 33). Similar to the first-line NMA, for the second-line NMAs of PFS and OS, we estimated the HRs between nivolumab, tislelizumab and docetaxel. The referred PFS and OS curves were extracted from the docetaxel in Checkmate-078 China (final rates of PFS and OS were < 3 and 5% for docetaxel) (23, 24). We also considered natural mortality after the plateau at the end of the survival curves, which were extracted from China's 6th National Census (34). The original PFS and OS curves used in this study are presented in Supplementary material 2.

### Model transitions and survival estimates

We used GetData Graph Digitizer (v2.26, http://getdata.sourceforge.net/download.html) to extract survival data from published PFS and OS Kaplan-Meier curves. To reconstruct individual patient data, we used the Guyot's method, which is the most accurate data reproduction method currently known for cases where individual patient data are not available (35, 36). Log cumulative hazards and schoenfeld residual test plots (Supplementary material 2) showed proportional hazard (PH) or piecewise models were not suitable in this analysis. In accordance with the shapes of the survival curves, the non-PH NMA models considered in this study were first- and second-order fractional polynomial (FP) models (37). We fitted first- and second-order FP models with power parameters −2, −1, −0.5, 0, 0.5, 1, 2, and 3, with three parallel Markov chains consisting of 10,000 samples after a 10,000 samples burn-in. To reconstruct and extrapolate the PFS curve of the standard chemotherapy, and the OS and PFS curves of the second-line docetaxel, we considered parametric functions including Exponential, Weibull, Gompertz, Gamma, Log-logistic, Log-normal, Generalized Gamma, GenF, FP, Restricted Cubic Spline, and Royston and Parmar (RP) models. Goodness-of-fit was evaluated by visual inspection of survival curves, Akaike information criterion (AIC) and deviance information criterion (DIC). Lower AIC and DIC combined with reasonable visual effects indicated a better performance of the selected model (38). Survival modeling was conducted in R (v4.1.2) and Winbugs (v1.4.3) (39, 40). R codes for relative methods can be found on Github (https://github.com/TaihangShao/NMA_methodology).

### Model validation

The face validity (model structure and assumption, data sources, and results) of the model was evaluated by clinical experts. Authors MZ and TS did the coding, and the results produced by the model were compared with previously reported results for cross-validation.

### Costs

The costs of implementing each treatment were derived the perspective of Chinese healthcare system. All cost data were inflated to 2022, shown as 2022 US dollars (1 USD = 6.36 Chinese Yuan). We considered only direct medical costs, including drug costs, follow-up costs, monitoring costs, death costs, and costs for treatment of adverse reactions (AEs). Drug prices were obtained from the latest local public bid-winning price or public databases (41–43). The prices of camrelizumab used in first-line or tislelizumab used in second-line were assumed to be the same as other indications of them which have entered the NRDL, considering the newly approved indication of sq-NSCLC would likely to be included in the list and the price is the same for all indications of the same drug in the NRDL. Prices for paclitaxel and gemcitabine were from the fifth batch of bids for centralized drug procurement of drugs in China in 2021 (41–43). Because carboplatin, cisplatin, paclitaxel, docetaxel, and nedaplatin have multiple dosage forms in the Chinese market, we chose the commonly used dosage combination under the principle of minimizing cost. Follow-up costs and monitoring costs were derived from the healthcare documents (44), which included CT examination, blood test, urinalysis, and blood biochemical examination, as wells as diagnosis fee, injection fee, nursing fee, and bed fee. Costs of BSC and end-of-life were extracted from published literature. We considered only severe AEs (≥grade 3) with rates >5%. AE related treatment costs and durations of AE were extracted from published articles. All AEs were assumed to occur during the first cycle (45). Details are listed in Table 1.

**Table 1:** Parameters used in the model.

**Item**	**Mean (range)**	**Distribution**	**Sources**
**Clinical-related parameters**			
**First-order fractional polynomial model for first-line PFS curve:*****p*** = −**2**			
PFS_HR_Scale (N + C vs. Standard chemotherapy)	−0.016 (-0.499 ~ 0.467)	Lognormal	NMA
PFS_HR_Scale (SI + C vs. Standard chemotherapy)	−0.735 (-1.029 ~ 0.442)	Lognormal	NMA
PFS_HR_Scale (P + C vs. Standard chemotherapy)	−1.255 (-1.678 ~−0.832)	Lognormal	NMA
PFS_HR_Scale (T + C vs. Standard chemotherapy)	−0.589 (-0.99 ~−0.197)	Lognormal	NMA
PFS_HR_Scale (CA + C vs. Standard chemotherapy)	−1.095 (-1.368 ~−0.828)	Lognormal	NMA
PFS_HR_Scale (SU + C vs. Standard chemotherapy)	−1.191 (-1.58 ~−0.806)	Lognormal	NMA
PFS_HR_Shape (N + C vs. Standard chemotherapy)	−4.314 (-11.076 ~ 2.094)	Lognormal	NMA
PFS_HR_Shape (SI + C vs. Standard chemotherapy)	0.849 (-1.671 ~ 3.263)	Lognormal	NMA
PFS_HR_Shape (P + C vs. Standard chemotherapy)	0.934 (-2.192 ~ 3.717)	Lognormal	NMA
PFS_HR_Shape (T + C vs. Standard chemotherapy)	−0.404 (-3.068 ~ 1.877)	Lognormal	NMA
PFS_HR_Shape (CA + C vs. Standard chemotherapy)	1.022 (-0.826 ~ 2.792)	Lognormal	NMA
PFS_HR_Shape (SU + C vs. Standard chemotherapy)	1.548 (-0.655 ~ 4.071)	Lognormal	NMA
**Second-order fractional polynomial model for first-line OS curve: p1** = −**0.5, p2** = **0**			
OS_HR_Scale (Nivolumab vs. Docetaxel)	2.231 (-3.239 ~ 7.493)	Lognormal	NMA
OS_HR_Scale (Tislelizumab vs. Docetaxel)	0.151 (-6.431 ~ 6.387)	Lognormal	NMA
OS_HR_Shape1 (Nivolumab vs. Docetaxel)	−3.328 (-9.684 ~ 3.238)	Lognormal	NMA
OS_HR_Shape1 (Tislelizumab vs. Docetaxel)	−0.786 (-8.365 ~ 7.201)	Lognormal	NMA
OS_HR_Shape2 (Nivolumab vs. Docetaxel)	−0.677 (-2.045 ~ 0.755)	Lognormal	NMA
OS_HR_Shape2 (Tislelizumab vs. Docetaxel)	0.187 (-1.822 ~ 1.54)	Lognormal	NMA
**Second-order fractional polynomial model for first-line PFS curve:** ***p*** = −**2**			
PFS_HR_Scale (Nivolumab vs. Docetaxel)	−0.891 (-1.263 ~−0.511)	Lognormal	NMA
PFS_HR_Scale (Tislelizumab vs. Docetaxel)	−1.059 (-1.347 ~−0.763)	Lognormal	NMA
PFS_HR_Shape (Nivolumab vs. Docetaxel)	0.675 (-0.253 ~−1.641)	Lognormal	NMA
PFS_HR_Shape (Tislelizumab vs. Docetaxel)	0.449 (-0.483 ~ 1.39)	Lognormal	NMA
**Parametric model fit to the referred PFS and OS curves**			
Log-logistic model for the first-line PFS curve (scale)	0.38	Constant	Parametric model
Log-logistic model for the first-line PFS curve (shape)	2.506	Constant	Parametric model
Exponential model for the second-line OS curve (scale)	1.043	Constant	Parametric model
Restricted cubic spline model for the second-line PFS curve (Gamma 0)	0.463	Constant	Parametric model
Restricted cubic spline model for the second-line PFS curve (Gamma 1)	0.305	Constant	Parametric model
Restricted cubic spline model for the second-line PFS curve (Gamma 2)	1.793	Constant	Parametric model
Restricted cubic spline model for the second-line PFS curve (Gamma 3)	0.114	Constant	Parametric model
**Risk of grade 3–5 adverse events**			
Neutropenia (P + C)	0.615 (0.492 ~ 0.738)	Beta	(13)
Neutropenia (SI + C)	0.486 (0.389 ~ 0.583)	Beta	(12)
Neutropenia (T + C)	0.517 (0.413 ~ 0.620)	Beta	(20)
Neutropenia (CA + C)	0.554 (0.444 ~ 0.665)	Beta	(11)
Neutropenia (N + C)	0.270 (0.216 ~ 0.323)	Beta	(16)
Neutropenia (Standard chemotherapy)^†^	0.488 (0.391 ~ 0.586)	Beta	(10–13, 16, 20)
Neutropenia (Docetaxel)	0.590 (0.472 ~ 0.708)	Beta	(46)
Neutropenia (SU + C)	0.325 (0.26 ~ 0.39)	Beta	(10)
Decreased platelet count (P + C)	0.077 (0.062 ~ 0.092)	Beta	(13)
Decreased platelet count (SI + C)	0.453 (0.362 ~ 0.543)	Beta	(12)
Decreased platelet count (T + C)	0.058 (0.047 ~ 0.07)	Beta	(20)
Decreased platelet count (CA + C)	0.067 (0.054 ~ 0.081)	Beta	(11)
Decreased platelet count (Standard chemotherapy)^†^	0.171 (0.136 ~ 0.205)	Beta	(10–13, 16, 20)
Decreased platelet count (SU + C)	0.103 (0.083 ~ 0.124)	Beta	(10)
Anemia (SU + C)	0.134 (0.108 ~ 0.161)	Beta	(10)
Anemia (SI + C)	0.335 (0.268 ~ 0.402)	Beta	(12)
Anemia (T + C)	0.075 (0.06 ~ 0.09)	Beta	(20)
Anemia (CA + C)	0.104 (0.083 ~ 0.124)	Beta	(11)
Anemia (Standard chemotherapy)^†^	0.143 (0.115 ~ 0.172)	Beta	(10–13, 16, 20)
Leukopenia (P + C)	0.354 (0.283 ~ 0.425)	Beta	(13)
Leukopenia (SI + C)	0.363 (0.291 ~ 0.436)	Beta	(12)
Leukopenia (Standard chemotherapy)^†^	0.284 (0.227 ~ 0.341)	Beta	(10–13, 16, 20)
Leukopenia (T + C)	0.225 (0.18 ~ 0.27)	Beta	(20)
Leukopenia (CA + C)	0.301 (0.24 ~ 0.361)	Beta	(11)
Leukopenia (N + C)	0.177 (0.142 ~ 0.233)	Beta	(16)
Leukopenia (SU + C)	0.141 (0.113 ~ 0.169)	Beta	(10)
Leukopenia (Docetaxel)^‡^	0.342 (0.274 ~ 0.41)	Beta	(46)
Pneumonia (SI + C)	0.14(0.112 ~168)	Beta	(12)
Pneumonia (Standard chemotherapy)^†^	0.094 (0.076 ~ 0.113)	Beta	(10–13, 16, 20)
Pneumonia (Tislelizumab)	0.089 (0.071 ~ 0.107)	Beta	(46)
Hyponatremia (SI + C)	0.061 (0.049 ~ 0.074)	Beta	(12)
Hyponatremia (Standard chemotherapy)^†^	0.05 (0.04 ~ 0.06)	Beta	(10–13, 16, 20)
Asthenia (Docetaxel)^‡^	0.051 (0.041 ~ 0.062)	Beta	(46)
**Time duration of grade 3–5 adverse events/days**			
Neutropenia	6.4	Constant	(47)
Decreased platelet count	8.5	Constant	(47)
Anemia	51.2	Constant	(47)
Leukopenia	4.5	Constant	(47)
Pneumonia	10.0	Constant	(48)
Hyponatremia	8.0	Constant	(49)
Asthenia	7.0	Constant	Assumed
**Cost-related parameters**			
**Cost of drugs/$**			
Pembrolizumab/100 mg	2816.87 (1408.43 ~ 2816.87)	Gamma	(41, 43)
Camrelizumab/200 mg	460.31 (368.25 ~ 460.31)	Gamma	(41, 43)
Sintilimab/100 mg	169.79 (135.83 ~ 169.79)	Gamma	(41, 43)
Tislelizumab/100 mg	227.95 (182.36 ~ 227.95)	Gamma	(41, 43)
Sugemalimab/600 mg	1,945 (973 ~ 1,945)	Gamma	(41, 43)
Nivolumab/100 mg	1454.18 (727.09 ~ 1454.18)	Gamma	(41, 43)
Nedaplatin/50 mg	47.05 (42.74 ~ 51.36)	Gamma	(41, 43)
Carboplatin/100 mg	8.13 (8.13 ~ 8.65)	Gamma	(41, 43)
Cisplatin /10 mg	1.47 (1.38 ~ 1.47)	Gamma	(41, 43)
Cisplatin /30 mg	3.01 (3.01 ~ 4.40)	Gamma	(41, 43)
Docetaxel/20 mg	3.55 (3.54 ~ 8.51)	Gamma	(41, 43)
Paclitaxel/100 mg	27.98 (27.98 ~ 27.98)	Gamma	(41, 42)
Paclitaxel/30 mg	10.57 (10.57 ~ 10.57)	Gamma	(41, 42)
Albumin paclitaxel/100 mg	109.73 (109.72 ~ 109.73)	Gamma	(41, 43)
Gemcitabine/200 mg	9.43 (9.42 ~ 9.43)	Gamma	(41, 42)
Best supportive care/cycle	337.95 (270.36 ~ 405.54)	Gamma	(50)
Cost of end-of-life	2325.75 (1860.6 ~ 2790.9)	Gamma	(50)
**Market shares**			
Paclitaxel	0.61 (0.49 ~ 0.73)	Beta	(47)
Carboplatin	0.74 (0.59 ~ 0.89)	Beta	
**Cost of follow-up and monitoring/$**			
Cost of CT examination/1 time	58.17 (45.99 ~ 68.98)	Gamma	(44)
Cost of blood biochemical examination/1 time	47.05 (37.2 ~ 55.8)	Gamma	(44)
Cost of blood test/1 time	3.14 (2.49 ~ 3.73)	Gamma	(44)
Cost of urinalysis/1 time	0.63 (0.5 ~ 0.75)	Gamma	(44)
Cost of diagnosis/	3.14 (1.55 ~ 4.66)	Gamma	(44)
Cost of intravenous injection/1 time	1.73 (1.55 ~ 2.14)	Gamma	(44)
Cost of nursing/1 time	3.77 (2.98 ~ 4.47)	Gamma	(44)
Cost of bed/1 time	6.6 (5.22 ~ 7.83)	Gamma	(44)
**Cost of grade 3–5 adverse events/$**			
Neutropenia	116.37 (51.11 ~ 357.8)	Gamma	(47)
Decreased platelet count	1523.82 (1240.17 ~ 1771.67)	Gamma	(47)
Anemia	140.4 (106.73 ~ 160.1)	Gamma	(47)
Leukopenia	116.37 (51.11 ~ 357.8)	Gamma	(47)
Pneumonia	1,640 (1,312 ~ 1,968)	Gamma	(26)
Hyponatremia	3,223 (2578.4 ~ 3867.6)	Gamma	(49)
Asthenia	107 (80 ~ 134)	Gamma	(51)
**Utility-related parameters**			
**Utilities for each state (base-case analysis)**			
Progression-free survival (immunotherapy)	0.75 (0.71 ~ 0.85)	Beta	(47)
Progression-free survival (chemotherapy)	0.70 (0.66 ~ 0.80)	Beta	(47, 52, 53)
Progression disease	0.59 (0.47 ~ 0.71)	Beta	(47)
**Utilities for each state (scenario 1)**			
Progression-free survival	0.804 (0.764 ~ 0.844)	Beta	(54)
Progression disease	0.321 (0.305 ~ 0.337)	Beta	(54)
**Utilities for each state (scenario 2)**			
Progression-free survival (immunotherapy)	0.877 (0.850 ~ 0.904)	Beta	(52)
Progression-free survival (chemotherapy)	0.823 (0.775 ~ 0.871)	Beta	(52)
Progression disease (second-line treatment)	0.768 (0.721 ~ 0.815)	Beta	(52)
Progression disease (third-line treatment)	0.703 (0.632 ~ 0.774)	Beta	(52)
**Disutilities for grade 3–5 adverse events**			
Neutropenia	0.2 (0.16 ~ 0.24)	Beta	(47)
Decreased platelet count	0.11 (0.09 ~ 0.13)	Beta	(47)
Anemia	0.07 (0.06 ~ 0.09)	Beta	(47)
Leukopenia	0.2 (0.16 ~ 0.24)	Beta	(47)
Pneumonia	0.05 (0.04 ~ 0.06)	Beta	(26)
Hyponatremia	0.08 (0.06-0.1)	Beta	(49)
Asthenia	0.07 (0.06 ~ 0.08)	Beta	(54)
**Other**			
Discount	0.05 (0.00–0.08)	Beta	(55)

N + C, Nedaplatin in combination with standard chemotherapy; SI + C, Sintilimab in combination with standard chemotherapy; P + C, Pembrolizumab in combination with standard chemotherapy; T + C, Tislelizumab in combination with standard chemotherapy; CA + C, Camrelizumab in combination with standard chemotherapy; SU + C, Sugemalimab in combination with standard chemotherapy.^†^Average of five first-line randomized controlled trials;^‡^ No sq-NSCLC subgroup safety data from Checkmate-078 China (54).

### Utilities

Health state utilities were sourced from published literature. For the base-case analysis, utilities were derived from the patient-level European Organization for Research and Treatment Quality of Life Questionnaire-Core 30 (EORTC QLQ-C30) scores in Orient-11 (56) by mapping to the EuroQol-5-dimension-5 level (EQ-5D-5L) (47). According to Shen et al. (52) and Nafees et al. (54), the utilities of patients receiving chemotherapy for PFS were 0.05 smaller than the utilities of those receiving immunotherapy. The EQ-5D utilities were 0.75 (immunotherapy) and 0.70 (chemotherapy) for PFS, and 0.59 for first- or end-stage PD. Considering the uncertainty of utilities which may have significant influences on the results, we used the utilities of Shen et al. (52) and Nafees et al. (54) to conduct two additional scenario analyses. The utility of the death state was specified as 0. Disutilities of AEs were extracted from other studies of Chinese patients. More details are shown in Table 1.

### Cost-effectiveness analysis

We evaluated the cost-effectiveness of these strategies from the Chinese healthcare system perspective, the simulated cohort was modeled for 20 years, at which point the mortality rate was 99%, which is the lifetime horizon recommended (55). The expected costs and quality-adjusted life years (QALYs) for each treatment were derived by assigning the corresponding costs and utilities to the time patients in each health state. Cost-effectiveness was measured by the incremental cost-effectiveness ratio (ICER) and incremental net monetary benefit (INMB). Recommended according to China Guidelines for Pharmacoeconomic Evaluations (55), a range of willingness-to-pay (WTP) thresholds, from USD 12,728–38,184 per QALY gained, that is, 1–3 times the gross domestic product (GDP) per capita. While domestic scholars have basically reached a consensus that the threshold limit of three times per capita GDP doesn't apply to China. Recently, Cai et al. (57) found the cost-effective threshold of a QALY in China was close to 1.5 times of GDP per capita (USD 19,091). Thus, in the base-case analysis, USD 19,091 was used to investigate whether alternative treatments were more cost-effective. National Institute for Health and Clinical Excellence (NICE) recommended multiplying the threshold level for end-stage disease treatment by a factor of 1.7, thus we used the cost-effective threshold of 2.55 times the GDP (USD 32,456) per capita in the subgroup analysis for second-line drugs (58). As recommended (55), costs and utilities were both discounted at an annual rate of 5% to reflect present values.

### Subgroup analysis

In addition to exploring the optimal treatment sequences, we also conducted subgroup analysis of the cost-effectiveness between first-line or second-line treatments. For the first-line subgroup, we compared seven treatments (standard chemotherapy, N + C, P + C, T + C, CA + C, SI + C or SU + C); For the second-line subgroup, we compared three treatments (nivolumab, tislelizumab, and docetaxel).

### Sensitivity analysis

Sensitivity analysis was performed to address the uncertainties in parameter values and decision making. We performed a one-way sensitivity analysis to test the sensitivity of results to changes in parameters such as costs, treatment effects, and utilities. Tornado graphs were plotted with the INMB used as a measure of cost-effectiveness to visualize the parameters which had a meaningful association with the conclusion. A Monte Carlo simulation was performed for 10,000 iterations for the probabilistic sensitivity analysis (PSA). The Gamma distribution was selected for cost, the Beta distribution for probability, proportion, and utilities, the Log-normal distribution was selected for the NMA shape or scale parameters. All the parameters were adjusted within the reported 95% confidence intervals or assuming reasonable ranges of the base-case values, details are provided in Table 1. A Scatter plot was drawn using the average cost and utility of 10,000 simulations for each therapy; cost-effectiveness acceptability curves were used to analyze the cost-effectiveness for each regimen with various cost-effective thresholds.

### Scenario analysis

To further explore the influence of parameter uncertainty and model structure on the research results, the following five scenarios were analyzed in this study.

Scenario 1: Using the utilities from Nafees et al. (54), the EQ-5D utilities were 0.804 for PFS and 0.321 for first- or end-stage PD.
Scenario 2: Using the utilities from Shen et al. (52), the EQ-5D utilities were 0.877 (immunotherapy) and 0.823 (chemotherapy) for PFS, 0.768 and 0.703 for first- or end-stage PD.
Scenario 3: Patient assistance programs (PAP) were considered for sugemalimab, nivolumab and pembrolizumab. Details are provided in Supplementary material 2.
Scenario 4: Considering the impacts of research time limits, longer simulation time frames, while closer to patients' lifetime costs and outcomes, also introduced more uncertainty. Therefore, we compared the costs and effects of each treatment when the simulation time was 5, 10 and 20 years.
Scenario 5: The cost of the third-line treatment the base-case analysis may be different from the actual clinical situation. For example, for patients with PS 0-1, third-line treatment with nivolumab or paclitaxel are recommended (21). We assumed that the cost of third-line treatment changed from USD 0–4,000 per cycle in this scenario.

## Results

### Network meta-analysis and survival rates estimates

**Figure 2:**
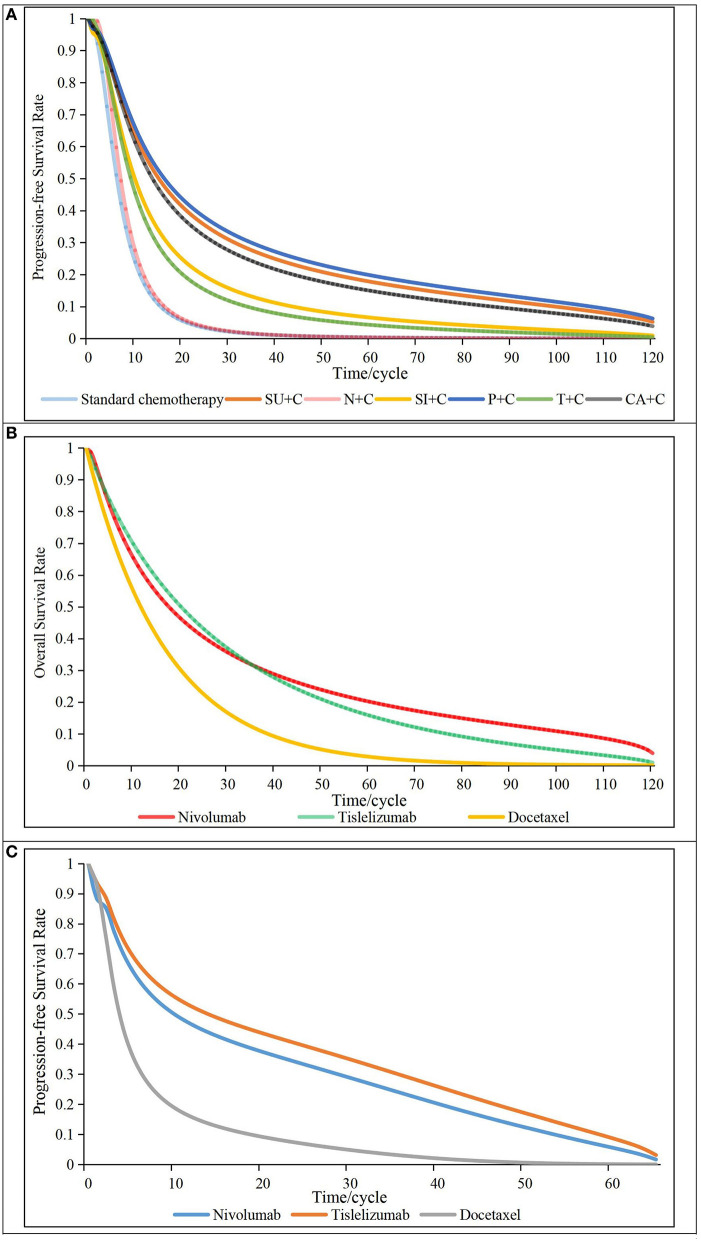
Survival curves of all first- or second-line treatments. **(A)** progressive-free survival curves for first-line treatments (above), **(B)** overall survival curves for second-line treatments (middle), **(C)** Progressive-free survival curves for second-line treatments (below), N + C, Nedaplatin in combination with standard chemotherapy; SI + C, Sintilimab in combination with standard chemotherapy; P + C, Pembrolizumab in combination with standard chemotherapy; T + C, Tislelizumab in combination with standard chemotherapy; CA + C, Camrelizumab in combination with standard chemotherapy; SU + C, Sugemalimab in combination with standard chemotherapy; cycle, 21 days.

A total of eight clinical trials with 2,154 patients were included in our NMA: Keynote-407 China, CameL-sq, Orient-12, Gemstone-302, Just and Rationale-304 for the first-line NMA (10–13, 16, 20); Checkmate-078 China and Rationale-303 for the other two second-line NMAs (23, 24, 46). Details for search strategies, network plot and risk of bias assessment are provided in Supplementary material 2, information of all RCTs are presented in Supplementary material 1. We chose the first-order FP models (*P* = −2) for the first-line NMA and the second-line NMA for PFS, second-order FP model (P_1_ = −0.5, P_2_ = 0) for the second-line NMA for OS. Related parameters for each intervention are listed in Table 1. The survival curves fitted by all models are provided in Supplementary material 2. The log-logistic model was chosen to reconstruct PFS curves of standard chemotherapy. The exponential distribution and restricted cubic spline models were used to fit the OS and PFS curves ‘of docetaxel, respectively. Details of the fitted survival curves for all treatments of the different models are provided in Supplementary material 2. AICs for parametric survival models are shown in Supplementary material 2. Other details for selecting parametric survival models are presented in Supplementary material 2. The PFS and OS curves of all first- or second-line treatments finally used in our model are presented in Figure 2.

### Model validation

The validation results showed that our model fitted and extrapolated well, and were consistent with clinical practice. Details results of model validation are presented in Supplementary material 2.

### Base-case analysis

The results of the base-case analysis are shown in Table 2. The mean QALYs for patients who received CD, ND, TID, SID, CT, NT, CN, NN, CAD, NN, CN, SUD or PED were 0.866, 0.906, 1.179, 1.266, 1.283, 1.301, 1.353, 1.389, 1.603, 1.721 and 1.807 ranked from least to most effective. The mean costs for patients who received ND, CD, SID, TID, NT, CT, CAD, NN, CN, SUD, and PED were USD 9,900, 9,981, 15,855, 16,072, 17,765, 18,131, 19,026, 61,498, 62,227, 80,927 and 117,369, ranked from least to most costly. Compared with ND, CD, SID, TID, NT and CT, the ICERs of CAD were USD 13,096, 12,276, 9,421, 6,974, 4,183, and 2,804 per QALY, all were < USD 19,091; and compared with NN and CN, CAD was cost-saving with improved effectiveness. The ICER of SUD and PED were USD 522,023 and 481,639 per QALY compared with CAD, respectively. Therefore, CAD was considered to be the most cost-effective treatment path for advanced sq-NSCLC, followed by SID, ND, NT, CD, TID, CT, NN, CN, SUD and PED in that order. Breakdown results of costs and utilities are shown in Supplementary material 3.

**Table 2:** Base-case analysis results.

**Treatment**	**ND**	**CD**	**SID**	**TID**	**NT**	**CT**	**CAD**	**NN**	**CN**	**SUD**	**PED**
Cost/$ (95% CI, discounted)	9,900 (9,775~10,030)	9,981 (9,859~10,109)	15,855 (15,706~16,018)	16,072 (15,910~16,244)	17,765 (17,542~18,003)	18,131 (17,906~18,372)	19,026 (18,846~19,228)	61,498 (60,393~62,625)	62,227 (61,102~63,376)	80,927 (79,959~81,919)	117,369 (115,979~118,785)
Utility/QALYs (95% CI, discounted)	0.906 (0.894~0.917)	0.866 (0.854~0.877)	1.266 (1.247~1.285)	1.179 (1.162~1.196)	1.301 (1.283~1.319)	1.283 (1.265~1.301)	1.603 (1.578~1.627)	1.389 (1.368~1.410)	1.353 (1.331~1.374)	1.721 (1.695~1.747)	1.807 (1.779~1.834)
Life-years/years (95% CI)	1.475 (1.454~1.495)	1.424 (1.404~1.445)	1.991 (1.960~2.021)	1.858 (1.830~1.885)	2.241 (2.207~2.274)	2.219 (2.185~2.253)	2.513 (2.473~2.554)	2.435 (2.393~2.476)	2.340 (2.338~2.421)	2.700 (2.658~2.743)	2.834 (2.789~2.879)
NMB/$ (95%CI, discounted)^¶^	7,391 (7,270~7,510)	6,548 (6,424~6,667)	8,315 (8,082~8,535)	6,436 (6,257~6,604)	7,073 (6,931~7,200)	6,368 (6,222~6,496)	1,1569 (11,247~11,871)	−3,4979 (-35928~-34,051)	−36,401 (-37370~-35,456)	−48,068 (-48703~-47,456)	−82,875 (-83902~-81,880)
INMB (VS. CAD)	−4,178	−5,022	−3,255	−5,134	−4,496	−5,201	NA	−46,548	−47,971	−59,637	−94,444
ICER	VS. ND	VS. CD	VS. SID	VS. TID	VS. NT	VS. CT	VS. CAD	VS. NN	VS. CN	VS. SUD	–
CD	dominated	–	–	–	–	–	–	–	–	–	–
SID	16,530^†^	14,677^†^	–	–	–	–	–	–	–	–	–
TID	22,590^§^	19,450^§^	−2486.48	–	–	–	–	–	–	–	–
NT	19,897^§^	17,884^†^	54,567	13,869^†^	–	–	–	–	–	–	–
CT	21,802^§^	19,522^§^	131,978	19,738^§^	dominated	–	–	–	–	–	–
CAD	13,096^†^	12,276^‡^	9,421^‡^	6,974^‡^	4,183^‡^	2,804^‡^	–	–	–	–	–
NN	106,754	98,453	370,975	216,196	496,744	409,928	dominated	–	–	–	–
CN	117,048	107,285	534,502	265,508	858,964	634,344	dominated	dominated	–	–	–
SUD	87,102	82,941	142,968	119,609	150,331	143,401	522,023	58,502	50,761	–	–
PED	119,270	114,123	187,726	161,344	196,940	189,562	481,639	133,753	121,458	425,698	–

^†^ < 1.5 times 2021 Gross Domestic Product (GDP) per capita ($19,092);^‡^1 times the 2021 GDP per capita ($12,728); ^§^ < 3 times the 2021 GDP per capita ($38,184); ^¶^Cost-effective threshold = 1.5 times the 2021 Gross Domestic Product per capita ($19,092). NMB, net monetary benefit (at the willingness to pay threshold of 1.5 times GDP per capita); INMB, increased net monetary benefit; NN, first-line nedaplatin-based chemotherapy followed by second-line nivolumab; NT, first-line nedaplatin-based chemotherapy followed by second-line tislelizumab; ND, first-line nedaplatin-based chemotherapy followed by second-line docetaxel; CN, first-line standard chemotherapy followed by second-line nivolumab; CT, first-line standard chemotherapy followed by second-line tislelizumab; CD, first-line standard chemotherapy followed by second-line docetaxel; TID, first-line tislelizumab combined with chemotherapy followed by second-line docetaxel; CAD, first-line camrelizumab combined with chemotherapy followed by second-line docetaxel; PED, first-line pembrolizumab combined with chemotherapy followed by second-line docetaxel; SID, first-line sintilimab combined with chemotherapy followed by second-line docetaxel; SUD, first-line sugemalimab combined with chemotherapy followed by second-line docetaxel; QALY, quality-adjusted life year; ICER, incremental cost-effectiveness ratio; NMB, net monetary benefit; INMB, Incremental net monetary benefit; CI, confidence interval.

### Subgroup analysis

#### Cost-effectiveness of first-line therapies

Compared with CA + C, the INMBs for the other 6 options were USD−3255 (SI + C),−4178 (N + C),−5134 (T + C),−47,971 (standard chemotherapy),−59,637 (SU + C) and−94,444 (P + C), from most cost-effective to least. Details are provided in Supplementary material 3. The NMB of CA + C was the largest among the seven treatments, which suggested that CA + C was the most cost-effective option.

#### Cost-effectiveness of second-line therapies

Tislelizumab with the largest NMB and QALYs among the three options was the most economical and effective second-line therapy for patients receiving either standard chemotherapy or nedaplatin in the first-line. Compared with docetaxel, the ICER of tislelizumab was about 1.5 times the GDP per capita per QALY, which was much smaller than that of nivolumab (USD 106,969/QALY). Other details are provided in Supplementary material 3.

### Sensitivity analysis

#### One-way sensitivity analysis

**Figure 3:**
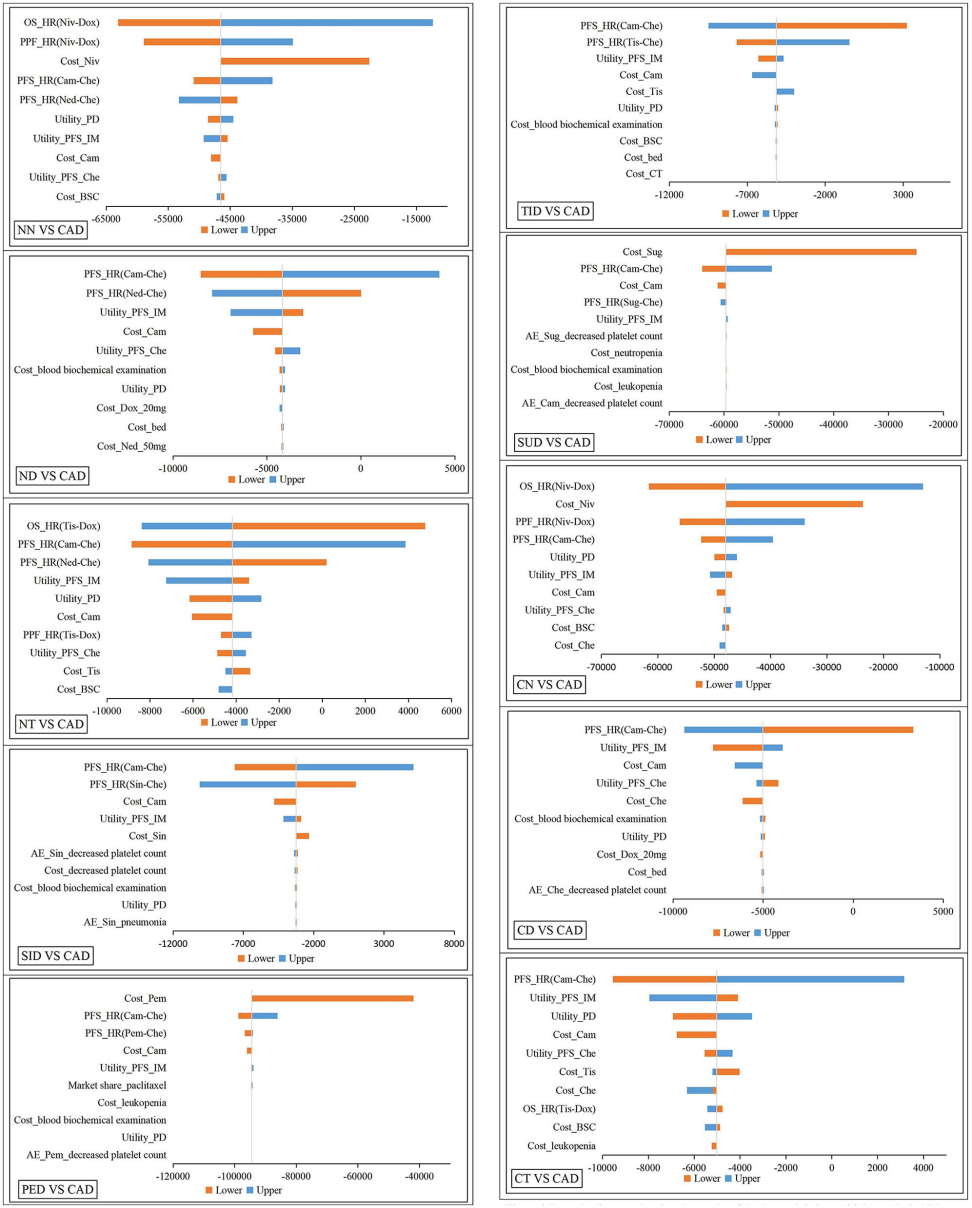
Tornado diagram showing the results of the deterministic sensitivity analysis. NN, first-line nedaplatin-based chemotherapy followed by second-line nivolumab; NT, first-line nedaplatin-based chemotherapy followed by second-line tislelizumab; ND, first-line nedaplatin-based chemotherapy followed by second-line docetaxel; CN, first-line standard chemotherapy followed by second-line nivolumab; CT, first-line standard chemotherapy followed by second-line tislelizumab; CD, first-line standard chemotherapy followed by second-line docetaxel; TID, first-line tislelizumab combined with chemotherapy followed by second-line docetaxel; CAD, first-line camrelizumab combined with chemotherapy followed by second-line docetaxel; PED, first-line pembrolizumab combined with chemotherapy followed by second-line docetaxel; SID, first-line sintilimab combined with chemotherapy followed by second-line docetaxel; SUD, first-line sugemalimab combined with chemotherapy followed by second-line docetaxel; HR, hazards rations; OS, overall survival; PFS, progression-free survival; PD, progression disease; Cam, camrelizumab; Tis, tislelizumab; Niv, nivolumab; Che, Chemotheraphy; Dox, docetaxel; BSC, best support care; IM, immunotheraphy; Sug, sugemalimab; Sin, sintilimab; Pem, pembrolizumab; CT, computed tomography; AE, adverse events.

Selecting the most economical CAD as the reference, we made tornado graphs of the other 10 treatment sequences (Figure 3). Although each parameter fluctuated, the NMBs of CAD were always larger compared with NN, CN, PED and SUD. Only when the HRs for PFS of CA + C fluctuated, CD, ND, CT and TID were likely to be more cost-effective. The cost-effectiveness of CAD and SID were affected by HRs for PFS of CA + C and SI + C, and cost-effectiveness of CAD and NT were affected by HRs for PFS of CA + C and OS of tislelizumab. One-way sensitivity analysis indicated that the HRs and costs of immunotherapy drugs had the greatest impacts on the INMBs, but overall, the base-case analysis results were relatively stable.

#### Probabilistic sensitivity analysis

**Figure 4:**
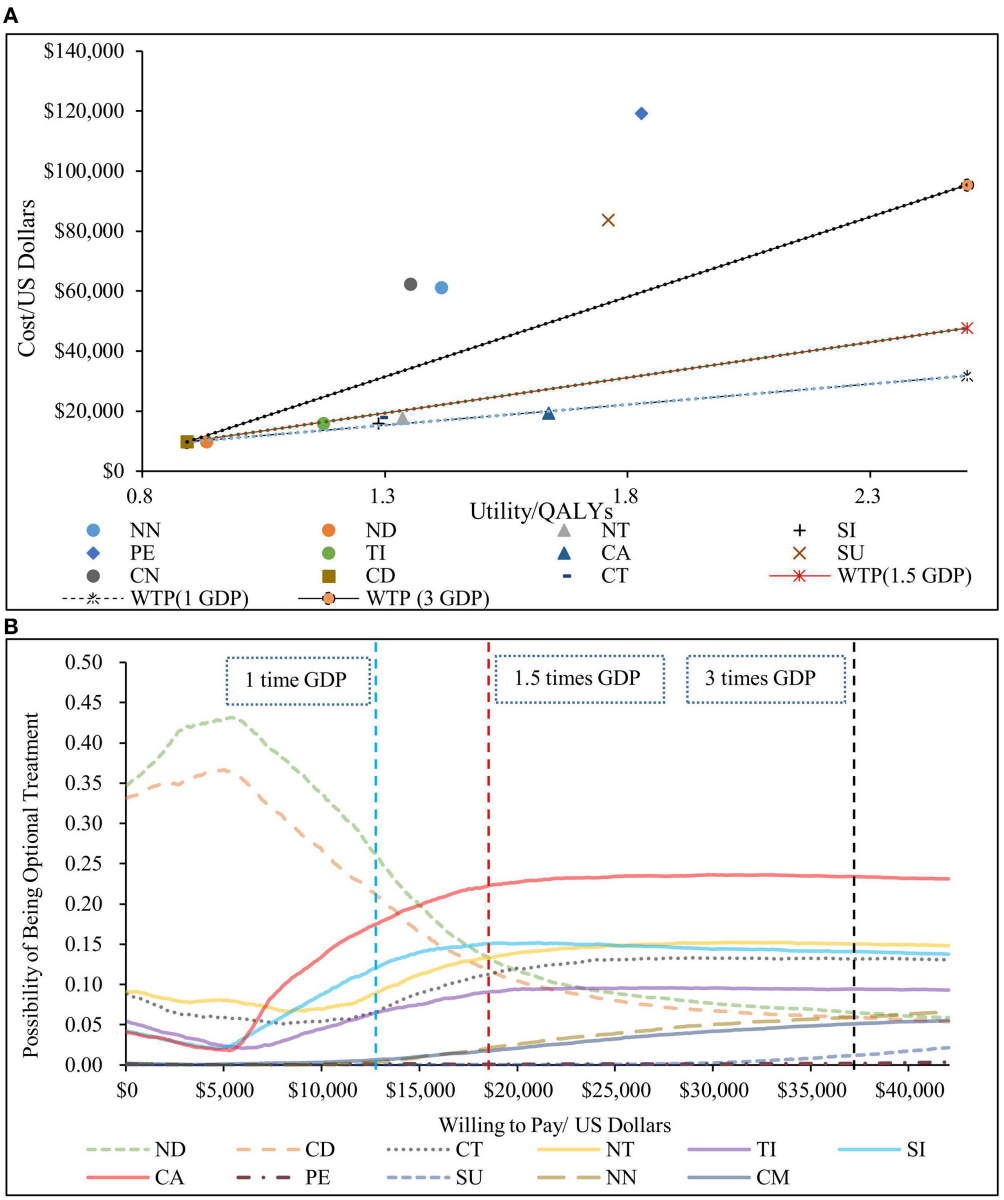
Results of the probabilistic sensitivity analysis. **(A)** scatter plot (above). **(B)** cost-effectiveness acceptable curve (below). NN, first-line nedaplatin-based chemotherapy followed by second-line nivolumab; NT, first-line nedaplatin-based chemotherapy followed by second-line tislelizumab; ND, first-line nedaplatin-based chemotherapy followed by second-line docetaxel; CN, first-line standard chemotherapy followed by second-line nivolumab; CT, first-line standard chemotherapy followed by second-line tislelizumab; CD, first-line standard chemotherapy followed by second-line docetaxel; CA, first-line camrelizumab combined with chemotherapy followed by second-line docetaxel; PE, first-line pembrolizumab combined with chemotherapy followed by second-line docetaxel; SI, first-line sintilimab combined with chemotherapy followed by second-line docetaxel; SU, first-line sugemalimab combined with chemotherapy followed by second-line docetaxel; QALY, quality-adjusted life year; GDP, 2021 per capita Gross Domestic Product.

The results of the PSA are shown in Figure 4. The scatter plot showed that NN, CN, SU and PE were not cost-effective even when cost-effective threshold was three times the GDP per capita compared to CD; the ICERs of the other six treatment sequences (ND, TID, SID, NT, CT, and CAD) were below the chosen cost-effective threshold compared to CD. Compared with the other six treatments, the ICERs of CAD were all much smaller than the chosen cost-effective threshold. According to the cost-effectiveness acceptability curves, ND was the most economical option when cost-effective threshold was lower than USD 15,000, and CAD was the most economical therapy when cost-effective threshold was over USD 15,000. Under the chosen threshold, CAD was the optimal choice in cost-effectiveness. These results confirmed that the conclusions of our study were sufficiently reliable.

### Scenario analysis

Results of Scenarios 1–4 are concluded in Supplementary material 3. In the first two scenarios, when utilities changed, the ICERs of CAD compared with economically suboptimal ND and SID both became smaller, even < 2,021 GDP per capita. After considering PAP, cost of sugemalimab, nivolumab, or pembrolizumab was all much lower, ICER of nivolumab, sugemalimab or pembrolizumab was $40,726, 34,094, and 24,499 compared to the ND, while still exceeded the selected cost-effective threshold. When the study time frame was reduced to 5 or 10 years, the ICER for CAD compared to ND increased slightly, but overall results were similar to those of 20 years. According to Supplementary material 3, CAD was always the most cost-effectiveness option over time. Results of Scenario 5 (Supplementary material 3) showed that the cost of third-line therapy did not affect the cost-effectiveness of CAD.

## Discussion

We explored the effectiveness and cost-effectiveness of different regimens for advanced sq-NSCLC treatment. According to the recommendation of CSCO 2022, 11 treatment sequences (ND, NN, NT, CD, CT, CN, TID, CAD, PED, SUD, and SID) are available for patients with advanced sq-NSCLC. We evaluated the effectiveness and cost-effectiveness of these treatment from the perspective of Chinese healthcare system using a sequential model. We found that regardless of using in the first- or second-line, immunotherapy would bring higher cost but more survival benefits to patients than chemotherapy. The base-case results showed that PED was the most effective option, but CAD was the optimal choice in cost-effectiveness under the chosen cost-effective threshold of 1.5 times the GDP per capita. Compared with suboptimal therapies, ND and SID, the ICERs of CA + C + D were USD 13,096 and 9,421 per QALY, respectively. Both one-way and probabilistic sensitivity analyses confirmed that the results were sufficiently reliable, CAD was the most cost-effective therapy when this is not a commonly used acronym in health economics. was over USD 15,000. Scenario analysis showed that CAD was always the most cost-effective, regarless of the changes in utilities, study duration, PAP, and cost of third-line treatment.

Subgroup results showed that P + C was the most effective, while CA + C was the most cost-effective among seven first-line therapies. Tislelizumab was the best second-line choice compared to nivolumab and docetaxel both in effectiveness and cost-effectiveness.

PED and SUD were the most effective treatment sequences, which could bring 1.807 and 1.721 QALYs to patients, respectively. But pembrolizumab and sugemalimab were cost-effective compared to CAD only after a price reduction of 90 and 85% respectively. Keynote-407 China (13) was chosen as the source of the efficacy of P + C in this China-based research. Compared with the global population (14), the performance of P + C in the Chinese population improved a lot, which was the reason why P + C was so effective in this study.

No studies targeted on the cost-effectiveness of treatment sequences for advanced sq-NSCLC in China have been published so far. Cheng et al. (22) explored the cost-effectiveness of atezolizumab compared with chemotherapy in treating NSCLC patients with PD-L1 expression levels >50%. The authors concluded that atezolizumab had better efficacy but was not cost-effective. Teng et al. (59) compared nivolumab, pembrolizumab, atezolizumab, and durvalumab in first-line treatment of NSCLC patients with high PD-L1 expression. The effectiveness and cost-effectiveness of nivolumab were found to be similar among various immune checkpoint inhibitors, but nivolumab was the most economical. Hao et al. (60) showed that nivolumab combined with ipimumab was not cost-effective compared with chemotherapy in advanced EGFR or ALK mutation-negative NSCLC. Wu et al. (61) evaluated the combination of pembrolizumab with chemotherapy and chemotherapy in patients with EGFR or ALK mutation-negative sq-NSCLC, and showed that the combination regimen was not cost-effective regardless of the PD-L1 expression level. Liao et al. (62) further confirmed from the perspective of the whole society that pembrolizumab was not economical compared to chemotherapy for PD-L1 High-expressing NSCLC. Further information of a systematic review of current published CEA based in China is provided in Supplementary material 1.

Sintilimab, camrelizumab and tislelizumab have been included in the NRDL since 2020, which meant that the prices of these drugs had greatly reduced, thereby improving the cost-effectiveness of combination therapy (41, 43). Camrelizumab combined with chemotherapy for first-line treatment or and tislelizumab for second-line treatment of advanced sq-NSCLC is likely to be listed in the NRDL based on the results of CameL-sq and Rationale-303 (11, 46). As the prices of camrelizumab and tislelizumab were unclear for sq-NSCLC, we considered a wide range of prices, and the sensitivity analysis results showed that the prices did not affect the conclusion.

### Strengths and limitations

Firstly, effectiveness and cost-effectiveness of seven first-line treatments, three second-line treatments and 11 treatment sequences for advanced sq-NSCLC approved in China were systematically compared for the first time. This study is important for patients, clinicians, and payers given the uncertainty about the optimal treatment for advanced sq-NSCLC, which causes serious morbidity and mortality in China. Our cost-effectiveness analysis provides information that can provide value-based decision-making evidence for the Chinese healthcare system. In the upcoming 2022 NRDL negotiation, our research may provide comprehensive and scientific evidence for drugs access negotiation for the treatment of wild-type advanced sq-NSCLC. Secondly, we constructed the NMA based on the FP model, and calculated time-varying HRs as non-PH were detected in the chosen trials. PH assumption has been used blindly without verification in previous studies, but actually this assumption is difficult to hold in NMA composed of multiple comparisons and serious survival fitting bias would be caused when PH models are used in case of PH assumption does not hold. Thirdly, we used a micro-simulation model that allows transition rates to vary over time under the time-reset option. Compared with memoryless hypothesis Markov cohort model, our model better simulated the long-term survival of patients. Finally, through sensitivity analysis and scenario analysis, we have fully explored the influences of parameter uncertainty and model structure on the results.

Our model includes several simplifying assumptions that limit its application. Firstly, to estimate progression rates, we synthesized survival data from multiple clinical trial populations. This introduced some uncertainty because no one trial population followed the treatment regimens specified in our model. Secondly, efficacy of docetaxel in patients receiving first-line immunotherapy is not yet available, and we assumed the efficacy of these patients were the same as receiving SC in first-line. According to the results of Checkmate 057 (63) and a real-world study (64), the median OS of advanced non-squamous NSCLC patients receiving docetaxel after standard chemotherapy was 9.5 (8.1–10.7) months, and the median OS of patients receiving docetaxel after treatment with immunotherapy combined with chemotherapy was 9.0 (8.1–11.2) months, thus, the efficacy of docetaxel was nearly identical whether received treatment with immunotherapy combined with chemotherapy or standard chemotherapy in first-line, and we considered our assumptions to be reasonable. Thirdly, there is no direct head-to-head evidence for the relative efficacy of N + C, P + C, SI + C, SU + C, CA + C and TI + C, and no direct evidence for the relative efficacy of tislelizumab and nivolumab, although we identified and used the best NMA model, some uncertainty remains. Fourthly, PFS rates of some first-line treatments such as SI + C and TI + C were relatively immature, parametric extrapolation would bring certain uncertainties. Fifthly, because the tail data of the PFS curves in the second-line docetaxel group were too sparse, the HRs calculated in the model were relatively small, which in turn caused the efficacy of tislelizumab and nivolumab to be slightly overestimated. Finally, toripalimab and penpulimab were not considered in our model, as they are second-level recommended by CSCO 2022 and have not yet been approved for treatment of sq-NSCLC in China as of May 2022.

## Conclusion

We provided a novel sequential model to determine the optimal therapeutic pathway as certain reference for future research. Although PED is currently the most effective therapy, CAD is the most cost-effective treatment sequence among 11 options. P + C and CA + C is the most effective and cost-effective therapy in first-line, respectively; tislelizumab is the best second-line choice. Our results may help clinicians make optimal decisions in treating advanced sq-NSCLC and provide value-based evidence for decision-making for the Chinese healthcare system. However, long-term follow-up data and direct-comparison evidence are still needed to confirm the results.

## Data availability statement

The original contributions presented in the study are included in the article/Supplementary material, further inquiries can be directed to the corresponding author.

## Author contributions

WT, MZ, and TS: full access to all of the data in the study, take responsibility for the integrity of the data, the accuracy of the data analysis, and concept and design. MZ, TS, and ZC: acquisition of data. MZ: analysis and interpretation of data. WT, MZ, TS, and ZC: critical revision of the manuscript for important intellectual content. MZ and TS: statistical analysis and drafting of manuscript. WT: obtaining funding, administrative and technical support, and supervision. All authors contributed to the article and approved the submitted version.

## Glossary

AE: Adverse Reaction
AIC: Akaike Information Criterion
BSC: Best Supportive Treatment
CHEERS: Consolidated Health Economic Evaluation Reporting Standards
CSCO 2022: Chinese Society of Clinical Oncology Guidelines 2022
EORTC QLQ-C30: European Organization for Research and Treatment Quality of Life Questionnaire-Core 30
EQ-5D: Euroqol-5-Dimension
EQ-5D-5L: Euroqol-5-Dimension-5 Level
CAD: First-Line Ca + C Followed By Second-Line Docetaxel
ND: First-Line N + C Followed By Second-Line Docetaxel
NN: First-Line N + C Followed By Second-Line Nivolumab
NT: First-Line N + C Followed By Second-Line Tislelizumab
PED: First-Line P + C Followed By Second-Line Docetaxel
SID: First-Line Si + C Followed By Second-Line Docetaxel
CD: First-Line Standard Chemotherapy Followed By Second-Line Docetaxel
CN: First-Line Standard Chemotherapy Followed By Second-Line Nivolumab
CT: First-Line Standard Chemotherapy Followed By Second-Line Tislelizumab
SUD: First-Line Su + C Followed By Second-Line Docetaxel
TID: First-Line T + C Followed By Second-Line Docetaxel
FP: Fractional Polynomial
GDP: Gross Domestic Product
HR: Hazard Ratio
ICER: Incremental Cost-Effectiveness Ratio
INMB: Incremental Net Monetary Benefit
NRDL: National Reimbursement Drug List
N + C: Nedaplatin Combined With Docetaxel
NMA: Network Meta-Analysis
NSCLC: Non-Small Cell Lung Cancer
non-sq: Non-Squamous
OS: Overall Survival
CA+C: Paclitaxel And Platinum Combined With Camrelizumab
P + C: Paclitaxel And Platinum Combined With Pembrolizumab
SU + C: Paclitaxel And Platinum Combined With Sugemalimab
T + C: Paclitaxel And Platinum Combined With Tislelizumab
PAP: Patient Assistance Program
PS: Performance Status
SC: Platinum- And Paclitaxel-Based Chemotherapy
SI + C: Platinum Combined With Gemcitabine And Sintilimab
PD-1: Programmed Death-1
PD-L1: Programmed Death-Ligand 1
PD: Progressed Disease
PFS: Progression-Free Survival
PH: Proportional Hazard
QALY: Quality-Adjusted Life Year
RCT: Randomized Clinical Trial
RP: Royston and Parmar
sq: Squamous
USD: United States Dollars
WTP: Willingness-To-Pay.
