# Supplementary material for: Effectiveness and cost-effectiveness analysis of 11 treatment paths, seven first-line and three second-line treatments for Chinese patients with advanced wild-type squamous non-small cell lung cancer: A sequential model
Source: Front Public Health. 2023 Feb 24;11:1051484. doi: 10.3389/fpubh.2023.1051484 (PMC9999022; doi:10.3389/fpubh.2023.1051484)
Supplement: Supplementary file 1 [file Data_Sheet_1.DOCX]

**Supplement 1**

[eTable 1 Information of Included RCTs, Medication Dosages and Treatment Duration. 1](#_Toc26063)

[eTable 2 Resource Utilization 5](#_Toc30468)

[eTable 3 Review of Pharmacoeconomic Evaluation 6](#_Toc20838)

### eTable 1 Information of Included RCTs, Medication Dosages and Treatment Duration.

| Study | Treatment | | Stage | Sample size | Mean age | Sex (male proportions) | Region | Smoking status (non-smoking proportions) |
| --- | --- | --- | --- | --- | --- | --- | --- | --- |
|  | Intervention (arm 1) | Control (arm 2) |  | arm 1 VS arm 2 | arm 1 VS arm 2 | arm 1 VS arm 2 | arm 1&2 | arm 1 VS arm 2 |
| Keynote-407 China^[1]^ | Pembrolizumab (200mg/21 days) + control arm | Paclitaxel (200mg/m^2^/21 days) or Nab-paclitaxel (100mg/m^2^/21 days) + Carboplatin (AUC 6 mg/mL/min/21 days) | Ⅳ | 65 VS 60 | 63 VS 63 | 95% VS 95% | Chinese | 8% VS 10% |
| Gemstone-302^[2]^ | Sugemalimab (1200mg/21 days) + control arm | Paclitaxel (200mg/m^2^/21 days) + Carboplatin (AUC 6 mg/mL/min/21 days) | Ⅳ | 62 VS 64 | 62 VS 64 | 79% VS 81% | Chinese | 27% VS 25% |
| Orient-12^[3]^ | Sintilimab (200mg/21 days) + control arm | Gemcitabine (100mg/m^2^/21 days) and either Cisplatin (75 mg/m^2^/21 days) or Carboplatin (AUC 5 mg/mL/min/21 days) + placedo | ⅢB-Ⅳ | 179 VS 178 | 64 VS 62 | 91% VS 92% | Chinese | 13% VS 17% |
| CameL-sq^[4]^ | Camrelizumb (200mg/21 days) + control arm | Carboplatin (AUC 5 mg/mL/min/21 days) and Paclitaxel (200mg/m^2^/21 days) | ⅢB-Ⅳ | 193 VS 196 | 64 VS 62 | 93% VS 92% | Chinese | 11% VS 12% |
| Rationale-307^[5]^ | Tislelizumab (200mg/21 days) + control arm | Carboplatin (AUC 5 mg/mL/min/21 days) and Paclitaxel (175mg/m^2^/21 days) | ⅢB-Ⅳ | 120 VS 121 | 60 VS 62 | 89% VS 92% | Chinese | 20% VS 19% |
| Just^[6]^ | Nedaplatin (80mg/m2/21 days) + docetaxel (75mg/m2/21 days) | Cisplatin + Docetaxel (75mg/m^2^/21 days) | ⅢB-Ⅳ | 141 VS 139 | 62 VS 61 | 87% VS 89% | Chinese | NA VS NA |
| Rationale-303^[7]^ | Tislelizumab (200mg/21 days) | Docetaxel (75mg/m^2^/21 days) | Ⅳ | 248 VS 122 | 62 VS 63 | 92% VS 91% | Chinese | 14% VS 11% |
| Checkmate-078 China^[8, 9]^ | Nivolumab (3mg/kg/14 days) | Docetaxel (75mg/m^2^/21 days) | Ⅳ | 338 VS 166 | 60 VS 60 | 78% VS 81% | Chinese | 30% VS 29% |
| Study | PD-L1 status(＜1%/≥1%/1-49%/≥50%) | | HR(PFS) | HR(OS) | SAE/ Total. (%) | Median PFS (month) | Median OS (month) | |
|  | arm 1 | arm 2 | arm 1 VS arm 2 | arm 1 VS arm 2 | arm 1 VS arm 2 | arm 1 VS arm 2 | arm 1 VS arm 2 | |
| Keynote-407 China^[1]^ | 95/176/103/73 | 99/177/104/73 | 0.57(0.47-0.69) | 0.71(0.58-0.88) | 74% VS 70% | 8.0 VS 5.1 | 17.1 VS 11.6 | |
| Gemstone-302^[2]^ | 124/196/NA/NA | 64/95/NA/NA | 0.34(0.24-0.48) | 0.48(0.31-0.74) | 64% VS 62% | 8.3 VS 4.8 | 23.3 VS 12.2 | |
| Orient-12^[3]^ | 59/120/62/58 | 63/115/52/63 | 0.536(0.422-0.681) | 0.567(0.353-0.909) | 87% VS 82% | 6.7 VS 4.9 | NA VS NA | |
| CameL-sq^[4]^ | 91/95/58/37 | 97/93/49/44 | 0.37(0.29-0.47) | 0.57(0.44-0.74) | 74% VS 72% | 8.5 VS 4.9 | 27.4 VS 14.5 | |
| Rationale-307^[5]^ | 48/NA/30/42 | 49/NA/31/41 | 0.52(0.37-0.74) | NA | 88% VS 84% | 7.6 VS 5.5 | NA VS NA | |
| Just | NA | NA | 0.778(0.599-1.009) | NA | 33% VS 45% | 4.6 VS 4.2 | NA VS NA | |
| Rationale-303^[7]^ | <25%:134; $\geq$25%:114 | <25%:66; $\geq$25%:56 | 0.78(0.64-0.96) | 0.58(0.44-0.76) | 30% VS 39% | 6.2 VS 2.8 | 16.0 VS 11.3 | |
| Checkmate 078 China^[8, 9]^ | 138/168/NA/NA | 67/84/NA/NA | 0.78(0.64-0.96) | 0.65(0.47-0.89) | 12% VS 47% | 2.8 VS 2.8 | 11.7 VS 7.9 | |
| Study | Treatment Duration^*^ | | | | | | Patients | |
|  | arm 1 | | | arm 2 | | | arm 1 & arm 2 | |
| Keynote-407 China^[1]^ | Pembrolizumab: continue medication until disease progression or unacceptable toxicity, a maximum of 2 years  Chemotherapy: same as the control group | | | Chemotherapy: continue medication until disease progression or unacceptable toxicity, a maximum of 4 cycles | | | Advanced wild-type squamous non-small cell lung cancer, without previous systemic therapy | |
| Gemstone-302^[2]^ | Sugemalimab: continue medication until disease progression or unacceptable toxicity, a maximum of 2 years  Chemotherapy: same as the control group | | | Chemotherapy: continue medication until disease progression or unacceptable toxicity, a maximum of 4-6 cycles^#^ | | | Advanced wild-type squamous non-small cell lung cancer, without previous systemic therapy | |
| Orient-12^[3]^ | Sintilimab: continue medication until disease progression or unacceptable toxicity, a maximum of 2 years  Chemotherapy: same as the control group | | | Chemotherapy: continue medication until disease progression or unacceptable toxicity, a maximum of 4-6 cycles^#^ | | | Advanced wild-type squamous non-small cell lung cancer, without previous systemic therapy | |
| CameL-sq^[4]^ | Camrelizumb: continue medication until disease progression or unacceptable toxicity, a maximum of 2 years  Chemotherapy: same as the control group | | | Chemotherapy: continue medication until disease progression or unacceptable toxicity, a maximum of 4-6 cycles^#^ | | | Advanced wild-type squamous non-small cell lung cancer, without previous systemic therapy | |
| Rationale-307^[5]^ | Tislelizumab: continue medication until disease progression or unacceptable toxicity, a maximum of 2 years  Chemotherapy: same as the control group | | | Chemotherapy: continue medication until disease progression or unacceptable toxicity, a maximum of 4-6 cycles^#^ | | | Advanced wild-type squamous non-small cell lung cancer, without previous systemic therapy | |
| Just^[6]^ | Nedaplatin: continue medication until disease progression or unacceptable toxicity, a maximum of 2 years  Chemotherapy: same as the control group | | | Chemotherapy: continue medication until disease progression or unacceptable toxicity, a maximum of 4 cycles | | | Advanced wild-type squamous non-small cell lung cancer, without previous systemic therapy | |
| Rationale-303^[7]^ | Tislelizumab: continue medication until disease progression or unacceptable toxicity | | | Docetaxel: continue medication until disease progression or unacceptable toxicity | | | Advanced wild-type squamous non-small cell lung cancer, and disease progression after platinum-doublet chemotherapy | |
| Checkmate 078 China^[8, 9]^ | Nivolumab: continue medication until disease progression or unacceptable toxicity | | | Docetaxel: continue medication until disease progression or unacceptable toxicity | | | Advanced wild-type squamous non-small cell lung cancer, and disease progression after platinum-doublet chemotherapy | |
| *Time to treatment discontinuation was assumed in our model to be equal to time spent in the “pre-progression” health state, the clinical benefit associated with the treatment was assumed to remain unchanged following this stopping rule^[10]^  #Four cycles were assumed in our model; | | | | | | | | |

### eTable 2 Resource Utilization

| Medical sources | Resource utilization (times/21 days） |
| --- | --- |
| Diagnosis | 1 |
| Intravenous injection | 3 |
| Nursing | PFS：3；PD：2 |
| Bed | 3 |
| CT examination | PFS：1~4 cycle：0.5；5~12 cycle：1/3；after 12 cycles：0.25；PD：0.25 |
| Blood biochemical examination | PFS：1；PD：0.75 |
| Blood test | PFS：1；PD：0.75 |
| Urinalysis | PFS：1；PD：0.75 |

PFS, progression-free survival; PD, progressive disease.

### eTable 3 Review of Pharmacoeconomic Evaluation

Cheng et al. ^[11]^ explored the cost-effectiveness of atezolizumab compared with chemotherapy in treating NSCLC patients with PD-L1 expression levels >50%. The authors concluded that atezolizumab had better efficacy but was not cost-effective. Teng et al. ^[12]^ compared nivolumab, pembrolizumab, atezolizumab, and durvalumab in first-line treatment of NSCLC patients with high PD-L1 expression. The effectiveness and cost-effectiveness of nivolumab were found to be similar among various immune checkpoint inhibitors, but nivolumab was the most economical. Hao et al. ^[13]^ showed that nivolumab combined with ipimumab was not cost-effective compared with chemotherapy in advanced EGFR or ALK mutation-negative NSCLC. Wu et al. ^[14]^ evaluated the combination of pembrolizumab with chemotherapy and chemotherapy in patients with EGFR or ALK mutation-negative sq-NSCLC, and showed that the combination regimen was not cost-effective regardless of the PD-L1 expression level. Liao et al. ^[15]^ further confirmed from the perspective of the whole society that pembrolizumab was not economical compared to chemotherapy for PD-L1 High-expressing NSCLC.

| Study | Perspective | Patients | Comparisons | Model | Health states | Study time | Threshold of WTP | Cycle length | Discount | Source of clinical data | Source of utilities | Health outcomes |
| --- | --- | --- | --- | --- | --- | --- | --- | --- | --- | --- | --- | --- |
| Cheng-2021^[11]^ | Chinese payer | NSCLC patients with PD-L1 ≥ 50% | Atezolizumab compared to chemotherapy | Partitioned survival model | PFS, PD, Death | 20 years | 3 times GDP per capita | NA | 3% | IMpower110 | published articles | QALY |
| Teng-2021^[12]^ | Chinese healthcare system | NSCLC patientswith high PD‐L1 expression | Nivolumab, pembrolizumab, atezolizumab, and durvalumab | Markov model | PFS, PD, Death | Lifetime | 3 times GDP per capita | 21 days | 5% | KEYNOTE‐024, KEYNOTE‐042, CheckMate‐026, IMpower 110, and MYSTIC | published articles | QALY |
| Hao-2021^[13]^ | Chinese health care perspectives | Advanced wild-type NSCLC | Nivolumab plus ipilimumab compared with chemotherapy | Markov model | PFS, PD, Death | 10 years | 3 times GDP per capita | 7 days | 5% | CheckMate-227 | published articles | QALY |
| Wu-2020^[14]^ | Chinese health care perspectives | Metastatic wild-type nonsquamous and squamous NSCLC | pembrolizumab plus chemotherapy compared with chemotherapy | Markov model | PFS, PD, Death | 20 years | 3 times GDP per capita | 21 days | 5% | KEYNOTE-189 and KEYNOTE-407 | published articles | QALY |
| Liao-2019^[15]^ | Chinese societal perspective | advanced NSCLC patients with PD-L1 positive | pembrolizumab compared with chemotherapy | Markov model | PFS, PD, Death | 10 years | 3 times GDP per capita | 21 days | 3% | KEYNOTE-024 | published articles | QALY |

PFS, progression-free survival; PD, progressive survival; QALY, quality-adjusted life year; PD-L1, programmed death-ligand 1; NSCLC, Non-small cell lung cancer

**Reference**

[1] Cheng Y, Zhang L, Hu J, et al. Pembrolizumab Plus Chemotherapy for Chinese Patients With Metastatic Squamous NSCLC in KEYNOTE-407[J]. JTO Clin Res Rep, 2021,2(10):100225.

[2] Zhou C, Wang Z, Sun Y, et al. Sugemalimab versus placebo, in combination with platinum-based chemotherapy, as first-line treatment of metastatic non-small-cell lung cancer (GEMSTONE-302): interim and final analyses of a double-blind, randomised, phase 3 clinical trial[J]. Lancet Oncol, 2022,23(2):220-233.

[3] Zhou C, Wu L, Fan Y, et al. Sintilimab Plus Platinum and Gemcitabine as First-Line Treatment for Advanced or Metastatic Squamous NSCLC: Results From a Randomized, Double-Blind, Phase 3 Trial (ORIENT-12)[J]. J Thorac Oncol, 2021,16(9):1501-1511.

[4] Ren S, Chen J, Xu X, et al. Camrelizumab Plus Carboplatin and Paclitaxel as First-Line Treatment for Advanced Squamous NSCLC (CameL-Sq): A Phase 3 Trial[J]. J Thorac Oncol, 2022,17(4):544-557.

[5] Wang J, Lu S, Yu X, et al. Tislelizumab Plus Chemotherapy vs Chemotherapy Alone as First-line Treatment for Advanced Squamous Non-Small-Cell Lung Cancer: A Phase 3 Randomized Clinical Trial[J]. JAMA Oncol, 2021,7(5):709-717.

[6] Lu S, Chen Z, Hu C, et al. Nedaplatin plus docetaxel versus cisplatin plus docetaxel as first-line chemotherapy for advanced squamous cell carcinoma of the lung—a multicenter, open-label, randomized, phase III trial[J]. Journal of Thoracic Oncology, 2018,13(11):1743-1749.

[7] Caicun Zhou D H X Y. Results from RATIONALE 303: A global Phase 3 study of tislelizumab vs docetaxel as second- or third-line therapy for patients with locally advanced or metastatic NSCLC. AACR 2021 CT039.[C].

[8] Chang J, Wu Y L, Lu S, et al. Three-year follow-up and patient-reported outcomes from CheckMate 078: Nivolumab versus docetaxel in a predominantly Chinese patient population with previously treated advanced non-small cell lung cancer[J]. Lung Cancer, 2021,165:71-81.

[9] Lu S, Wang J, Cheng Y, et al. Nivolumab versus docetaxel in a predominantly Chinese patient population with previously treated advanced non-small cell lung cancer: 2-year follow-up from a randomized, open-label, phase 3 study (CheckMate 078)[J]. Lung Cancer, 2021,152:7-14.

[10] Kuznik A, Smare C, Chen C I, et al. Cost-Effectiveness of Cemiplimab Versus Standard of Care in the United States for First-Line Treatment of Advanced Non-small Cell Lung Cancer With Programmed Death-Ligand 1 Expression ≥50[J]. Value Health, 2022,25(2):203-214.

[11] Cheng S, Pei R, Li J, et al. Atezolizumab compared to chemotherapy for first-line treatment in non-small cell lung cancer with high PD-L1 expression: a cost-effectiveness analysis from US and Chinese perspectives[J]. Ann Transl Med, 2021,9(18):1481.

[12] Teng M M, Chen S Y, Yang B, et al. Determining the optimal PD-1/PD-L1 inhibitors for the first-line treatment of non-small-cell lung cancer with high-level PD-L1 expression in China[J]. Cancer Med, 2021,10(18):6344-6353.

[13] Hao X, Shen A, Wu B. Cost-Effectiveness of Nivolumab Plus Ipilimumab as First-Line Therapy in Advanced Non-small-cell Lung Cancer[J]. Front Pharmacol, 2021,12:573852.

[14] Wu B, Lu S. The effect of PD-L1 categories-directed pembrolizumab plus chemotherapy for newly diagnosed metastatic non-small-cell lung cancer: a cost-effectiveness analysis[J]. Transl Lung Cancer Res, 2020,9(5):1770-1784.

[15] Liao W, Huang J, Hutton D, et al. Cost-effectiveness analysis of first-line pembrolizumab treatment for PD-L1 positive, non-small cell lung cancer in China[J]. J Med Econ, 2019,22(4):344-349.
