# Supplementary material for: Effectiveness and cost-effectiveness analysis of 11 treatment paths, seven first-line and three second-line treatments for Chinese patients with advanced wild-type squamous non-small cell lung cancer: A sequential model
Source: Front Public Health. 2023 Feb 24;11:1051484. doi: 10.3389/fpubh.2023.1051484 (PMC9999022; doi:10.3389/fpubh.2023.1051484)
Supplement: Supplementary file 2 [file Data_Sheet_2.PDF]

Supplement 4

The CHEERS 2022 Checklist.

Section/topic	Item No	Guidance for reporting	Reported in section
**Title**			
Title	1	Identify the study as an economic evaluation and specify the interventions being compared.	P1
**Abstract**			
Abstract	2	Provide a structured summary that highlights context, key methods, results, and alternative analyses.	P1-2
**Introduction**			
Background and objectives	3	Give the context for the study, the study question, and its practical relevance for decision making in policy or	P3-4
**Methods**			
Health economic analysis plan	4	Indicate whether a health economic analysis plan was developed and where available.	P5
Study population	5	Describe characteristics of the study population (such as age range, demographics, socioeconomic, or clinical	P4
Setting and location	6	Provide relevant contextual information that may influence findings.	P5
Comparators	7	Describe the interventions or strategies being compared	P6
Perspective	8	State the perspective(s) adopted by the study and why	P5
Time horizon	9	State the time horizon for the study and why appropriate.	P5
Discount rate	10	Report the discount rate(s) and reason chosen.	P8
Selection of outcomes	11	Describe what outcomes were used as the measure(s) of benefit(s) and harm(s).	P8
Measurement of outcomes	12	Describe how outcomes used to capture benefit(s) and harm(s) were measured.	P8
Valuation of outcomes	13	Describe the population and methods used to measure and	P5,10
Measurement and valuation of resources and costs	14	Describe how costs were valued.	P7
Currency, price date, and conversion	15	Report the dates of the estimated resource quantities and unit costs, plus the currency and year of conversion.	P7
Rationale and description of model	16	If modelling is used, describe in detail and why used. Report if the model is publicly available and where it can be accessed.	P5
Analytics and assumptions	17	Describe any methods for analysing or statistically transforming data, any extrapolation methods, and approaches for validating any model used.	P5
Characterizing heterogeneity	18	Describe any methods used for estimating how the results of the study vary for subgroups.	P6-9
Characterizing distributional effects	19	Describe how impacts are distributed across different individuals or adjustments made to reflect priority populations.	P6-9
Characterizing uncertainty	20	Describe methods to characterise any sources of uncertainty in the analysis.	P9
Approach to engagement with patients and others affected by the study	21	Describe any approaches to engage patients or service recipients, the general public, communities, or stakeholders (such as clinicians or payers) in the design of the study.	Not Applicable

**Results**			
Study parameters	22	Report all analytic inputs (such as values, ranges, references) including uncertainty or distributional	P6-9, Table 1
Summary of main results	23	Report the mean values for the main categories of costs and outcomes of interest and summarise them in the most appropriate overall measure.	P11-13
Effect of uncertainty	24	Describe how uncertainty about analytic judgments, inputs, or projections affect findings. Report the effect of choice of discount rate and time horizon, if applicable.	P11-13
Effect of engagement with patients and others affected by the study	25	Report on any difference patient/service recipient, general public, community, or stakeholder involvement made to the approach or findings of the study	Not Applicable
**Discussion**			
Study findings, limitations, generalizability, and current knowledge	26	Report key findings, limitations, ethical or equity considerations not captured, and how these could affect patients, policy, or practice.	P14-17
**Other relevant information**			
**Source of funding**	27	Describe how the study was funded and any role of the funder in the identification, design, conduct, and reporting	P19
Conflicts of interest	28	Report authors conflicts of interest according to journal or International Committee of Medical Journal Editors [illegible]	P19
