# Supplementary material for: Effectiveness and cost-effectiveness analysis of 11 treatment paths, seven first-line and three second-line treatments for Chinese patients with advanced wild-type squamous non-small cell lung cancer: A sequential model
Source: Front Public Health. 2023 Feb 24;11:1051484. doi: 10.3389/fpubh.2023.1051484 (PMC9999022; doi:10.3389/fpubh.2023.1051484)
Supplement: Supplementary file 3 [file Data_Sheet_3.DOCX]

**Supplement 2**

Contents

[1. eMethod 1: Modeling Process for Decision Analysis Model 1](#_Toc112454268)

[2. eMethod 2: Eligibility Criteria 3](#_Toc112454269)

[3. eMethod 3: Search Strategies 3](#_Toc112454270)

[4. Original PFS and OS curves for each regimen (eFigure 1) 4](#_Toc112454271)

[5. Log Cumulative Hazards and Schoenfeld Residual Test Plots (eFigure 2) 6](#_Toc112454272)

[6. eMethod 4: Literature Search and Study Characteristics (eFigure 3) 10](#_Toc112454273)

[7. Network Plot (eFigure 4) 11](#_Toc112454274)

[8. Risk of Bias Assessment (eFigure 5) 12](#_Toc112454275)

[9. Survival Curves Fitted by Fractional Polynomial Models for First-line NMA (eFigure 6) 13](#_Toc112454276)

[10. Survival Curves Fitted by Fractional Polynomial Models for Second-line NMAs (eFigure 7) 16](#_Toc112454277)

[11. Kaplan-Meier Survival Curves and Parametric Reconstruction Survival Curves of Each Treatment (eFigure 8) 21](#_Toc112454278)

[12. Deviance Information Criterion (DIC) for Network Meta-analysis (eTable 1) 22](#_Toc112454279)

[13. Akaike Information Criterion (AIC) for the Referred PFS and OS Curves (eTable 2) 24](#_Toc112454280)

[14. eMethod 5: Selection Process for Parametric Survival Models and Model Validation 25](#_Toc112454281)

[15. eMethod 6: Details for Patient Assistance Programs 28](#_Toc112454282)

# eMethod 1: Modeling Process for Decision Analysis Model

We developed a sequential micro-simulation model to estimate the survival and cost outcomes. Steven et al.^[1]^ have explored the cost-effectiveness and potential budgetary consequences of durvalumab consolidation therapy vs no consolidation therapy after chemoradiotherapy in stage III NSCLC in the context of the US health care system, we adopted a micro-simulation model similar to theirs. The cumulative progression-free survival probability at different time points in the PFS stage was derived from the PFS curves of the first-line treatment. A sequential model was used to bridge the state transition of patients from first-line treatments to post first-line treatments under the precise treatment pathways. That is, we first divided the patients into three types: stayed in the PFS, entered the second-line treatment (first-stage PD stage) or death. Only age-, gender- specific natural mortality was considered for the transition probability from the PFS stage directly to the death stage, natural mortality was extracted from China's 6th National Census^[2]^, information of age and gender were from the randomized controlled trials of corresponding intervention options. Once the living patient was out of the first-line treatment stage, the patient would be in the first cycle of second-line treatment. Using the idea of partitioned survival model, we obtained the cumulative progression-free survival rate and mortality rate at any time point.

After the cumulative transition probability was obtained, the cyclical transition probability was needed in the micro-simulation model. The competing risk model was adopted to deal with the cyclical transition probability of disease progression and death in our micro-simulation model for first-line treatment and second-line treatment. The cyclical transition probability is calculated as follows:

**First-line period (PFS state in the model)**

Cyclical transition probability of PFS to PFS at cycle T:

$$P_{\mathrm{aT}}=S_{\mathrm{aT}}/S_{aT-1}$$

Where, $S_{\mathrm{aT}}$ is the cumulative progression-free survival probability derived from the first-line PFS curves.

Cyclical transition probability of PFS to first-stage PD at cycle T:

$$P_{\mathrm{bT}}=1-S_{\mathrm{aT}}/S_{aT-1}$$

Cyclical transition probability of PFS to death at cycle T:

$$P_{\mathrm{cT}}=\mathrm{ND}_{T}/\mathrm{ND}_{T-1}$$

Where, $\mathrm{ND}_{T}$ equals to the age-, gender- specific natural mortality.

The cyclical natural mortality was calculated from annual natural mortality, we assumed the natural mortality changes exponentially, details of calculation are as follows:

$$N_{T}=1-EXP(-Y_{T}*(21/365.25))$$

Where, $Y_{T}$ equals to annual natural mortality at year T.

**Post first-line period (first-stage PD and end-stage PD)**

Cyclical transition probability of first-stage PD to first-stage PD at cycle T:

$$P_{\mathrm{cT}}=S_{\mathrm{bT}}/S_{bT-1}$$

Where, $S_{\mathrm{bT}}$ is the cumulative progression-free survival probability derived from the second-line PFS curves.

Cyclical transition probability of first-stage PD to second-stage PD at cycle T:

$$P_{\mathrm{dT}}=1-S_{\mathrm{bT}}/S_{bT-1}$$

Cyclical transition probability of first- or second-stage PD to death at cycle T:

$$P_{\mathrm{eT}}=1-S_{\mathrm{cT}}/S_{cT-1}$$

Where, $S_{\mathrm{cT}}$ is the cumulative overall survival probability derived from the second-line OS curves.

Besides, considering that most of the clinical trials in this study were followed for less than 2 years, the short follow-up period may not be sufficient to capture the risk of death due to natural mortality, for example, no natural death case appeared in RCTs. Thus, age-, gender- specific natural mortality was used to adjust the OS or PFS curves after the time point at which the terminal phase of the original OS or PFS curves appeared to plateau.

Considering that the model in this study is a sequential model, and the cyclical transition probability changes with time. Therefore, partition survival models or markov models with traditional memoryless assumptions do not apply. The modeling of sequential model should be based on the time-reset, and at the same time, model needs to satisfy the transition probability that is variable over time. Therefore, after the cyclical transition probability transition probability was obtained, a mirco-simulation model was built to overcome the above limitations. The modeling process for mirco-simulation model is presented as follows:

The probability of each event is determined through a stochastic/random walk process (first-order uncertainty) in which the probability is compared with a value drawn from a uniform probability distribution X~U[0, 1] to establish if the event has occurred in the current cycle^[3]^.

According to **Figure 1**, we assumed that only when the patient was out of PFS state, the other events may occur. For the risks of PFS stage transferred to first-stage PD or death, the competitive risk model was used, and death event was assumed to be more prioritized. Likewise, patients receiving second-line therapy (first-PD stage) would also face competing risks of disease progression only and death, we assumed death risk to be more prioritized.

Once the living patients was out of PFS state, he or she would enter the first-cycle of second-line treatment. “COUNTIF” function was used to determine the cumulative survival periods of the patient at the corresponding state, “Lookup” function was used to determine the corresponding cyclical transition probability for certain cycle at each state in Mircosoft Excel.

# eMethod 2: Eligibility Criteria

The study populations were ≥18 years of age with stage IIIB to IV sq-NSCLC, PD-L1 expression level was unlimited, without known EGFR sensitising mutations, or ALK, ROS1, or RET fusions, and had not previously received systemic therapy. The interventions were PD-(L)1 inhibitors that were already in or about to enter the market in China as first-line therapeutic regimens of sq-NSCLC, whose phase III clinical trials were completed and data were available. The intervention group received PD-(L)1 inhibitor combined with chemotherapy, and chemotherapy was limited to pemetrexed plus platinum and paclitaxel/gemcitabine plus platinum, which were approved in China for first-line treatment of advanced sq-NSCLC. The control group received chemotherapy only.

Overall survival (OS), progression-free survival (PFS), and serious adverse events (SAEs) were the outcome indicators. The corresponding hazard ratio (HR) or odd ratio (OR) and related 95% confidence interval (CI) should also be reported. If a study did not report at least the HR of PFS or OS, it would be excluded.

The studies included were limited to phase III randomized controlled clinical trials. In cases of different published studies or conference abstracts of the same clinical trial, we selected the latest and the most comprehensive version. For the interactions with multiple clinical trials, we selected the trials meeting the limited standards and having similar experimental designs. Clinical trials that did not focus on the Chinese population will be excluded.

# eMethod 3: Search Strategies

As of May 2022, we systematically searched the PubMed (https://pubmed.ncbi.nlm.nih.gov), Embase (https://www.embase.com), and ClinicalTrials.Gov (https://clinicaltrials.gov) to retrieve clinical trials and published studies of associated drugs. We also searched abstracts in European Society for Medical Oncology, American Society of Clinical Oncology, and World Conference on Lung Cancer. There was no limit for the study period, and the language was limited to Chinese or English.

（1）PubMed

| No | Items |
| --- | --- |
| #1 | PD-1[Title/Abstract] OR PD-L1[Title/Abstract] OR Immunotherapy [Title/Abstract] |
| #2 | sq[Title/Abstract] OR Squamous[Title/Abstract] |
| #3 | NSCLC[Title/Abstract] OR Non-small cell lung cancer [Title/Abstract] |
| #4 | #1 AND #2 AND #3 |
| #5 | Animals [Title] OR Human [Title] |
| #6 | #4 NOT #5 |
| #7 | Clinical Trial [Publication Type] OR Randomized Controlled Trial [Publication Type] |
| #8 | #6 AND #7 |

（2）Embase

| #1 | 'Squamous Non-small cell lung cancer'/exp OR ('Non-small cell lung cancer') NEAR/3 (Squamous*OR cancer OR cancers* OR cancerous* OR tumor* OR tumour*)): ab,ti,kw,tn |
| --- | --- |
|  |  |
|  |  |
| #2 | Nedaplatin'/exp OR (Nedaplatin OR "Just"): ab,ti,kw,tn |
| #3 | Pembrolizumb'/exp OR (Pembrolizumb OR "Keynote407"): ab,ti,kw,tn |
| #4 | Sintilimab'/exp OR (Sintilimab OR "Orient 12"): ab,ti,kw,tn |
| #5 | Camrelizumb'/exp OR (Camrelizumb OR "CameL-sq" ):ab,ti,kw,tn |
| #6 | Tislelizumab'/exp OR (Tislelizumab OR "Rationale303" OR "Rationale307"): ab,ti,kw,tn |
| #7 | Sugemalimab '/exp OR (Tislelizumab OR "Gemstone302"): ab,ti,kw,tn |
| #8 | Nivolumab '/exp OR (Tislelizumab OR "Checkmate078"): ab,ti,kw,tn |
| #9 | #2 OR #3 OR #4 OR #5 OR #6 |
| #10 | #1 AND #11 |
| #11 | crossover procedure':de OR 'double-blind procedure':de OR 'randomized controlled trial':de OR 'single-blind procedure':de OR 'drug therapy'/lnk OR (random* OR factorial* OR crossover* OR cross NEXT/1 over* OR placebo* OR doubl* NEAR/1 blind* OR singl* NEAR/1 blind* OR assign* OR allocat* OR volunteer*):de,ab,ti |
|  |  |
|  |  |
|  |  |
|  |  |
| #12 | #10 AND #11 |
| #13 | ('animal'/exp OR 'animal experiment'/exp) NOT 'human'/exp |
| #14 | #12 NOT #13 |

（3）ClinicalTrials

https://clinicaltrials.gov/ct2/results/refine?show_xprt=Y

(Squamous Non-small-cell Lung Cancer) AND (Pembrolizumb OR Nedaplatin OR Sintilimab OR Camrelizumb OR Tislelizumab OR Chemotherapy OR Immunotherapy OR Nivolumab OR Sugemalimab ) AND (Phase 3)

# Original PFS and OS curves for each regimen (eFigure 1)

| 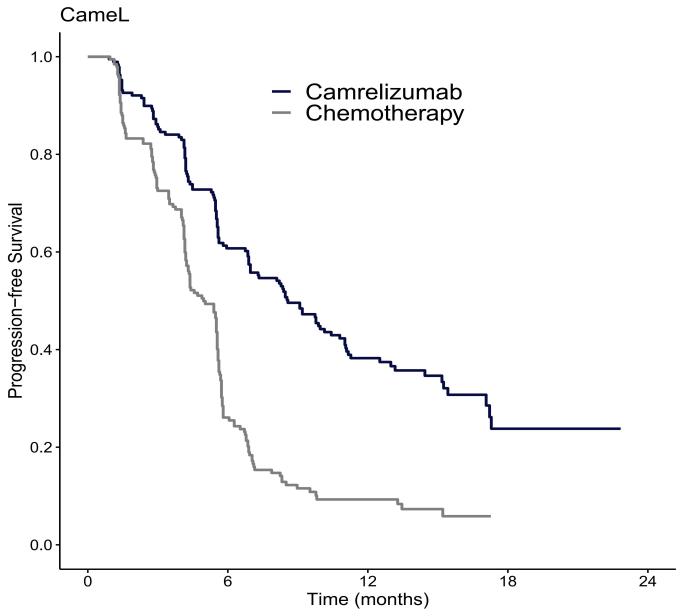 | 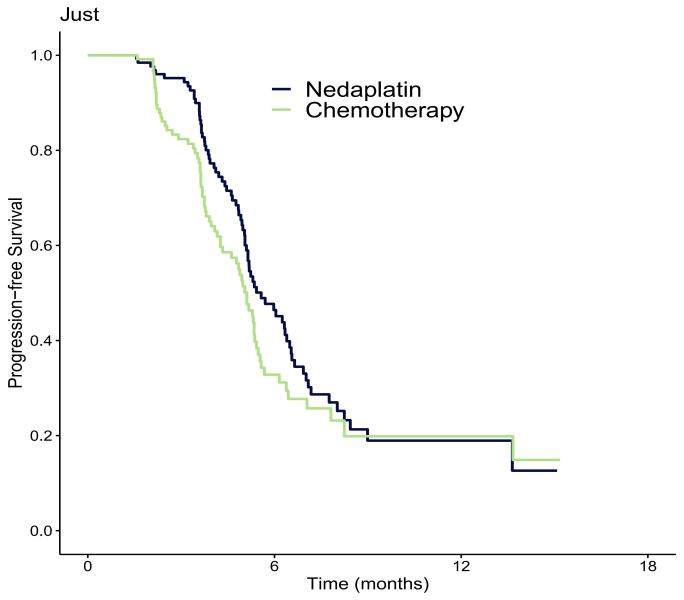 |
| --- | --- |
| 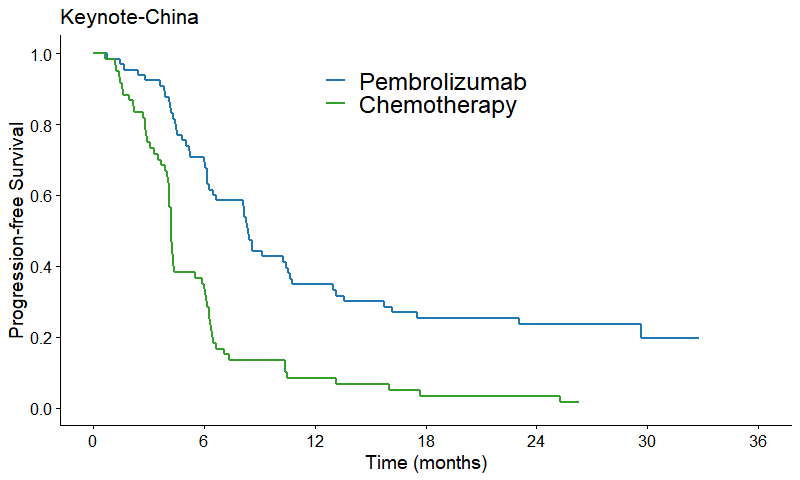 | 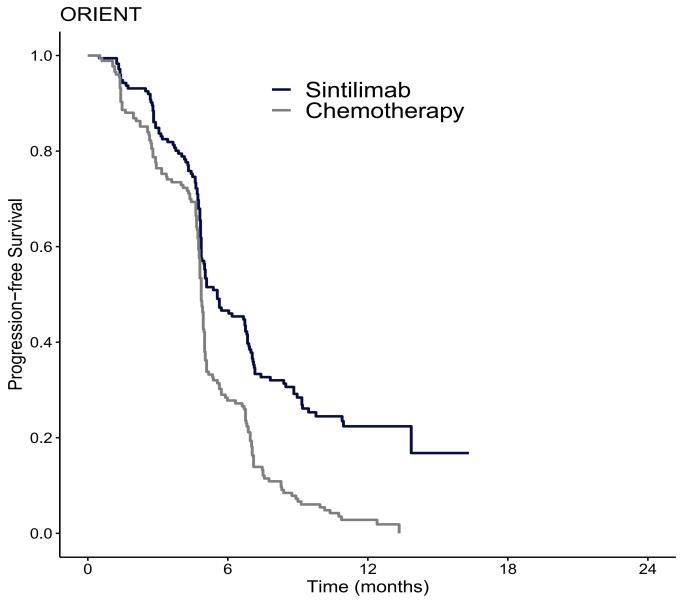 |
| 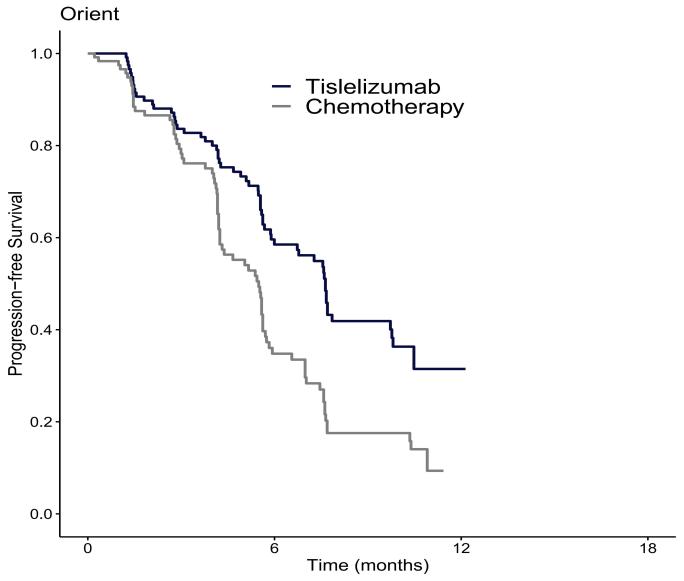 | 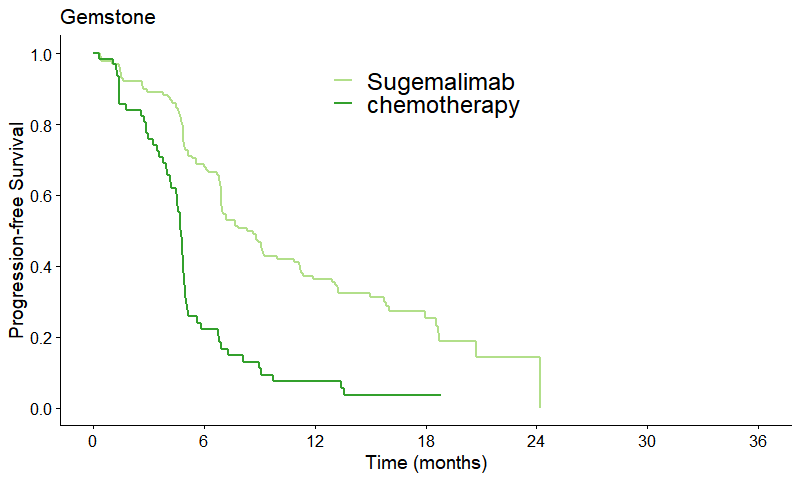 |
| 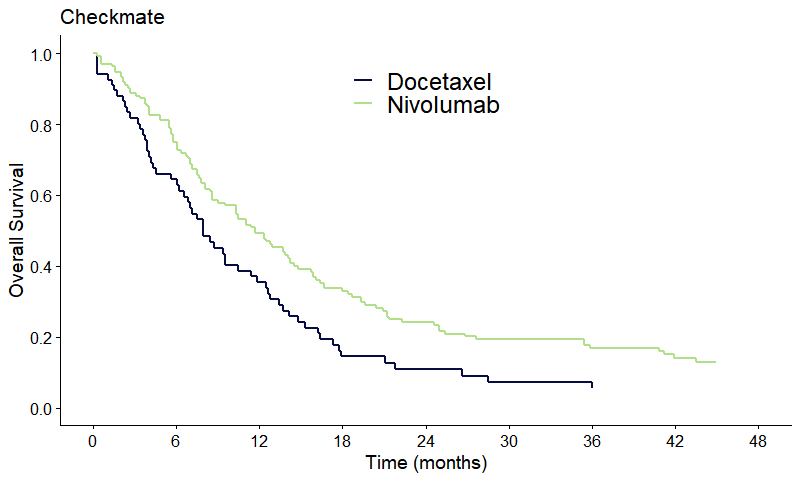 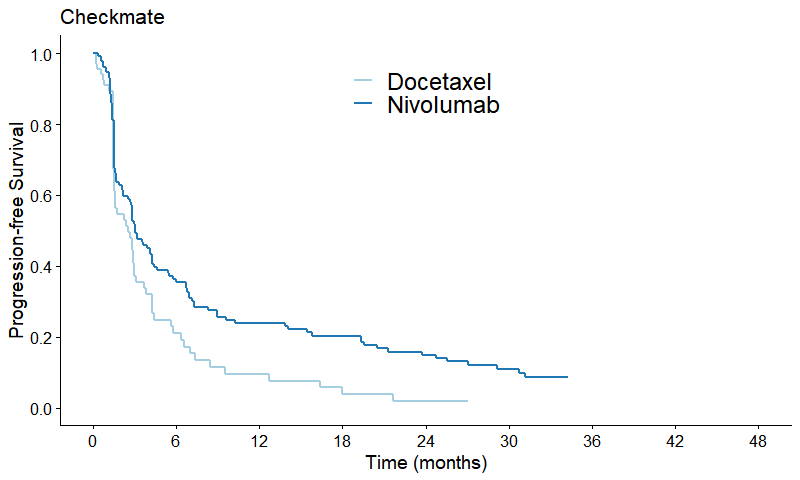 | |
| 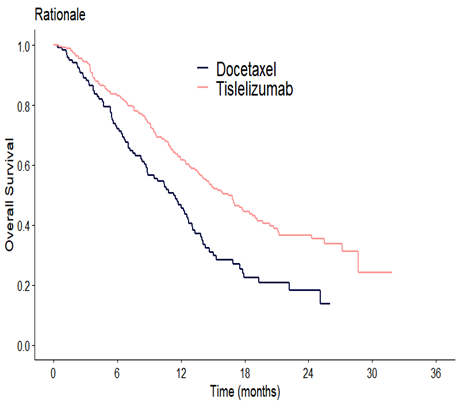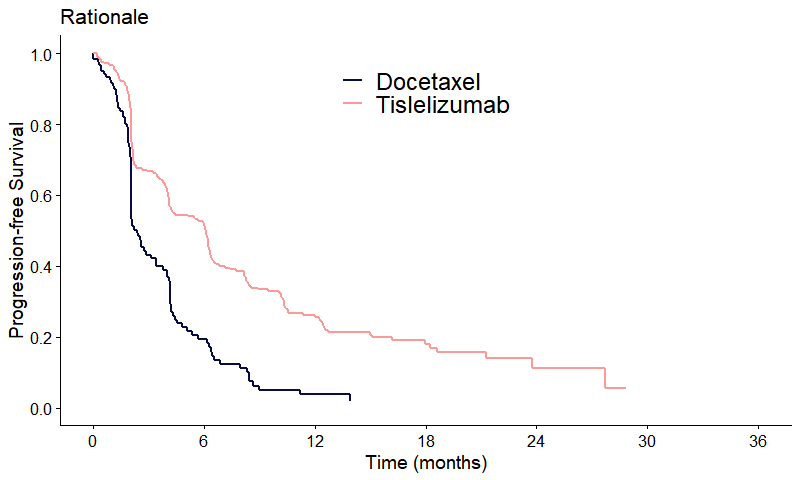 | |
| **eFigure 1 Original curves for all treatment** | |

# Log Cumulative Hazards and Schoenfeld Residual Test Plots (eFigure 2)

**First-line NMA**

| 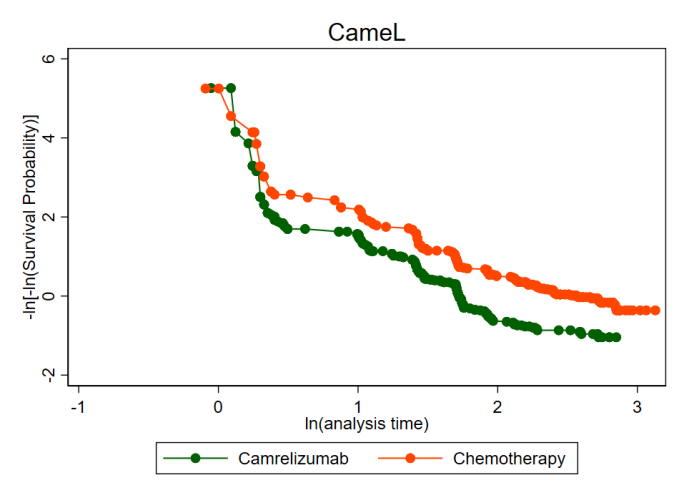 | 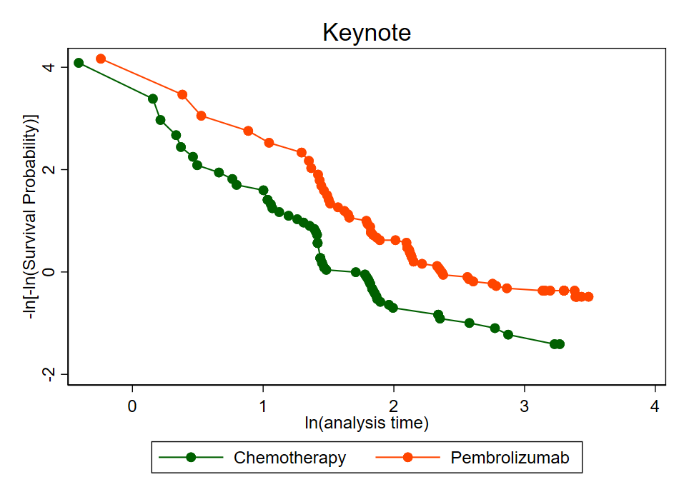 |
| --- | --- |
| 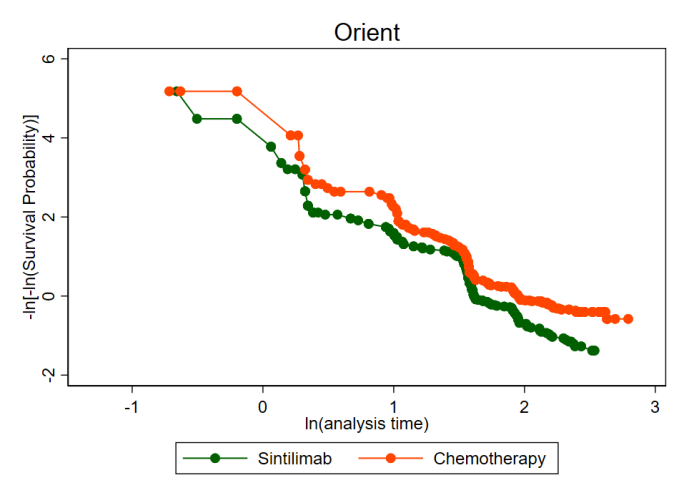 | 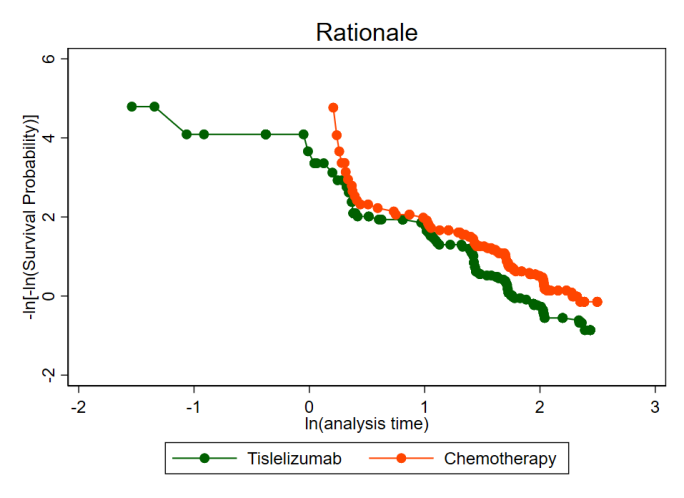 |
| 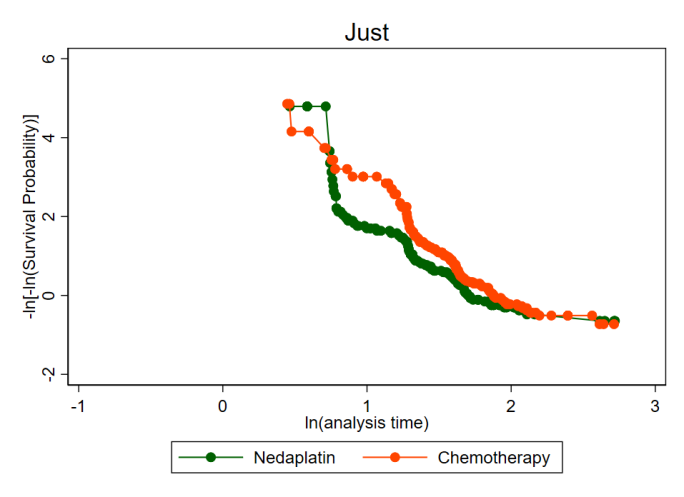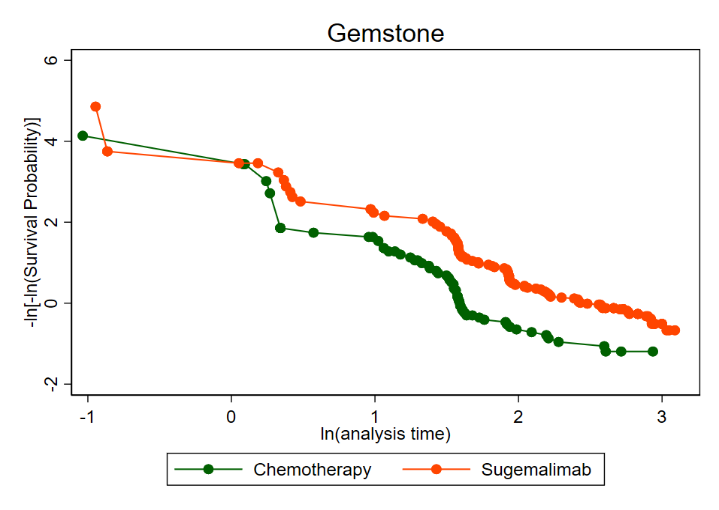  **Second-line NMA** | |
| 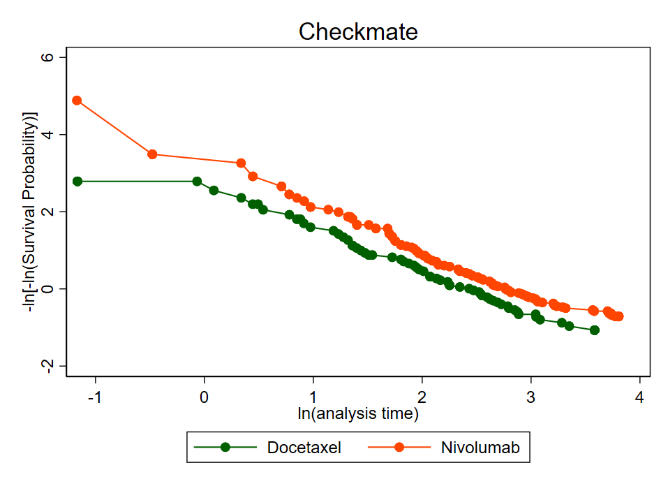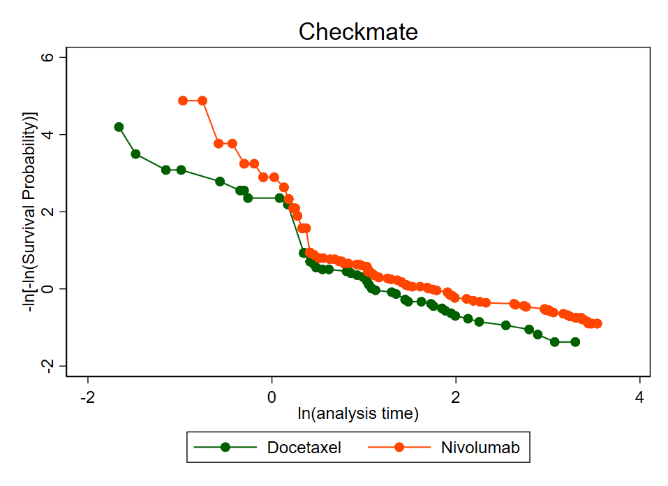  PFS  OS  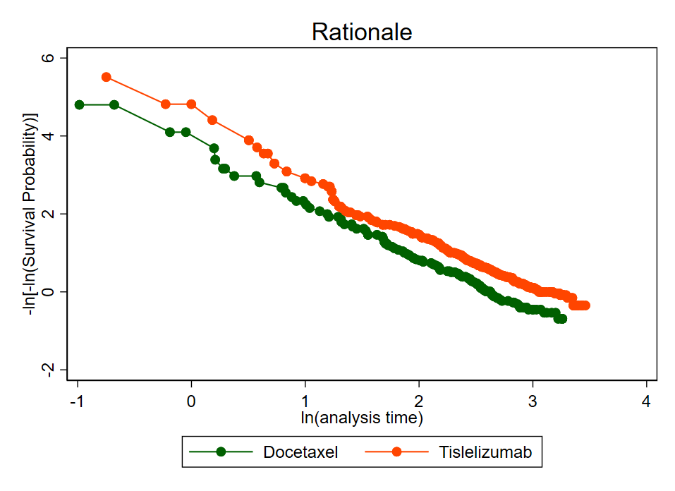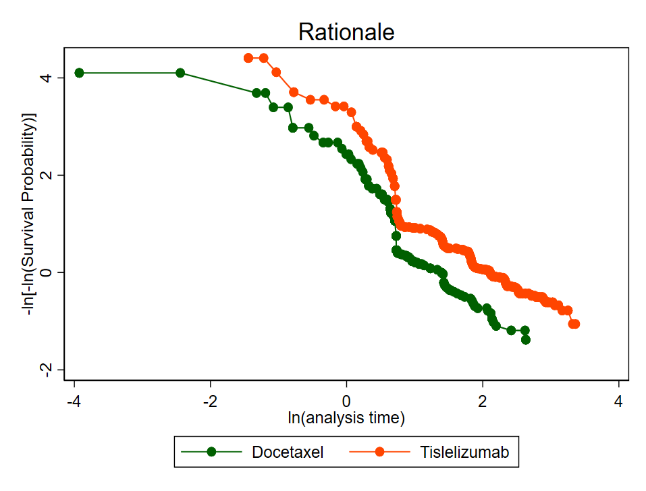  PFS  OS | |

**eFigure 2A Log Cumulative Hazards Plots**

**First-line NMA**


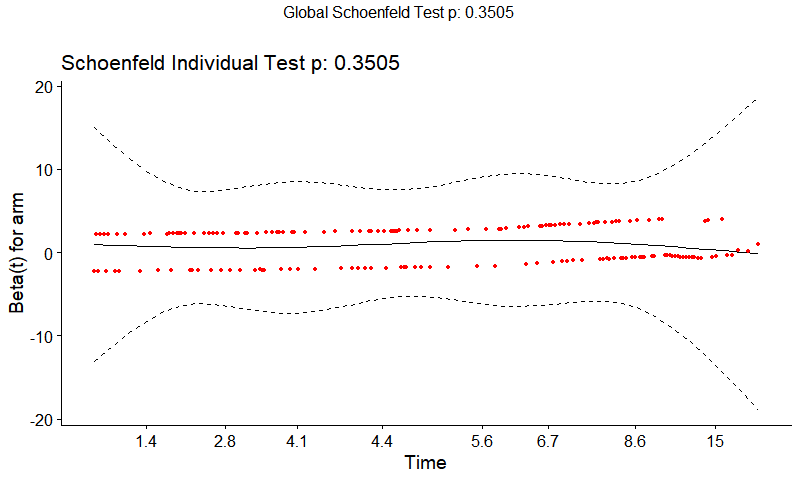


**CameL-sq**


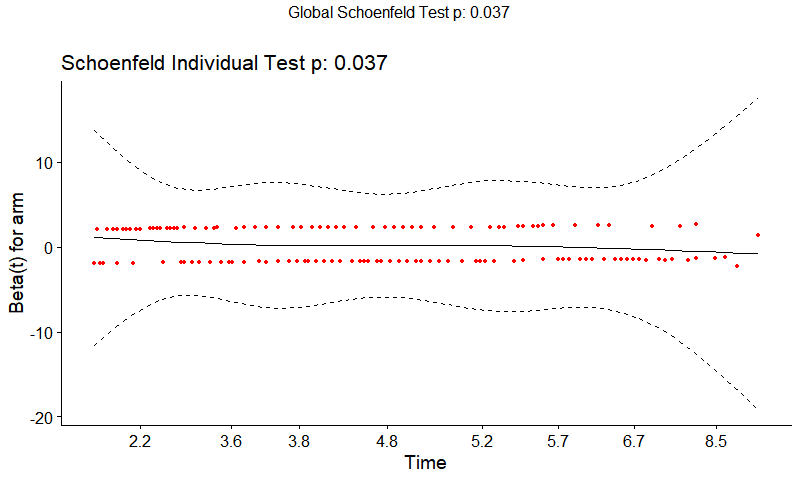


**Just**

**
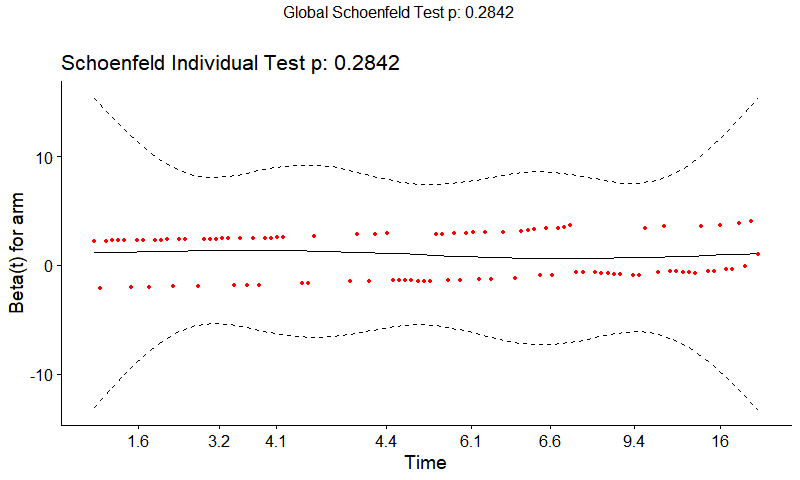
**

**Keynote-407 China**

**
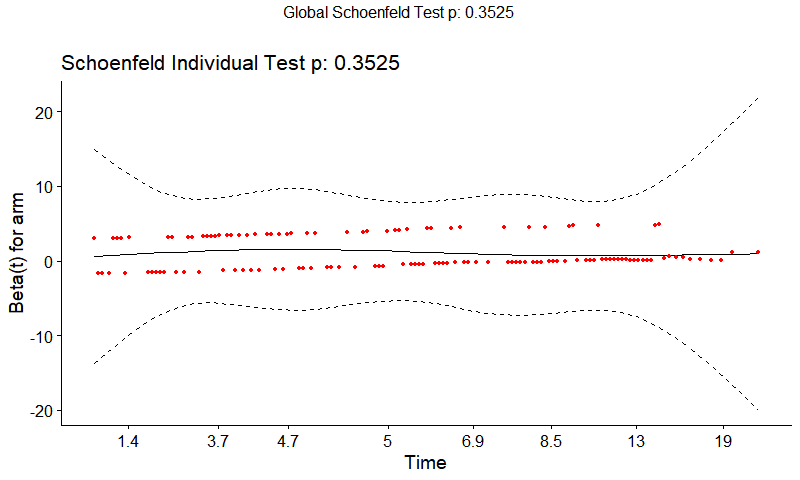
**

**Gemstone-302**

**
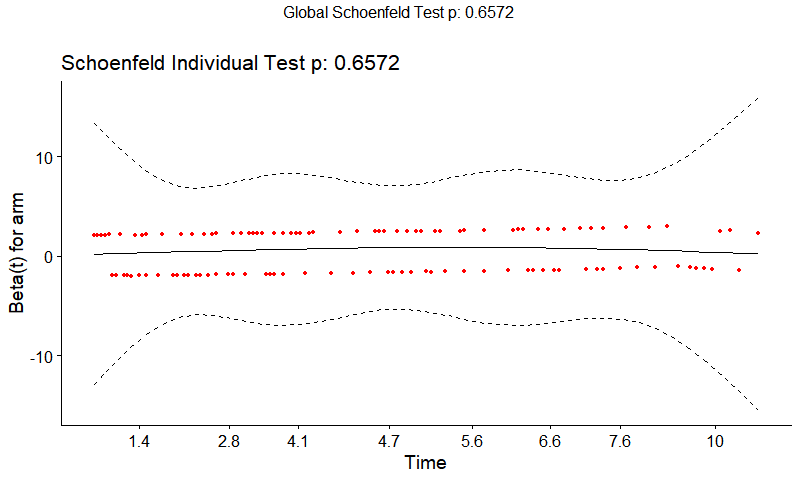
**

**Rationale-307**

**
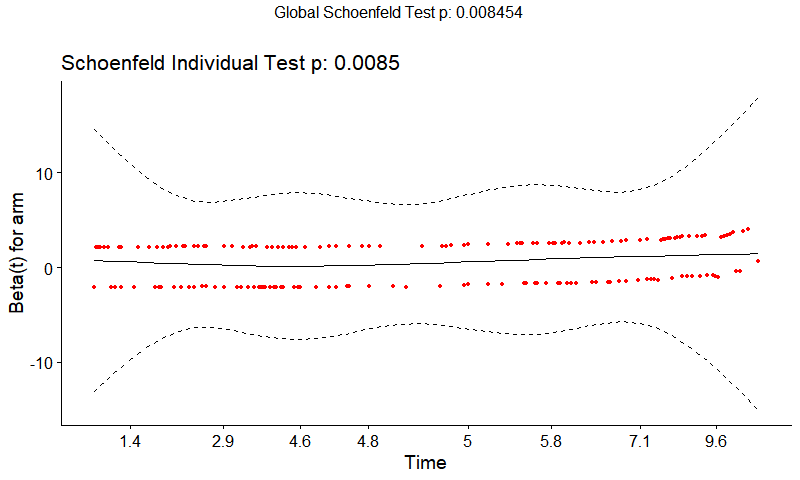
**

**Orient-12**

**Second-line NMA**

**
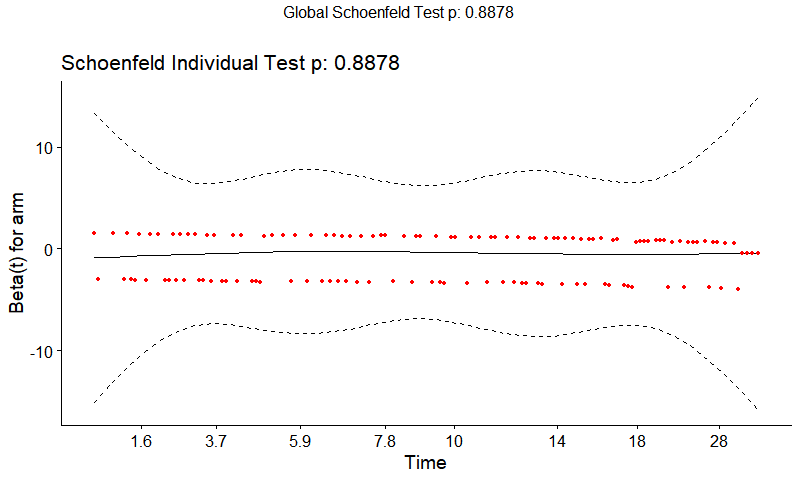
**

**Checkmate 078 (OS)**

**
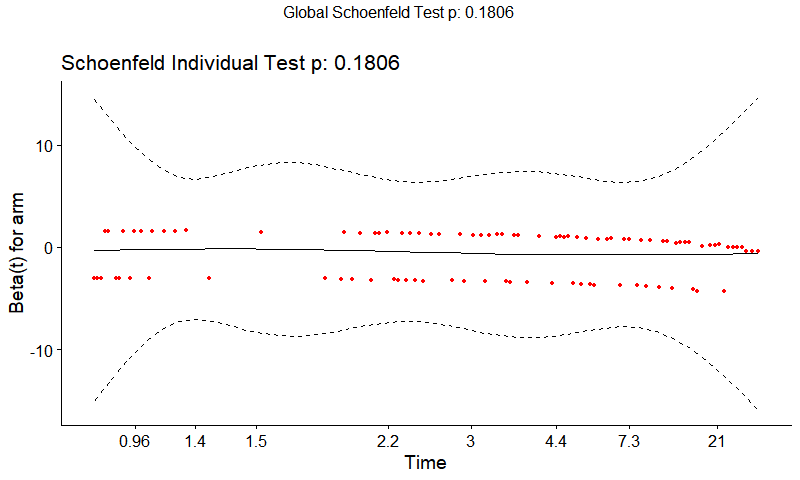
**

**Checkmate 078 (PFS)**

**
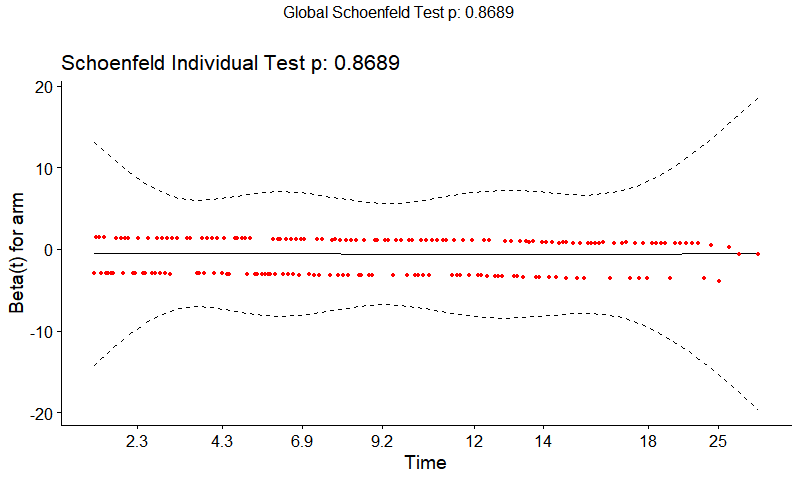
**

**Rationale-303 (OS)**

**
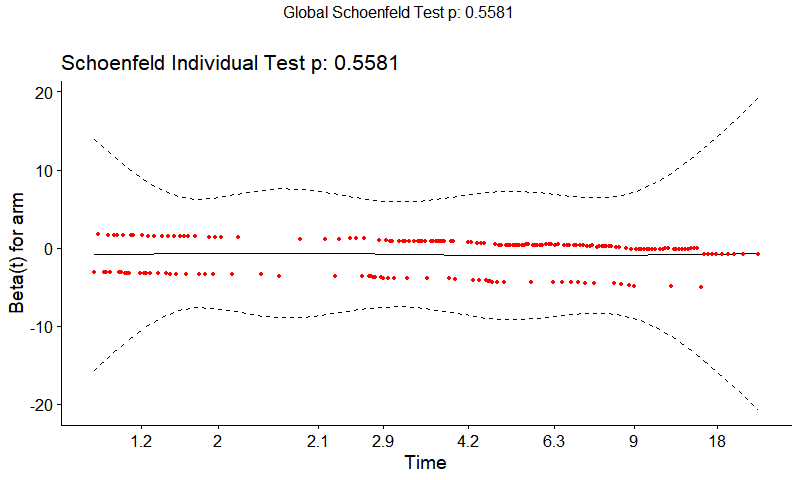
**

**Rationale-303 (PFS)**

**eFigure 2B Schoenfeld Residual Test Plots**

# eMethod 4: Literature Search and Study Characteristics (eFigure 3)

A total of 4,067 unique study records were identified, including 59 publication citations, 13 trial regulatory records and 3,997 conference abstracts. After removing duplicates following the preliminary inclusion/exclusion criteria, 9 PD-1/PD-L1 inhibitors and 22 clinical trials were retained. Full-text screening was done for these records. After comparison of the experimental designs and screening for the reporting of outcome indicators, 10 treatments with 8 clinical trials included 2,154 patients were finally included (A total of six RCTs for first-line NMA, the other two RCTs for second-line NMA). Flow chart of the literature search is shown in **eFigure 3**.


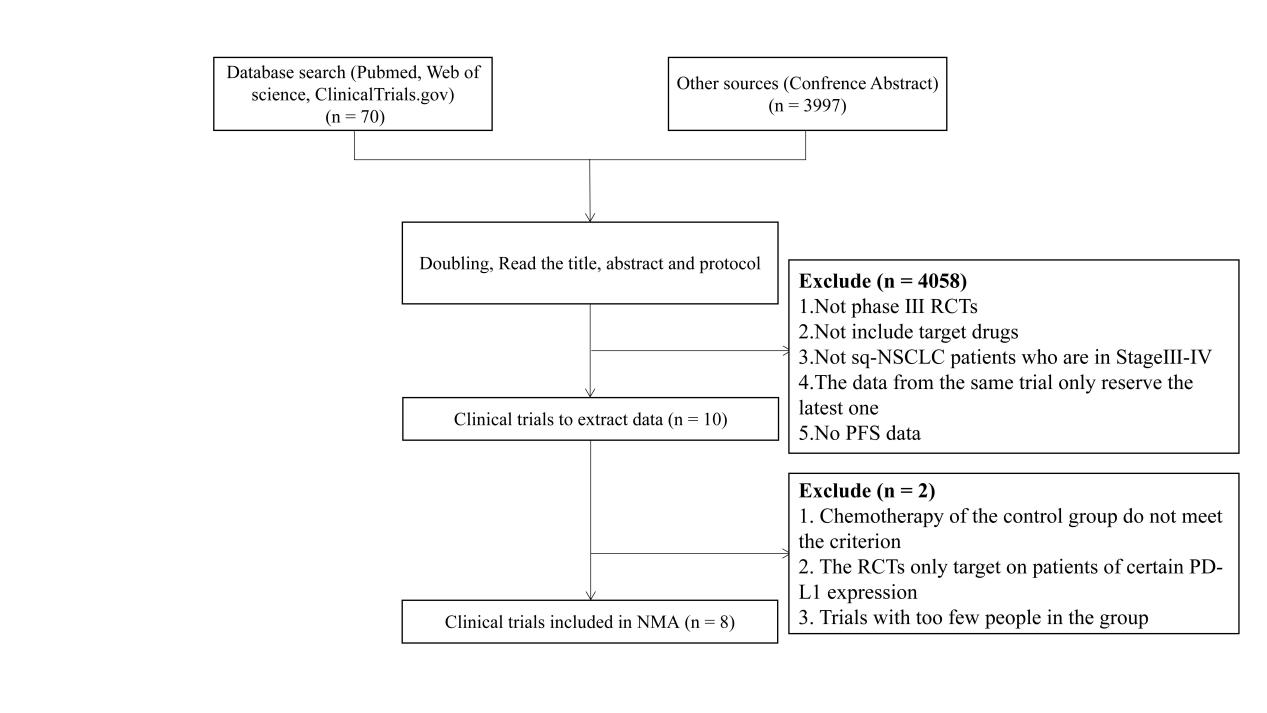


**eFigure 3 Flow Chart of Literature Search**

# Network Plot (eFigure 4)


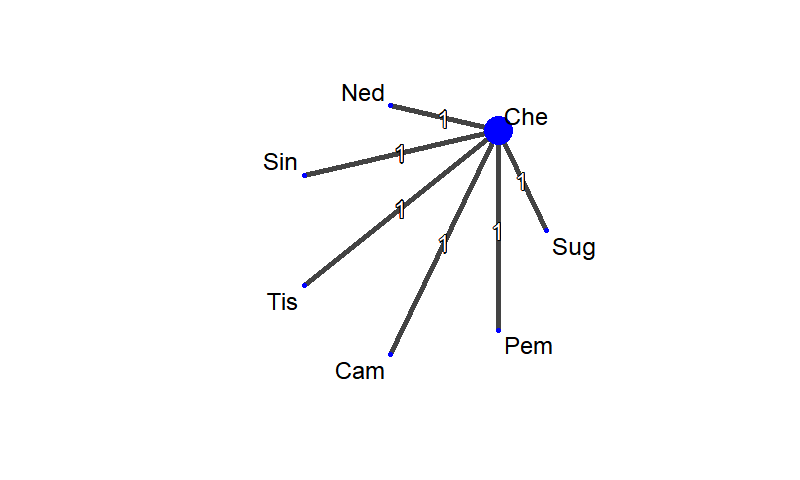


**eFigure 4A Network plot for first-line NMA**

(Che, platinum and paclitaxel-based chemotherapy; Ned, nedaplatin-based chemotherapy; Tis, tislelizumab combined with chemotherapy; Sin, sintilimab combined with chemotherapy; Pem, pembrolizumab combined with chemotherapy; Cam, camrelizumab combined with chemotherapy; Sug, sugemalimab combined with chemotherapy)

**
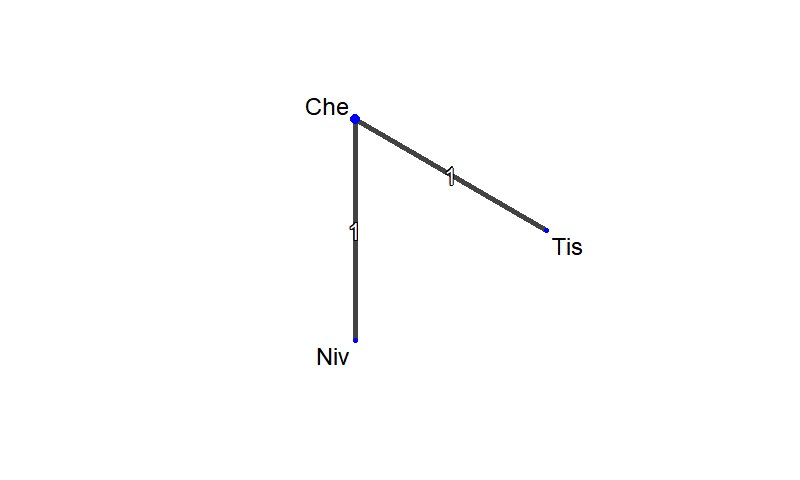
**

**eFigure 4B Network plot for second-line NMA**

(Che, Docetaxel; Tis, tislelizumab; Niv, nivolumab combined with chemotherapy)

# Risk of Bias Assessment (eFigure 5)


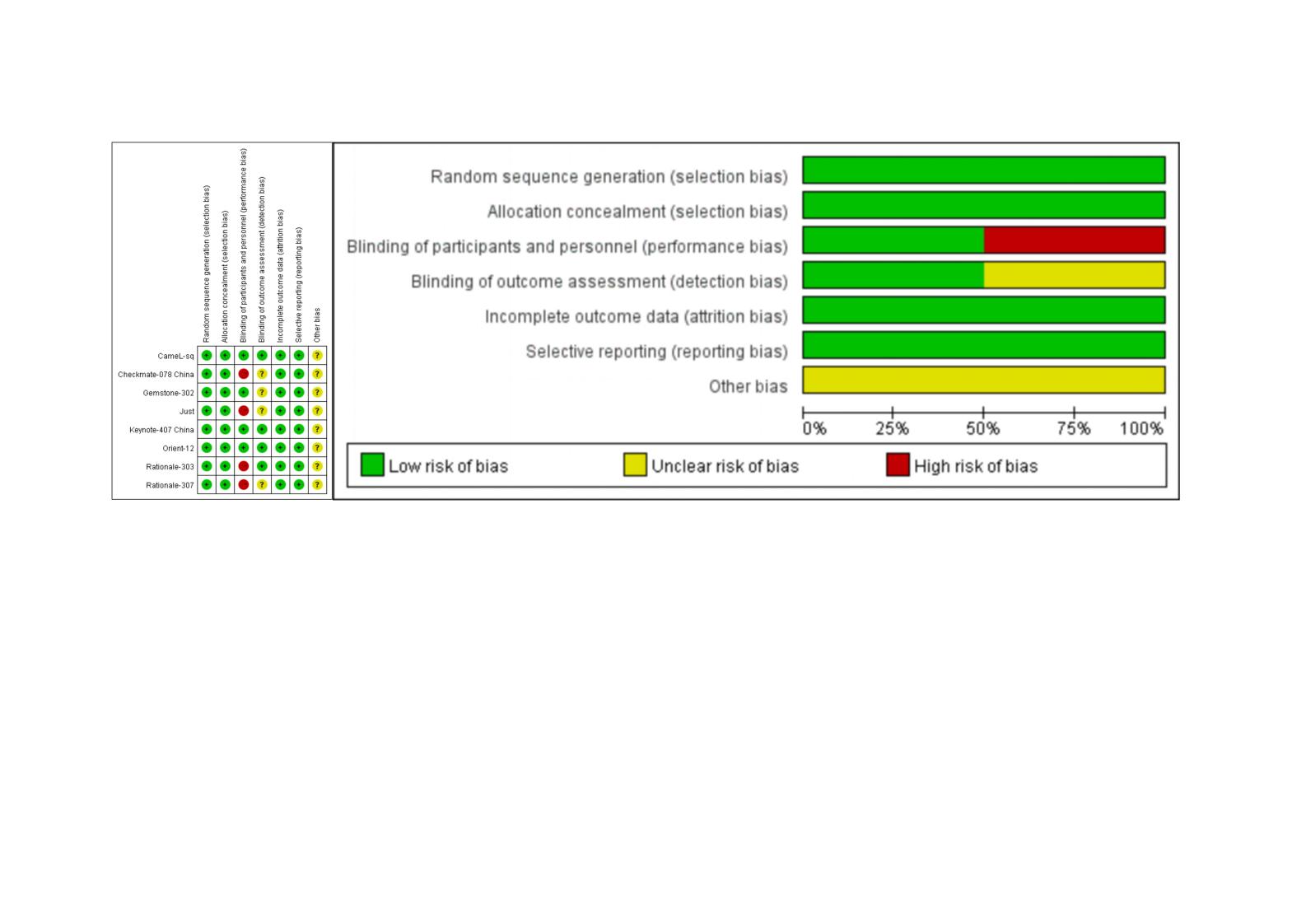


**eFigure 5 Risk of Bias** **Assessment**

# Survival Curves Fitted by Fractional Polynomial Models for First-line NMA (eFigure 6)


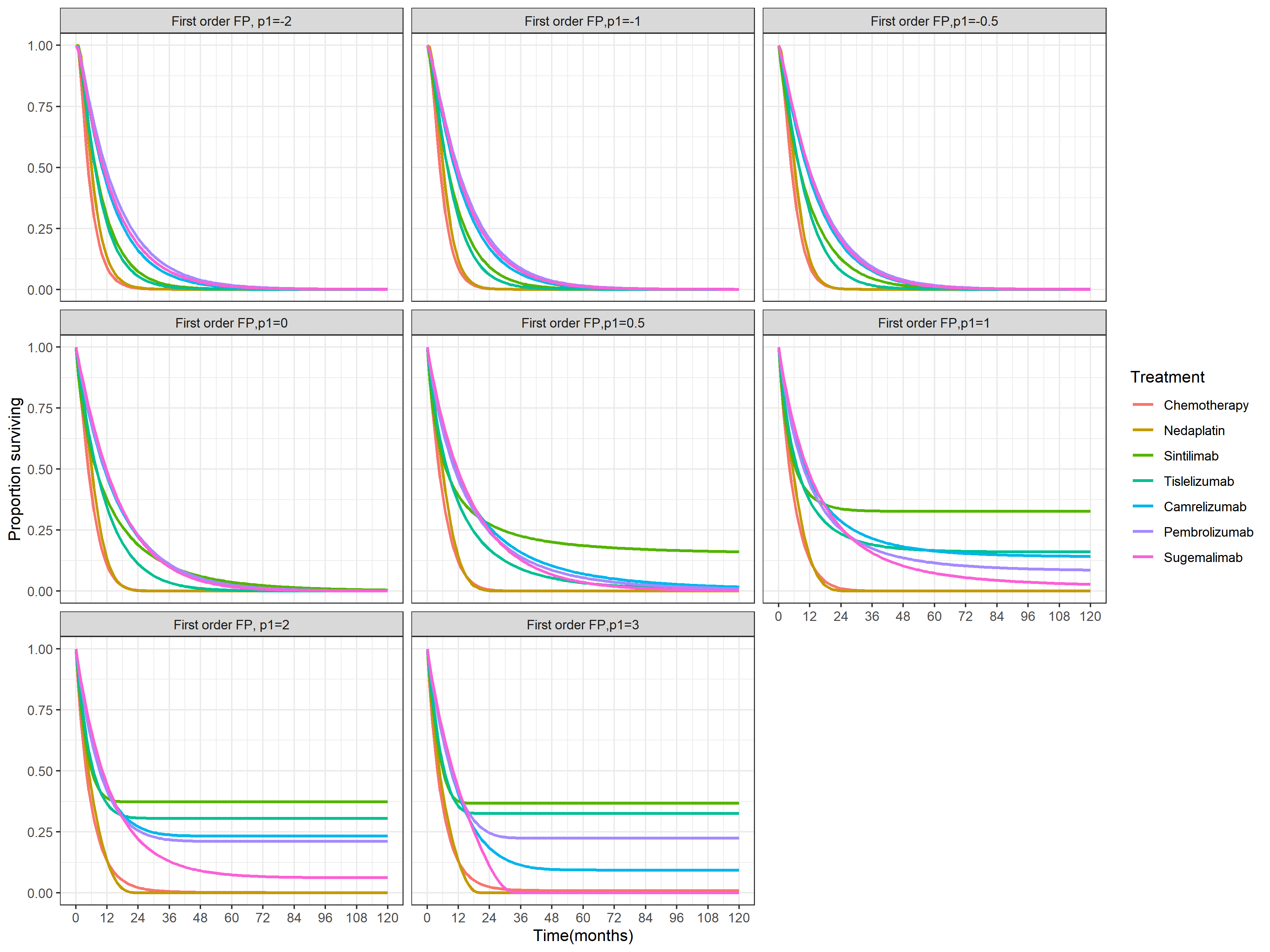


**eFigure 6A First-order** **Fractional Polynomial Models**


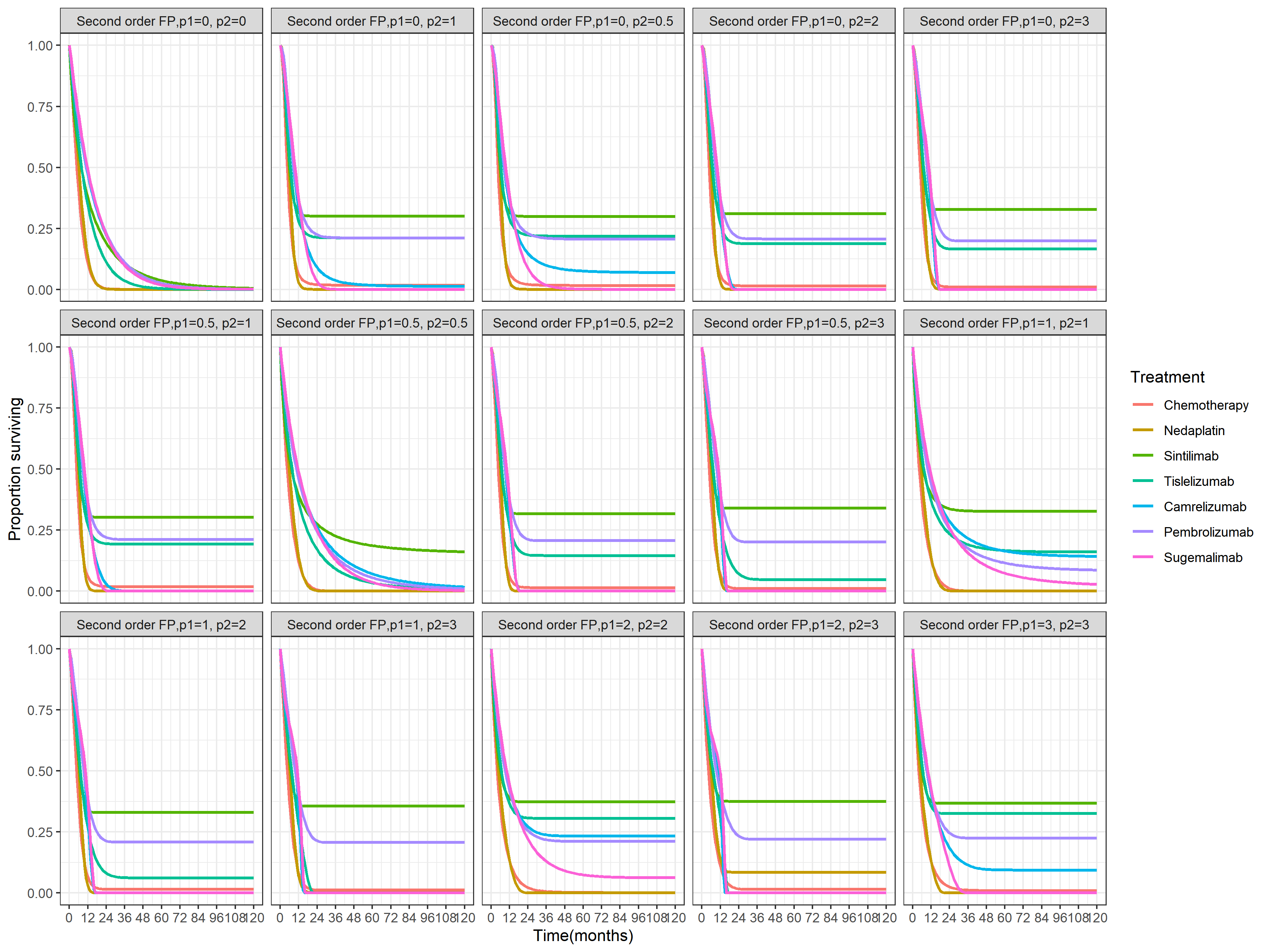

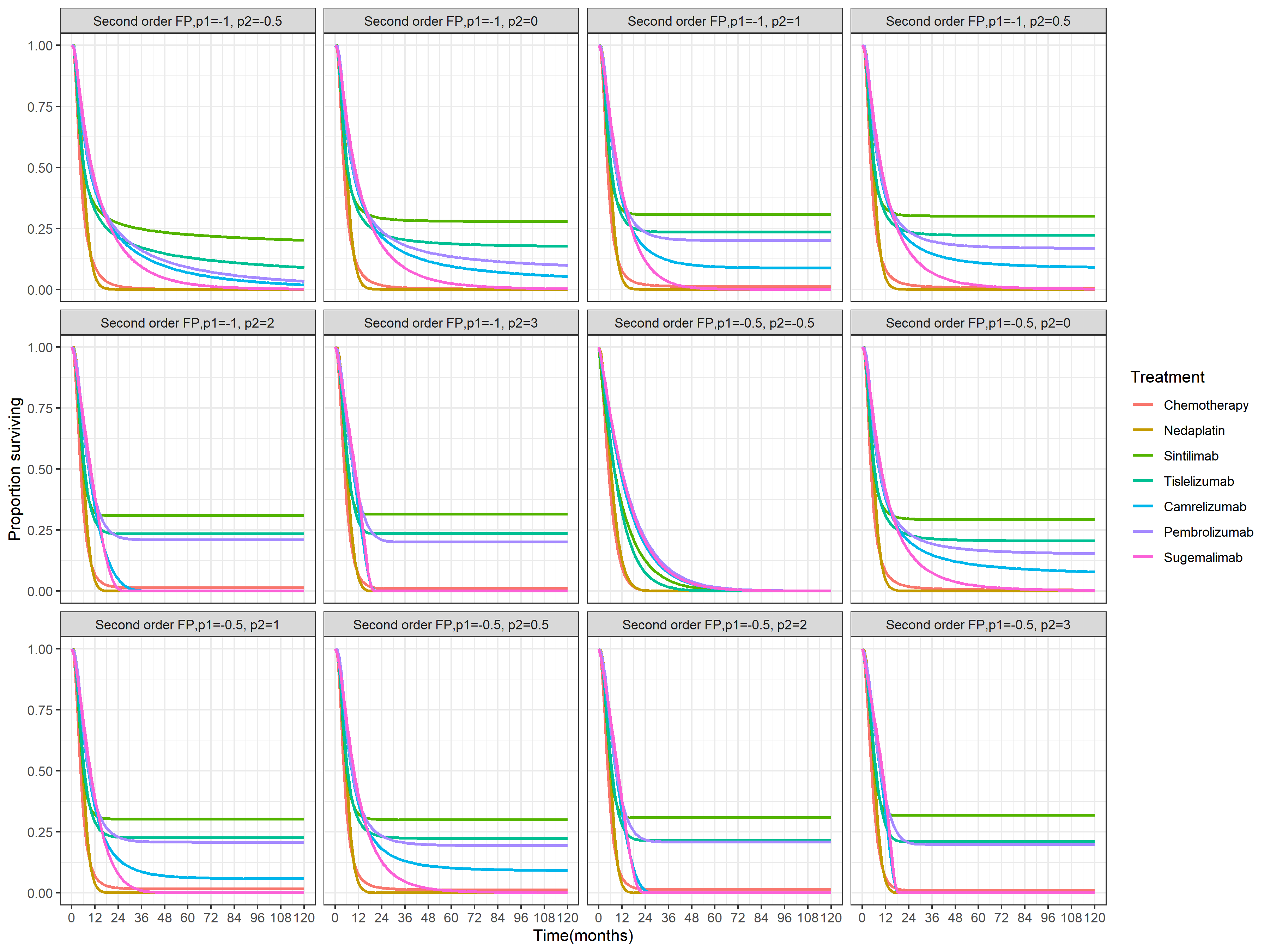


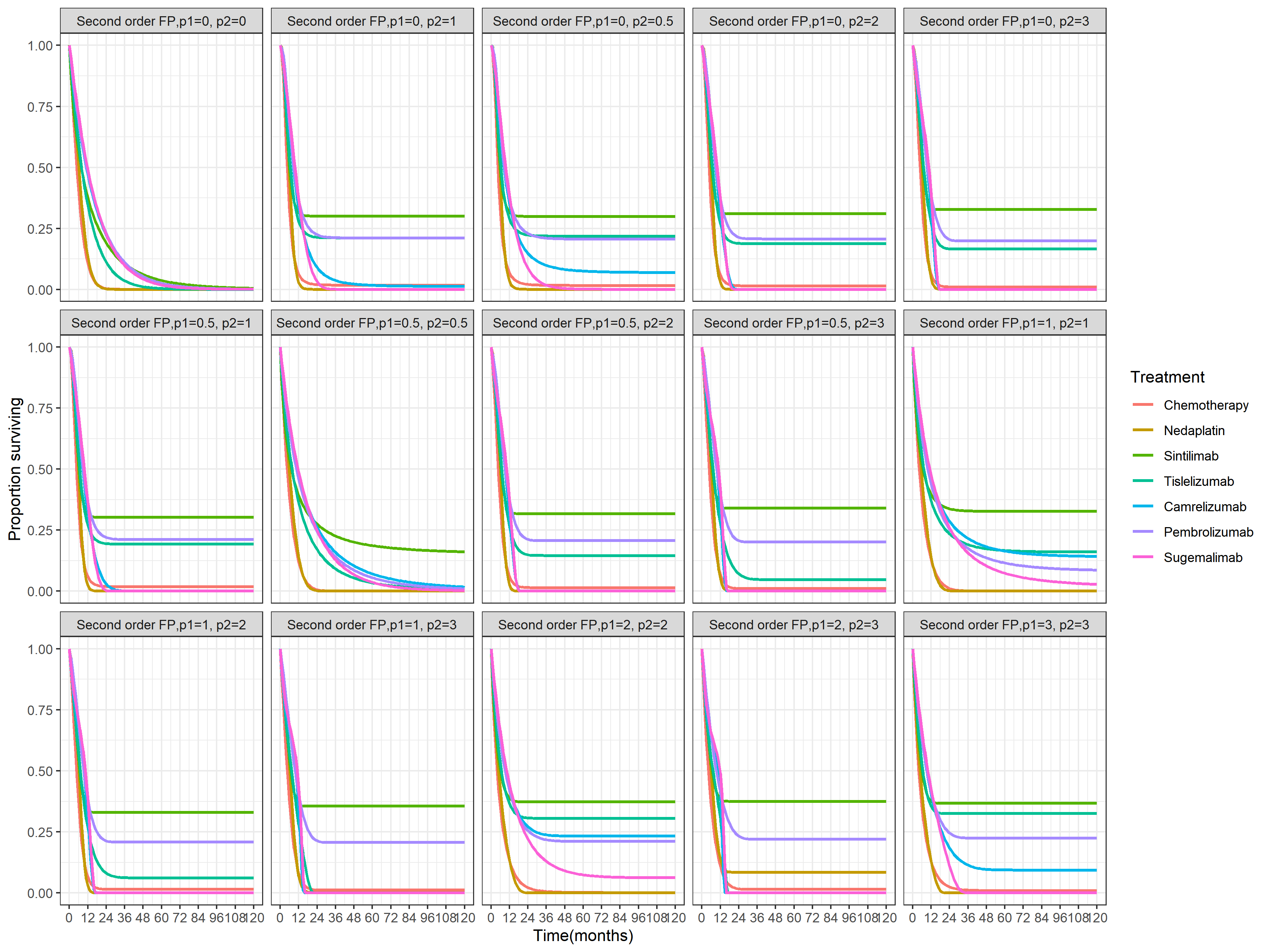


**eFigure 6B Second-order** **Fractional Polynomial Models**

# Survival Curves Fitted by Fractional Polynomial Models for Second-line NMAs (eFigure 7)


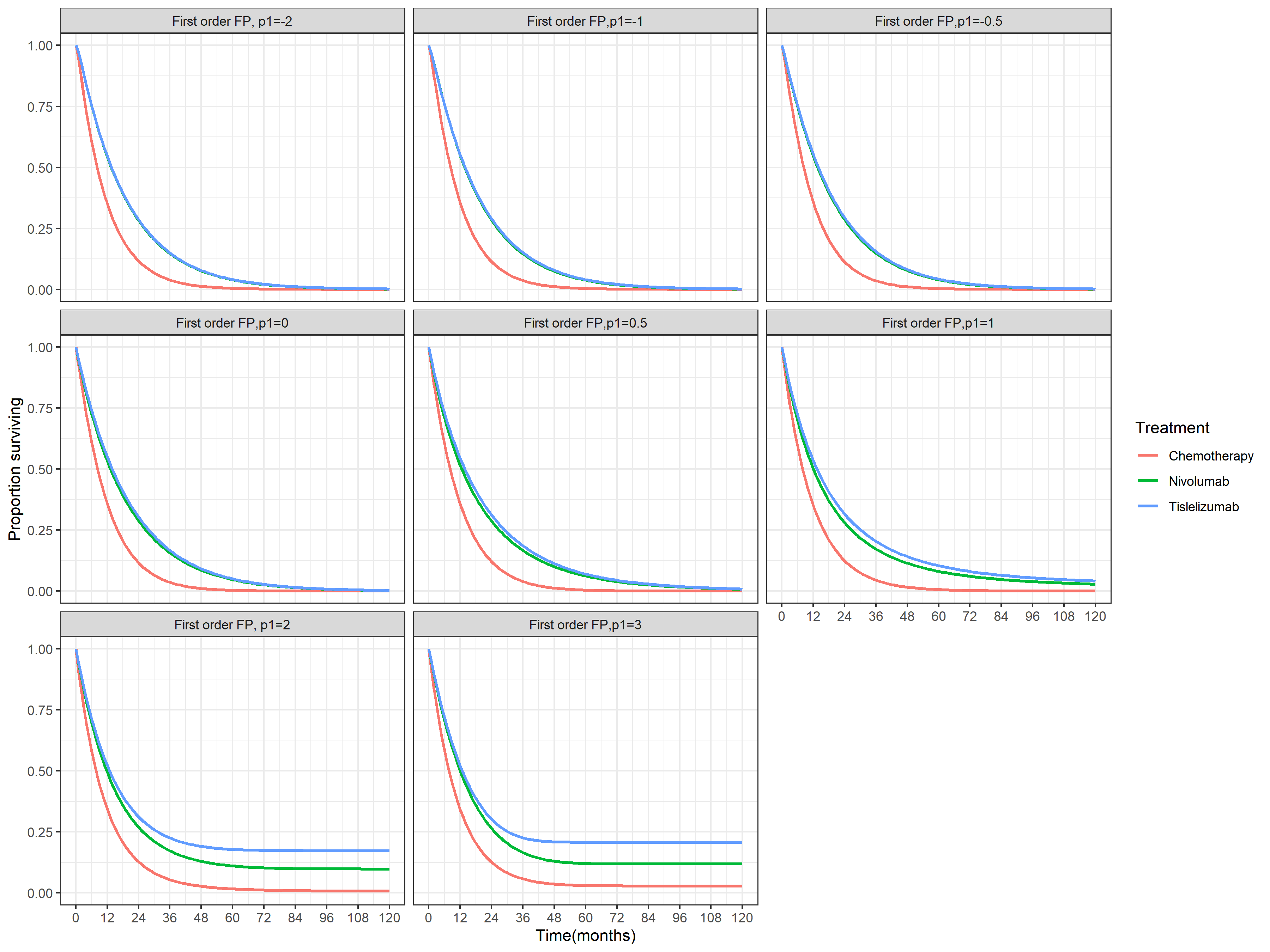


**eFigure 7A First-order Fractional Polynomial Models (OS)**

**
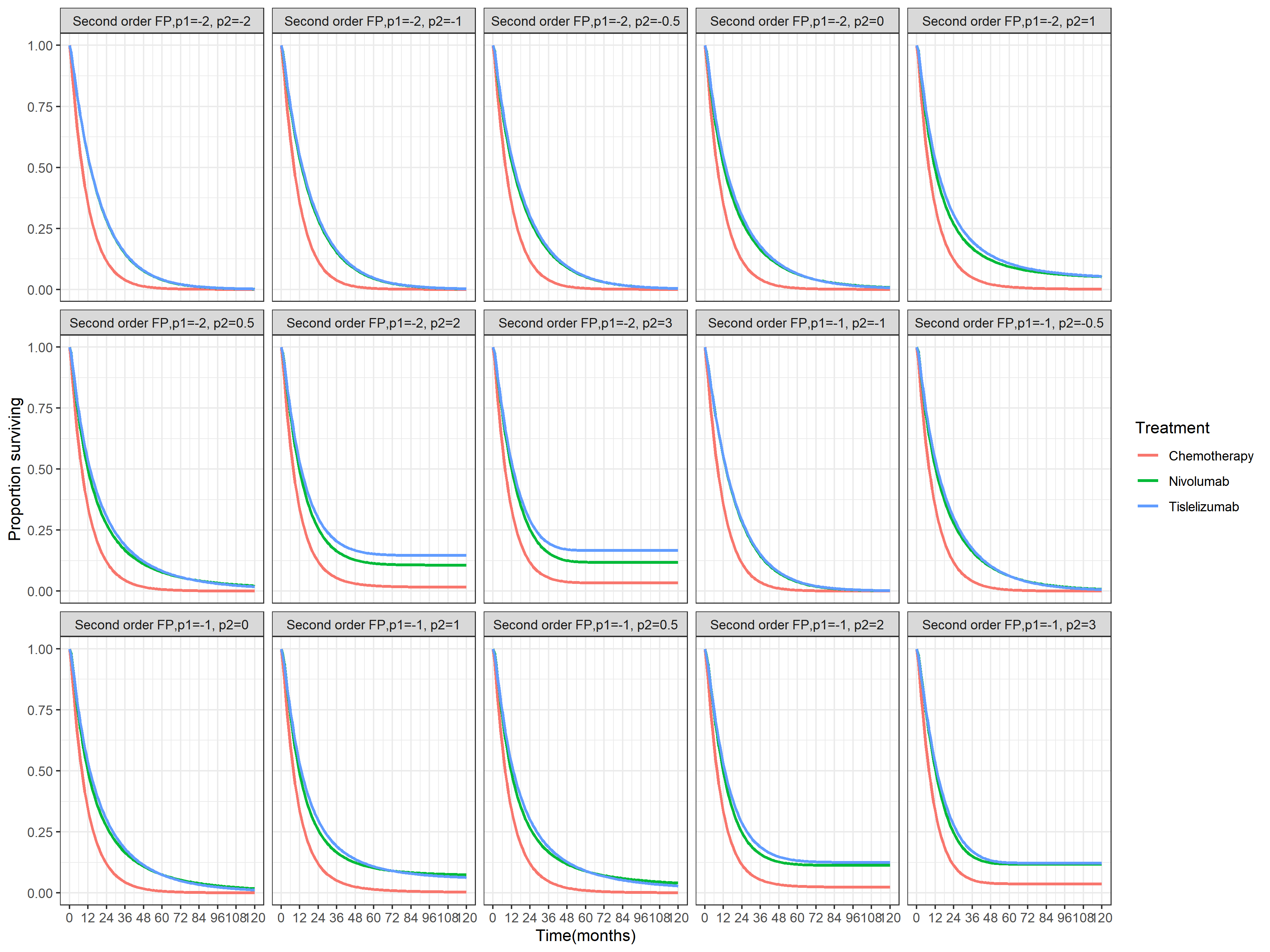
**

**
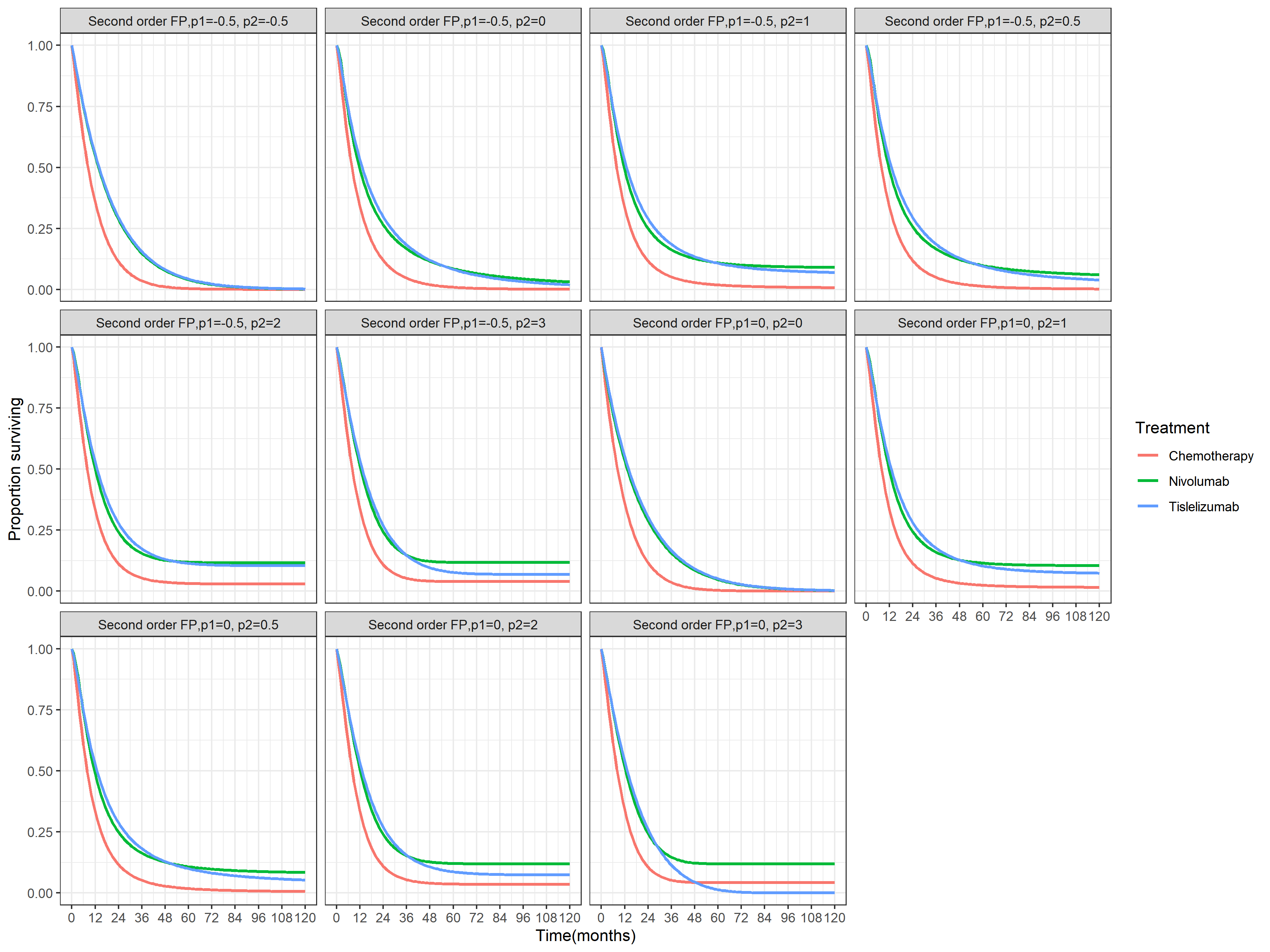
**

**
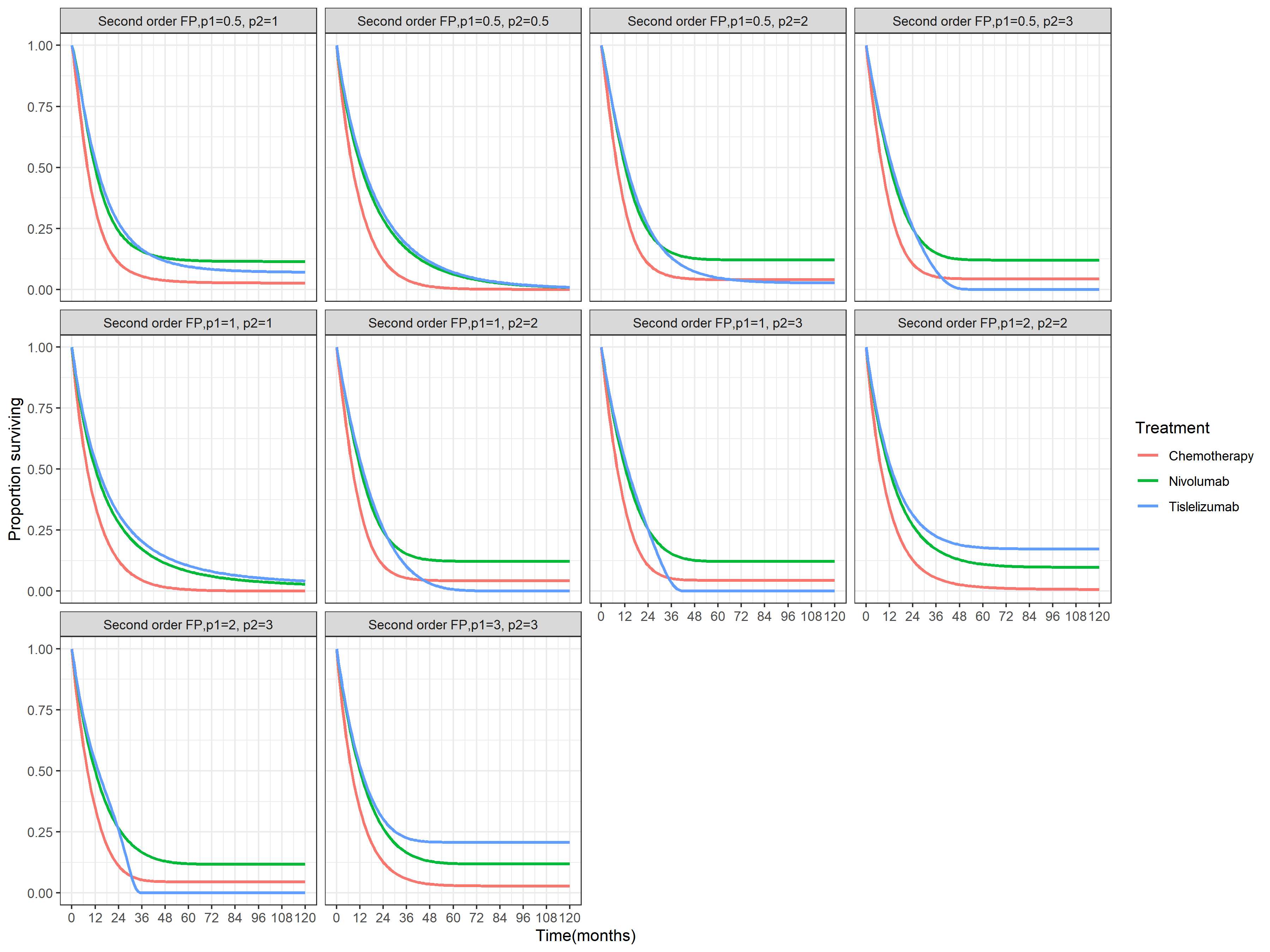
**

**eFigure 7B Second-order Fractional Polynomial Models (OS)**

**
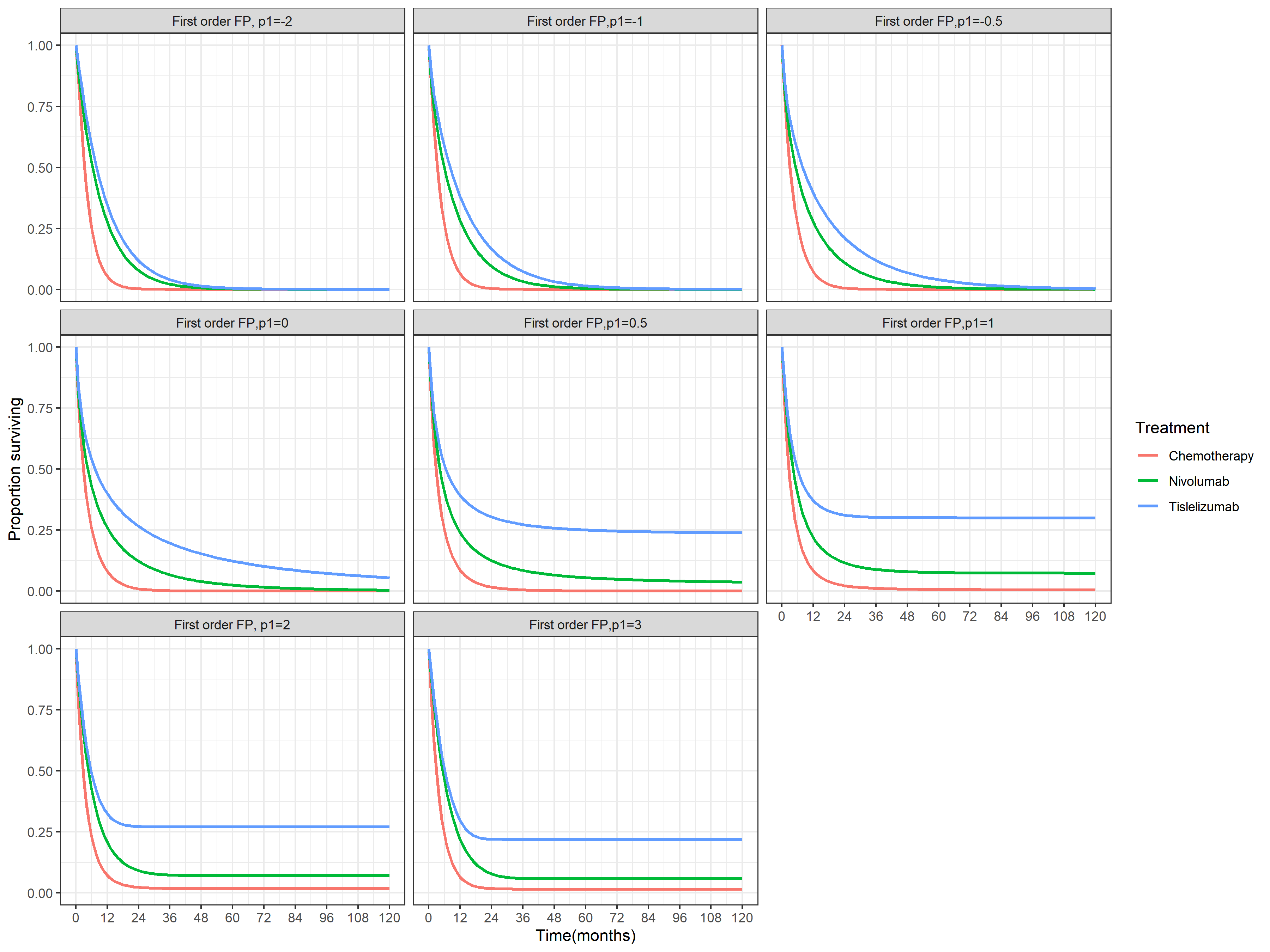
**

**eFigure 7C First-order Fractional Polynomial Models (PFS)**

**
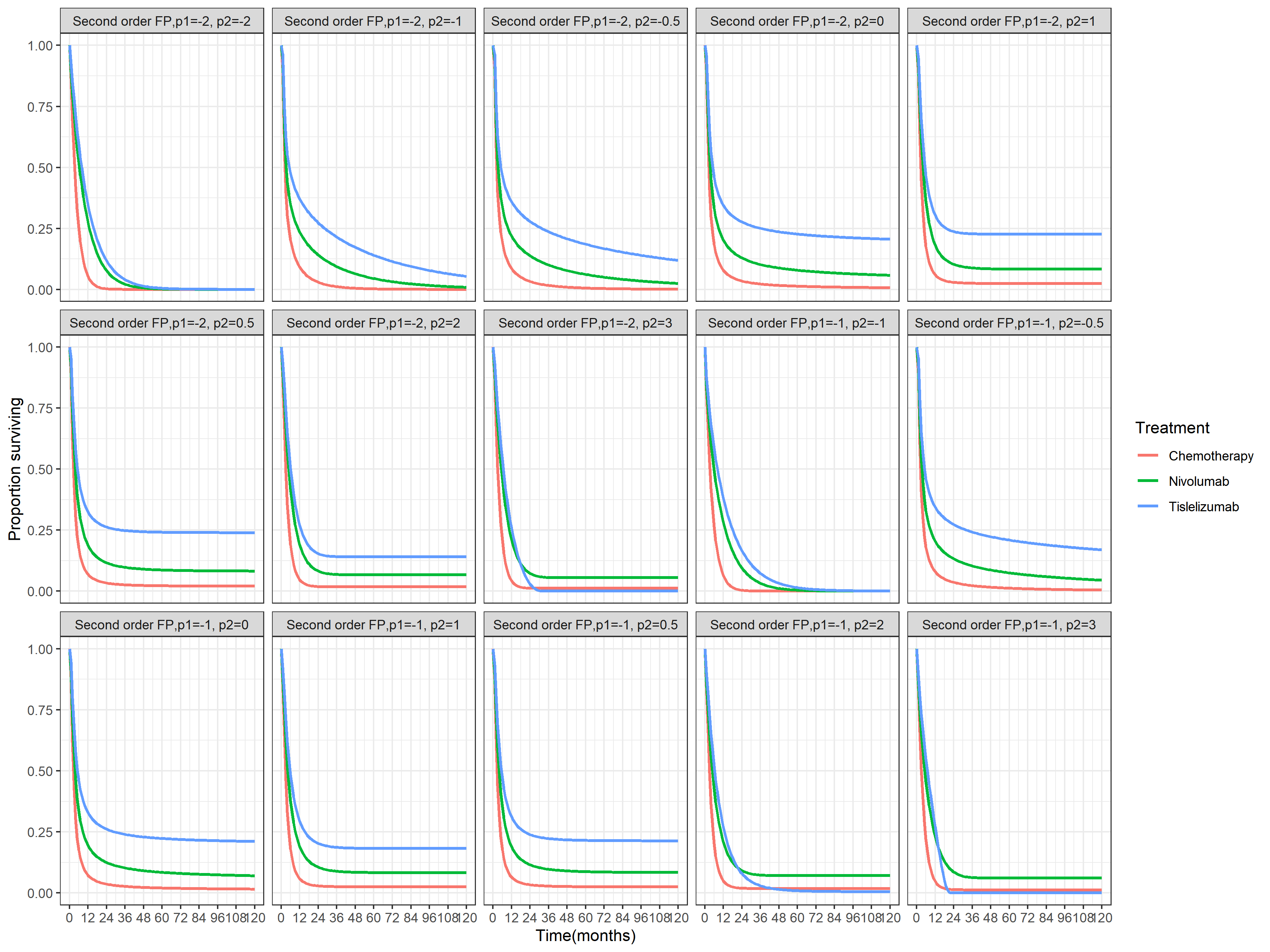
**

**
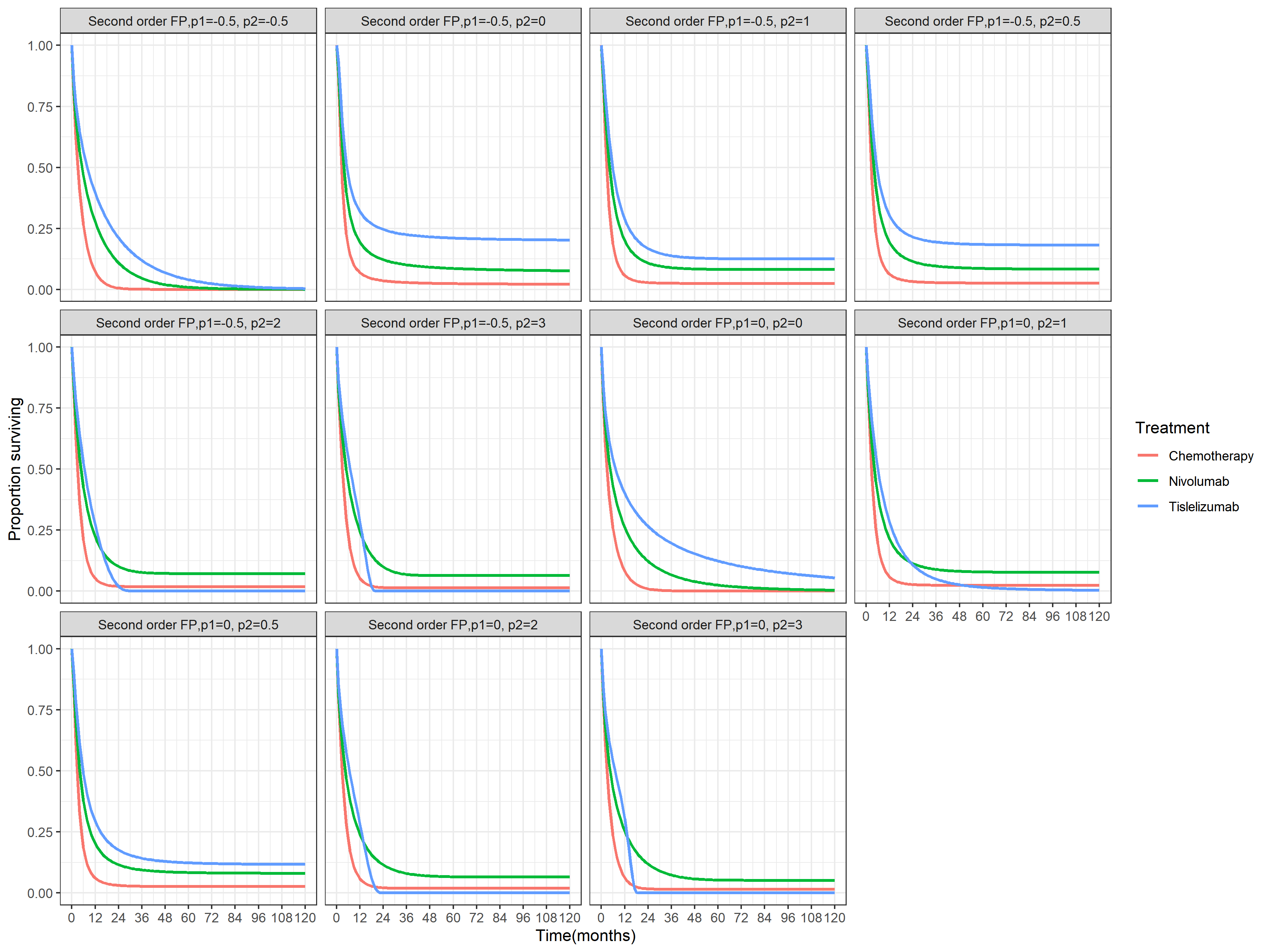
**

**
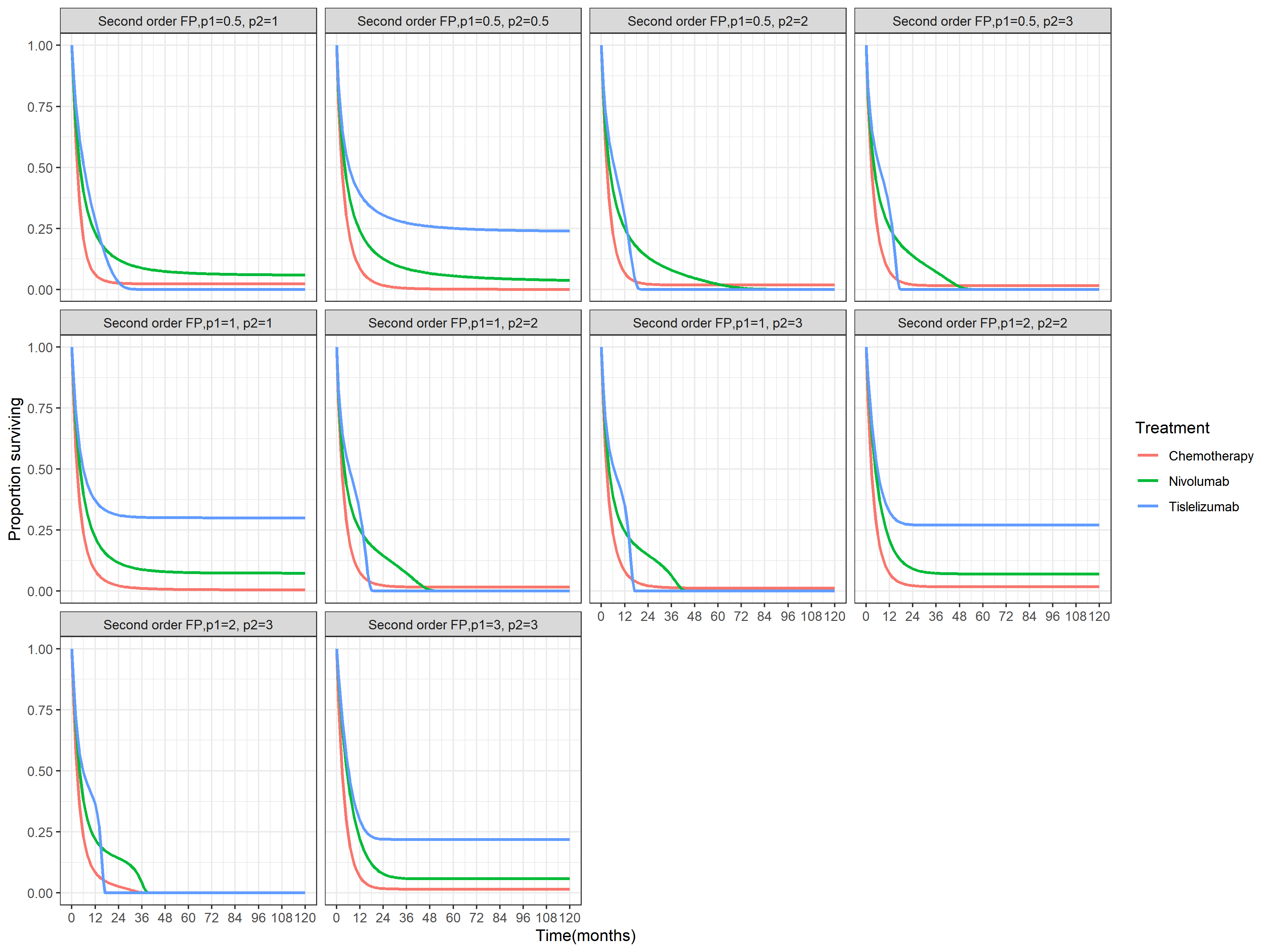
**

**eFigure 7D Second-order Fractional Polynomial Models (PFS)**

# Kaplan-Meier Survival Curves and Parametric Reconstruction Survival Curves of Each Treatment (eFigure 8)


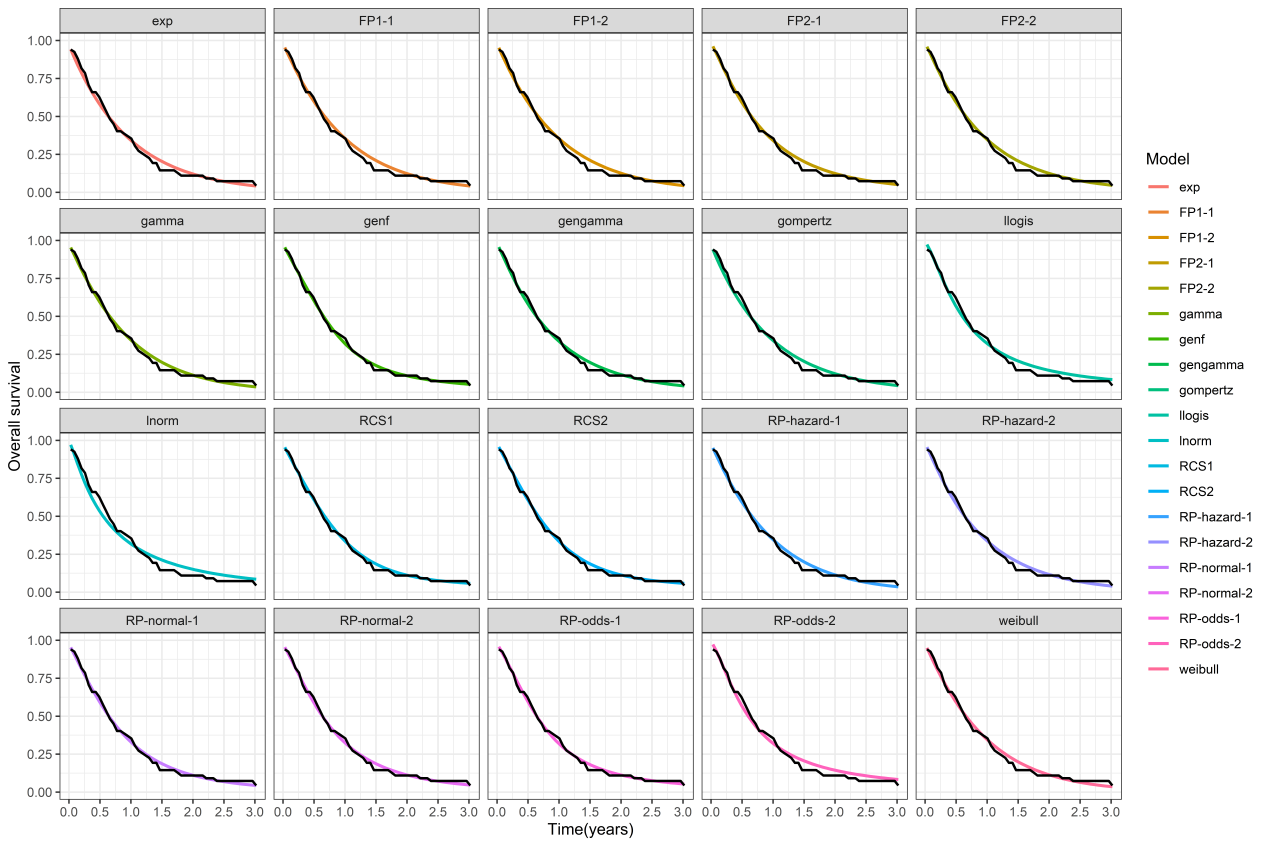


**eFigure 8A Overall Survival Curves of Docetaxel in Checkmate-078 China**

**
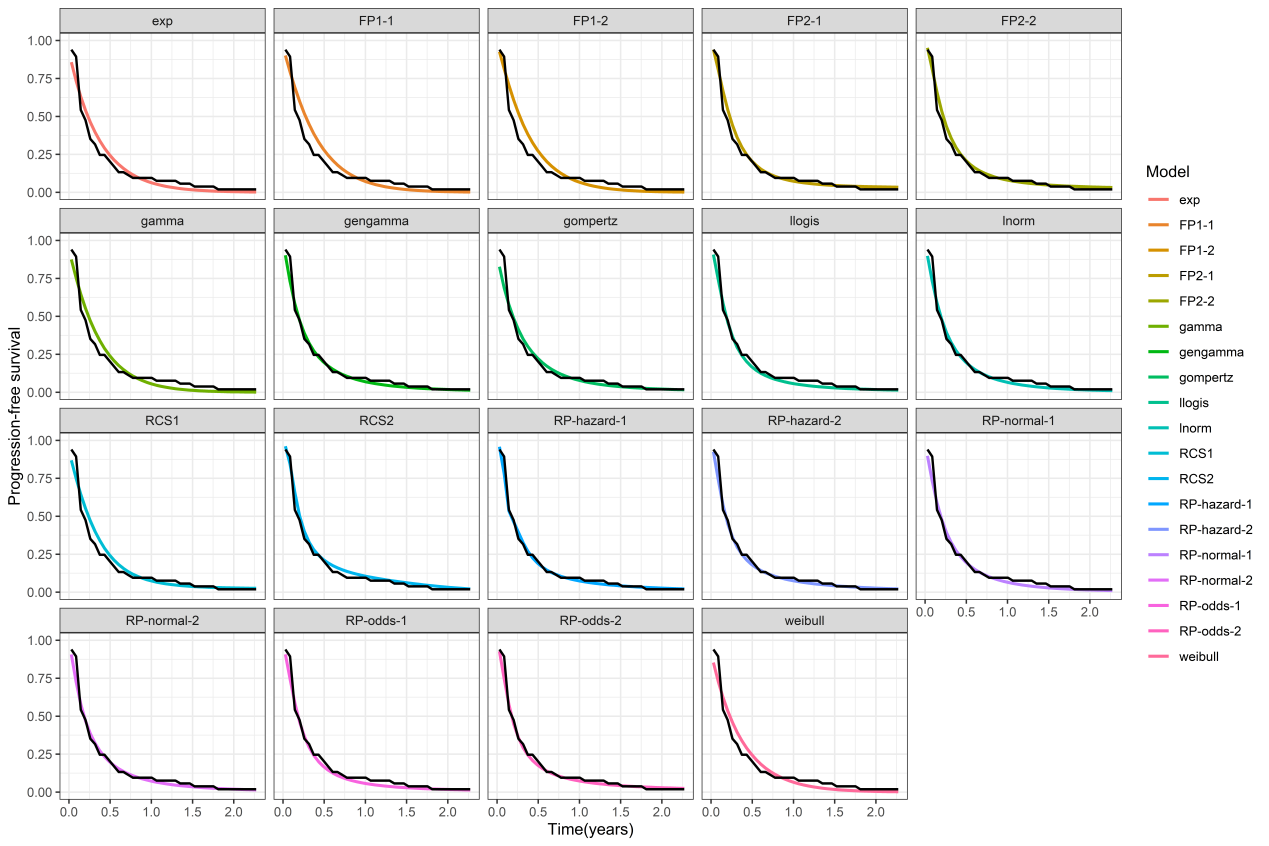
**

**eFigure 8B Progression-free Survival Curves of Docetaxel in Checkmate-078 China**

**
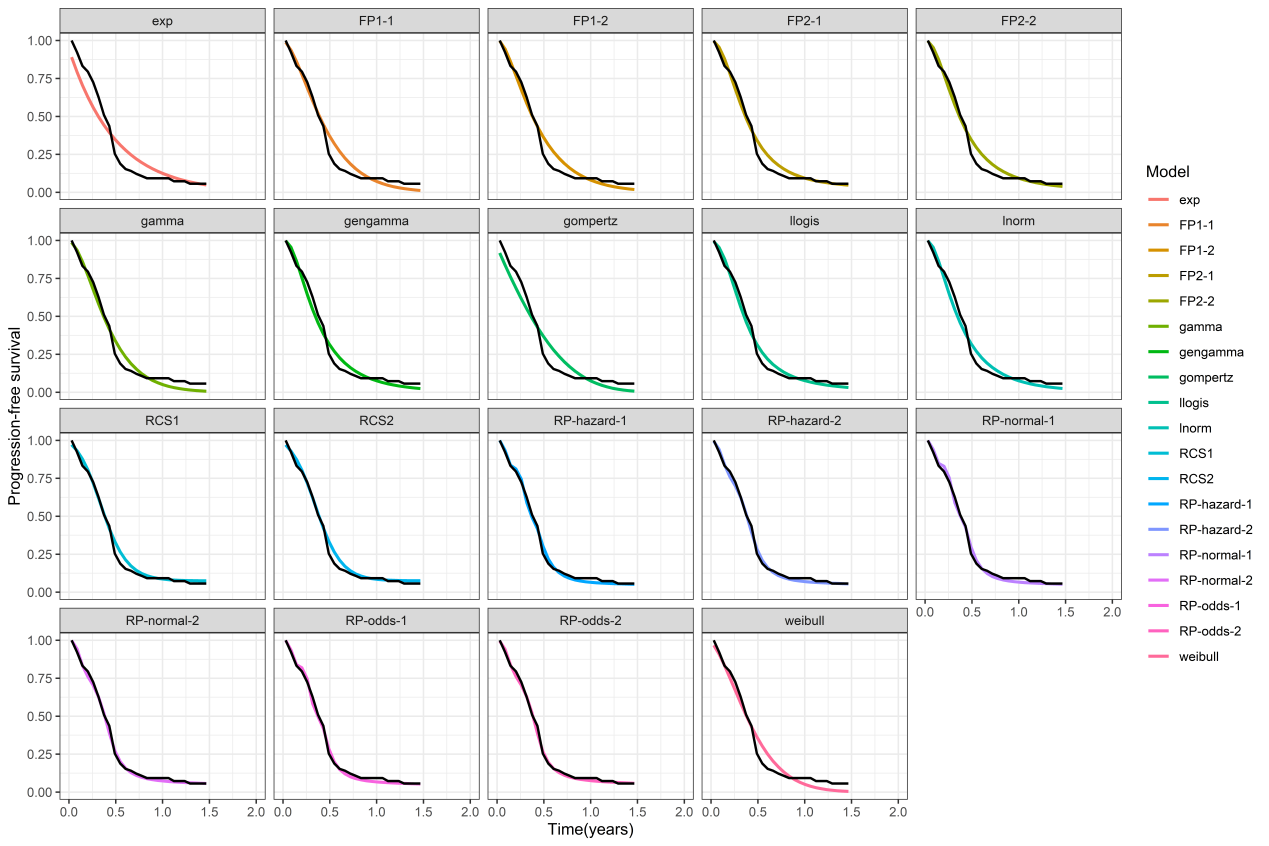
**

**eFigure 8C Progression-free Survival Curves of Chemotherapy in CameL-sq**

(RP, Royston-Parmar spline; RCS, restricted cubic spline; FP, fractional polynomial; exp, exponential; log-logistic, log-logistic; log-normal, log-normal; gengamma, generalized gamma)

# Deviance Information Criterion (DIC) for Network Meta-analysis (eTable 1)

| First-order fractional polynomial models for first-line PFS curves | | | | |
| --- | --- | --- | --- | --- |
| p | Parameter | | DIC | |
| -2 | 2 | | 1101.03 | |
| -1 | 2 | | 1129.19 | |
| -0.5 | 2 | | 1176.09 | |
| 0 | 2 | | 1244.93 | |
| 0.5 | 2 | | 1321.71 | |
| 1 | 2 | | 1389.84 | |
| 2 | 2 | | 1475.09 | |
| 3 | 2 | | 1510.44 | |
| First-order fractional polynomial models for second-line PFS curves | | | | |
| p | Parameter | | DIC | |
| -2 | 2 | | 1313.57 | |
| -1 | 2 | | 1346.54 | |
| -0.5 | 2 | | 1396.39 | |
| 0 | 2 | | 1466.94 | |
| 0.5 | 2 | | 1542.91 | |
| 1 | 2 | | 1608.43 | |
| 2 | 2 | | 1689.03 | |
| 3 | 2 | | 1723.16 | |
| Second-order fractional polynomial models for second-line OS curves | | | | |
| p_1_, p_2_ | | 3 | | 1313.57 |
| -2, -2 | | 3 | | 1289.02 |
| -2, -1 | | 3 | | 1282.53 |
| -2, -0.5 | | 3 | | 1274.81 |
| -2, 0 | | 3 | | 1260.28 |
| -2, 0.5 | | 3 | | 1266.90 |
| -2, 1 | | 3 | | 1254.68 |
| -2, 2 | | 3 | | 1258.46 |
| -2, 3 | | 3 | | 1346.54 |
| -1, -1 | | 3 | | 1263.90 |
| -1, -0.5 | | 3 | | 1253.15 |
| -1, 0 | | 3 | | 1237.39 |
| -1, 0.5 | | 3 | | 1243.73 |
| -1,1 | | 3 | | 1236.42 |
| -1,2 | | 3 | | 1247.36 |
| -1,3 | | 3 | | 1396.39 |
| -0.5, -0.5 | | 3 | | 1242.17 |
| -0.5, 0 | | 3 | | 1231.12 |
| -0.5, 0.5 | | 3 | | 1234.47 |
| -0.5, 1 | | 3 | | 1238.20 |
| -0.5, 2 | | 3 | | 1257.54 |
| -0.5, 3 | | 3 | | 1466.94 |
| 0, 0 | | 3 | | 1231.99 |
| 0, 0.5 | | 3 | | 1230.00 |
| 0, 1 | | 3 | | 1251.32 |
| 0, 2 | | 3 | | 1281.96 |
| 0, 3 | | 3 | | 1241.42 |
| 0.5, 0.5 | | 3 | | 1542.91 |
| 0.5, 1 | | 3 | | 1275.48 |
| 0.5, 2 | | 3 | | 1318.29 |
| 0.5, 3 | | 3 | | 1608.43 |
| 1, 1 | | 3 | | 1307.47 |
| 1, 2 | | 3 | | 1361.10 |
| 1, 3 | | 3 | | 1689.03 |
| 2, 2 | | 3 | | 1447.67 |
| 2, 3 | | 3 | | 1723.16 |
| 3, 3 | | 3 | | 1313.57 |

# Akaike Information Criterion (AIC) for the Referred PFS and OS Curves (eTable 2)

| For PFS curve of first-line standard chemotherapy in CameL-sq | | |
| --- | --- | --- |
| Model | Params | AIC |
| exponential | 1 | 182.36 |
| gompertz | 2 | 171.40 |
| weibull | 2 | 144.20 |
| FP1-second smallest DIC | 2 | 140.01 |
| FP1-smallest DIC | 2 | 135.24 |
| gamma | 2 | 133.73 |
| FP2-second smallest DIC | 3 | 132.95 |
| FP2-smallest DIC | 3 | 130.54 |
| gengamma | 3 | 129.96 |
| log-normal | 2 | 128.02 |
| RCS-second smallest AIC | 4 | 127.05 |
| RCS-smallest AIC | 3 | 125.06 |
| log-logistic | 2 | 124.64 |
| RP-hazard-second smallest AIC | 6 | 107.65 |
| RP-normal-second smallest AIC | 7 | 106.10 |
| RP-odds-second smallest AIC | 6 | 105.67 |
| RP-odds-smallest AIC | 7 | 104.56 |
| RP-hazard-smallest AIC | 7 | 104.27 |
| RP-normal-smallest AIC | 6 | 103.78 |
| For OS curve of second-line docetaxel in Checkmate-078 China | | |
| log-normal | 2 | 122.55 |
| RP-normal-second smallest AIC | 4 | 120.46 |
| genf | 4 | 120.13 |
| RCS-second smallest AIC | 4 | 120.07 |
| RP-hazard-second smallest AIC | 3 | 119.72 |
| gengamma | 3 | 119.59 |
| FP2-second smallest AIC | 3 | 119.48 |
| RP-odds-second smallest AIC | 2 | 119.38 |
| log-logistic | 2 | 119.38 |
| FP2-smallest AIC | 3 | 119.12 |
| RP-normal-smallest AIC | 3 | 118.58 |
| RP-odds-smallest AIC | 3 | 118.14 |
| RCS-smallest AIC | 3 | 118.10 |
| gompertz | 2 | 118.07 |
| weibull | 2 | 117.89 |
| RP-hazard-smallest AIC | 2 | 117.89 |
| FP1-second smallest AIC | 2 | 117.83 |
| gamma | 2 | 117.78 |
| FP1-smallest AIC | 2 | 117.70 |
| exponential | 1 | 116.09 |
| For PFS curve of second-line docetaxel in Checkmate-078 China | | |
| FP1-second smallest AIC | 2 | 105.04 |
| RCS-second smallest AIC | 3 | 104.09 |
| FP1-smallest AIC | 2 | 102.95 |
| weibull | 2 | 102.11 |
| gamma | 2 | 101.65 |
| exponential | 1 | 100.10 |
| gompertz | 2 | 99.87 |
| FP2-second smallest AIC | 3 | 94.14 |
| gengamma | 3 | 93.57 |
| RP-normal-second smallest AIC | 3 | 93.29 |
| FP2-smallest AIC | 3 | 92.66 |
| log-normal | 2 | 91.96 |
| RP-normal-smallest AIC | 2 | 91.96 |
| RP-hazard-second smallest AIC | 6 | 91.04 |
| RP-hazard-smallest AIC | 3 | 90.67 |
| RP-odds-second smallest AIC | 3 | 90.66 |
| log-logistic | 2 | 89.99 |
| RP-odds-smallest AIC | 2 | 89.99 |
| RCS-smallest AIC | 4 | 88.02 |

# eMethod 5: Selection Process for Parametric Survival Models and Model Validation

**Part A. NMA of first-line PFS curves: First-order fractional polynomial models (power parameter = −2)**

According to Log Cumulative Hazards Plots **(eFigure 5A)** and Schoenfeld Residual Test Plots **(eFigure 5B)**, the first-line PFS curves did not satisfy proportional hazard (PH) assumption. Therefore, we considered non-PH models. The fitting effects of the FP models on the hazard function are more flexible than other non-PH models (such as parametric survival curve models and piecewise exponential models)^[4]^. In view of the large number of survival curves and more diverse survival risk functions in this study, we chose the FP model. We considered but did not use the second-order FP models which have lower DIC values (Lower DIC values indicate better relative fit^[5]^) because the fitted survival curves violated the clinical reality distinctly: According to **eFigure 6B and eTable 1,** the second-order FP models with smaller DICs than the best-fitted first-order FP model would lead to the plateau in some survival curves of more than 5 years. Among the first-order FP models, the model with power parameter equals to −2 had the lowest DIC, furthermore, first-order fractional polynomial (FP) models (power parameter = −2) would lead reasonable survival rates for all treatments without any unreasonable plateau (**eFigure 6A**).

**Part B. NMA of second-line PFS curves: First-order fractional polynomial models (power parameter = −2)**

According to Schoenfeld Residual Test Plots **(eFigure 5B)**, though the second-line PFS curves satisfied PH assumption on the whole. However, as we can find from Log Cumulative Hazards Plots **(eFigure 5A),** the constant hazard ratio (HR) did not accurately reflect the relative risk at each point in time, so we still considered non-PH models. For the same reason in **Part A** (Selection process for NMA of first-line PFS curves**)**, we chose the FP models. According to **eFigure 7D and eTable 1,** some second-order FP models with smaller DICs than the best-fitted first-order FP model would lead to the plateau in some survival curves of more than 5 years, while the other second-order FP models which had smaller DICs than the best-fitted first-order FP model would result in a 10-year progression-free survival rate of more than 5% for nivolumab. According to Lung-MAP S1400I^[6]^, the 3-year PFS rate of nivolumab was close to 0, thus the 2nd order model was considered unsuitable for this NMA. first-order fractional polynomial models (power parameter = −2) had the lowest DIC among all 1-order FP models and reasonable fitting and extrapolation of survival curves (**eFigure 7C**) were considered to be the best model for NMA of second-line PFS curves.

**Part C. NMA of second-line PFS curves: Second-order fractional polynomial models (power parameters equal to −0.5 and 0, respectively)**

For the same reason in **Part B** (Selection process for NMA of second-line PFS curves**)**, we chose the FP models. According to **eFigure 7B and eTable 1,** some second-order FP models with smaller DICs that led to the plateau in some survival curves of more than 5 years were excluded. The other second-order FP models which had smaller DICs than the second-order FP model (P1 = 0, P2 = 1) overall survival rate of more than 8% for nivolumab, equals to the 3.2-year overall survival rates of nivolumab according to Lung-MAP S1400I^[6]^. Thus second-order fractional polynomial model (P1 = 0, P2 = 1) was the best-fitted model for this NMA, first-order fractional polynomial models were not chosen because of higher DICs compared to it.

**Part D. Parametric first-line referred PFS curves: Log-logistic model**

We considered the following parametric functions: exponential, Weibull, Gompertz, Gamma, Log-logistic, Log-normal, Generalized Gamma, GenF, FP, Restricted Cubic Spline (RCS, Knots = 0~5), and Royston-Parmar splines (RP) models (Knots = 0~5, link function = Normal, Odds or Hazards). In the first step, in order to reduce the number of included models, for the 1-order FP, 2-order FP, RCS, RP-Odd, RP-Normal, RP-Hazards models, we only consider the two models with the lowest AIC in each class. According to **eTable 2** and **eFigure 8C**, among the 20 chosen parametric models, log-logistic was the model with the 6th smallest AIC, second only to the Royston-Parmar splines models. We considered but not chose the RP models because the RP models resulted in a underfit to survival curves of pembrolizumab or sugalimumab combined with chemotherapy after visual inspection of observed KM curves in Keynote-407 China and Gemstone-302 in our network^[7, 8]^.

**Part E. Parametric second-line referred OS curve: Exponential model**

Similar to **Part D**, according to **eTable 2** and **eFigure 8A**, among the 20 chosen parametric models, exponential model was the model with the smallest AIC and the best fit. Furthermore, the exponential models resulted in a good-fit to survival curves of all treatments after visual inspection of observed OS curves without any unreasonable plateau or survival rates at any time.

**Part F. Parametric second-line referred PFS curve: RCS model**

The RCS model with 2 knots had the smallest AIC compared with other 19 models, the corresponding survival curves were also in line with observed PFS curves in the network after visual inspection. So for the same reason in **Part E**, the RCS model was chosen to be the parametric model for the referred second-line PFS curve.

**Model validation**

Our model was validated following the ISPOR Task Force recommendation^[13]^.

After indirect comparsions, for first-line PFS curves, the median PFS time of standard chemotherapy was 4.9 months (4.2~5.5) and 4.5 months in CameL-sq^[18]^ and our mdoel; The median PFS time of camrelizumab plus standard chemotherapy was 8.5 months (6.9~10.4) and 9.1 months in CameL-sq^[18]^ and our mdoel; The median PFS time of sintilimab plus standard chemotherapy was 5.5 months (4.9~6.8) and 6.3 months in Orient 12^[14]^ and our mdoel; The median PFS time of tislelizumab plus standard chemotherapy was 7.6 months (6~9.8) and 6.3 months in Rationale 307^[15]^ and our mdoel; The median PFS time of sugemalimab plus standard chemotherapy was 8.3 months (6.9~10.9) and 9.8 months in Gemstone 302^[7]^ and our mdoel; The median PFS time of pembrolizumab plus standard chemotherapy was 8.3 months (6.2~10.5) and 10.4 months in Keynote 407 China^[8]^ and our mdoel; The median PFS time of nedaplatin plus standard chemotherapy was 4.6 months (4.4~5.1) and 4.9 months in Just^[17]^ and our mdoel. Among second-line PFS and OS curves, the median PFS time of docetaxel was 2.7 months (1.4~2.9) and 2.5 months in Checkmate 078 China^[16]^ and our mdoel; The median OS time of docetaxel was 7.9 months (5.9~11.2) and 7.7 months in Checkmate 078 China^[16]^ and our mdoel; The PFS rate of tislelizumab at 6.2 months was 50% in Rationale 303^[19]^, similar to 55% in our model; and PFS rate of nivolumab at 2.9 months was 50% in Checkmate-078 China^[16]^, similar to 60% in our model; The median OS time of tislelizumab was 16.0 months (13.8~18.9) and 14.6 months in Rationale 303^[19]^ and our mdoel; The median OS time of nivolumab was 11.7 months (8.9~14.0) and 11.8 months in Checkmate 078 China^[16]^ and our model.

Considering that the survival rates of each treatment in our model would definitely be different from the survival rates in RCTs after indirect comparisons, simulated clinical outcomes in our model were consistent with the results of respective clinical trials.

Although the Orient 12^[14]^, Gemstone 302^[7]^, Rationale 307^[19]^ and Just^[17]^ did not report median OS time for sq-NSCLC, the mean and media OS time was 2.83 and 2.23 years for patients treated with pembrolizumab plus standard chemotherapy estimated by our model, respectively, matched the median OS time of 2.51 years reported in Keynote 407 China^[8]^; The mean and media OS time was 1.42 and 1.15 years for patients treated with standard chemotherapy estimated by our model, respectively, matched the mean and median OS time of 1.47 and 1.29 years reported in Shao et al.^[12]^ and CameL-sq^[20]^, respectively; The mean and media OS time was 2.51 and 2.01 years for patients treated with camrelizumab plus standard chemotherapy estimated by our model, respectively, matched the mean and media OS time of 2.38 and 2.29 years reported in Shao et al.^[12]^ and CameL-sq^[20]^, respectively. The model results were consistent with the RCTs, but minor deviations still exist. This is partly due to the bias introduced by NMA, and partly due to the differences in second-line treatments, such as a certain percents of patients were treated with second-line immunotherapy in RCTs.

A recent research by Shao et al.^[12]^ evaluated the cost-effectiveness of camrelizumab plus chemotherapy to treat sq-NSCLC from the perspective of Chinese healthcare system, using head-to-head RCT data, the ICER of camrelizumab combined with standard chemotherapy compared to standard chemotherapy was estimated to be USD 13,572, similar to our findings (USD 12,276). There is no research related to other drugs as July 2022, so no more external verification can be carried out.

# eMethod 6: Details for Patient Assistance Programs

Considering that sugemalimab, nivolumab, and pembrolizumab have patient assistance programs for patients with advanced non-small cell lung cancer. Considering PAP, the prices of the above drugs were different due to patient survival time. Followings are the details of PAP for each drug.

**Pembrolizumab**^[9]^

According to the newest PAP (program name: KEY TO LIFE) of pembrolizumab in 2021, patients who bought the first two cycles of pembrolizumab would receive the next two cycles for free, continuing to buy the two cycles of pembrolizumab; next cycles remaining for up to 2 years (35 cycles) would also be free.

**Nivolumab**^[10]^

The first stage, after receiving 3 consecutive nivolumab treatments, patients can continue to benefit from nivolumab treatment and have no disease progression after being assessed by designated physicians, get free drug assistance for up to 3 subsequent treatments. The second stage, patients who have completed the first stage, if they still need to receive nivolumab treatment, can continue to benefit from nivolumab treatment and have no disease progression after receiving 3 consecutive nivolumab treatments after being evaluated by the designated physician. After being approved by the PMO, the patient can receive drug assistance for the remainder of the application year at most. Patients can apply according to the application year cycle until the project is terminated or the disease progresses.

**Sugemalimab**^[11]^

The first round of assistance: After at least 2 treatment cycles of sugemalimab, the patient still needs to continue treatment with sugemalimab after evaluation by the project-designated doctor, after being reviewed and approved by the project office, a maximum of 2 treatment cycles of sugemalimab can be obtained as an aid drug. The second round of assistance: After the first round of assistance, the patients who still need to continue treatment with sugemalimab after the evaluation of the project doctor, and who have the intention to apply, have undergone at least 2 treatment cycles of sugemalimab. After sugemalimab treatment, after being reviewed and approved by the project office, a maximum of 11 treatment cycles of sugemalimab injection can be obtained as an aid drug. The third round of assistance: After the second round of assistance, the patients who still need to continue treatment with sugemalimab after the evaluation of the project doctor, and who have the intention to apply, have undergone at least one treatment cycle of sugemalimab. After sugemalimab treatment, after being reviewed and approved by the project office, a maximum of 8 treatment cycles of sugemalimab can be obtained as an aid drug.

**References**

[1] Criss S D, Mooradian M J, Sheehan D F, et al. Cost-effectiveness and Budgetary Consequence Analysis of Durvalumab Consolidation Therapy vs No Consolidation Therapy After Chemoradiotherapy in Stage III Non-Small Cell Lung Cancer in the Context of the US Health Care System[J]. JAMA Oncol, 2019,5(3):358-365.

[2] Data T T N P. http://www.stats.gov.cn/tjsj/pcsj/rkpc/6rp/indexch.htm.[EB/OL]. http://www.stats.gov.cn/tjsj/pcsj/rkpc/6rp/indexch.htm.

[3] Pollock R F, Norrbacka K, Boye K S, et al. The PRIME Type 2 Diabetes Model: a novel, patient-level model for estimating long-term clinical and cost outcomes in patients with type 2 diabetes mellitus[J]. J Med Econ, 2022,25(1):393-402.

[4] Jansen J P. Network meta-analysis of survival data with fractional polynomials[J]. BMC Med Res Methodol, 2011,11:61.

[5] Rutherford M J, Lambert P C, Sweeting M J, et al. NICE DSU Technical Support Document 21. Flexible Methods for Survival Analysis[J]. Department of Health Sciences, University of Leicester, Leicester, UK, 2020:1-97.

[6] Gettinger S N, Redman M W, Bazhenova L, et al. Nivolumab Plus Ipilimumab vs Nivolumab for Previously Treated Patients With Stage IV Squamous Cell Lung Cancer: The Lung-MAP S1400I Phase 3 Randomized Clinical Trial[J]. JAMA Oncol, 2021,7(9):1368-1377.

[7] Zhou C, Wang Z, Sun Y, et al. Sugemalimab versus placebo, in combination with platinum-based chemotherapy, as first-line treatment of metastatic non-small-cell lung cancer (GEMSTONE-302): interim and final analyses of a double-blind, randomised, phase 3 clinical trial[J]. Lancet Oncol, 2022,23(2):220-233.

[8] Cheng Y, Zhang L, Hu J, et al. Pembrolizumab Plus Chemotherapy for Chinese Patients With Metastatic Squamous NSCLC in KEYNOTE-407[J]. JTO Clin Res Rep, 2021,2(10):100225.

[9] The key to life-tumor immunotherapy patient assistance project project assistance plan adjustment announcement[EB/OL].http://smzy.ilvzhou.com/index.php?a=lists&c=index&catid=12&m=content.

[10] Announcement on the Adjustment of the Assistance Program of the Odivo Patient Assistance Program[EB/OL]. http://www.cfchina.org.cn/show.php?contentid=2213.

[11] "Ze Mei Tong Xing" Charity Drug Donation Project[EB/OL]. https://zmtx.ilvzhou.com/?page_id=126.

[12] Shao T, Ren Y, Zhao M, et al. Cost-effectiveness Analysis of Camrelizumab Plus Chemotherapy as First-Line Treatment for Advanced Squamous NSCLC in China. Front. Public Health 10:912921. https://www.frontiersin.org/articles/10.3389/fpubh.2022.912921/abstract

[13] Eddy, D. M., Hollingworth, W., Caro, J, et al. (2012). Model Transparency and Validation: A Report of the ISPORSMDM Modeling Good Research Practices Task Force--7. Value Health 15 (6), 843-850.

[14] Zhou C, Wu L, Fan Y, et al. Sintilimab Plus Platinum and Gemcitabine as First-Line Treatment for Advanced or Metastatic Squamous NSCLC: Results From a Randomized, Double-Blind, Phase 3 Trial (ORIENT-12). J THORAC ONCOL. 2021; 16: 1501-11.

[15] Wang J, Lu S, Yu X, et al. Tislelizumab Plus Chemotherapy vs Chemotherapy Alone as First-line Treatment for Advanced Squamous Non-Small-Cell Lung Cancer: A Phase 3 Randomized Clinical Trial. JAMA ONCOL. 2021; 7: 709-17.

[16] Chang J, Wu YL, Lu S, et al. Three-year follow-up and patient-reported outcomes from CheckMate 078: Nivolumab versus docetaxel in a predominantly Chinese patient population with previously treated advanced non-small cell lung cancer. LUNG CANCER. 2021; 165: 71-81.

[17] Lu S, Chen Z, Hu C, et al. Nedaplatin plus docetaxel versus cisplatin plus docetaxel as first-line chemotherapy for advanced squamous cell carcinoma of the lung—a multicenter, open-label, randomized, phase III trial. J THORAC ONCOL. 2018; 13: 1743-49.

[18] Ren S, Chen J, Xu X, et al. Camrelizumab Plus Carboplatin and Paclitaxel as First-Line Treatment for Advanced Squamous NSCLC (CameL-Sq): A Phase 3 Trial. J THORAC ONCOL. 2022; 17: 544-57.

[19] Zhou C. Results from RATIONALE 303: A global Phase 3 study of tislelizumab vs docetaxel as second- or third-line therapy for patients with locally advanced or metastatic NSCLC. AACR 2021 CT039.

[20] Zhou C. Updated OS for CameL-sq: Camrelizumab Plus Carboplatin and Paclitaxel as First-Line Treatment for Advanced Squamous NSCLC (CameL-Sq): A Phase 3 Trial. ELCC 2022.
