# Supplementary material for: Effectiveness and cost-effectiveness analysis of 11 treatment paths, seven first-line and three second-line treatments for Chinese patients with advanced wild-type squamous non-small cell lung cancer: A sequential model
Source: Front Public Health. 2023 Feb 24;11:1051484. doi: 10.3389/fpubh.2023.1051484 (PMC9999022; doi:10.3389/fpubh.2023.1051484)
Supplement: Supplementary file 4 [file Data_Sheet_4.DOCX]

**Supplement 3**

[eTable 1 Costs and Effectiveness Breakdown Results 2](#_Toc2035)

[eTable 2 Efectiveness and Cost-effectiveness Analysis Results of First-line Treatment Regimens 3](#_Toc22712)

[eTable 3 Efectiveness and Cost-effectiveness Analysis Results of Second-line Treatment Regimens 3](#_Toc18150)

[eTable 4 Results for Scenario Analysis 5](#_Toc13845)

[eFigure 1 Net Monetary Benefits of All Treatments over Time 8](#_Toc27086)

[eFigure 2 Net monetary benefits of All Treatments When Costs of Third-line treatment Changed 9](#_Toc32643)

### eTable 1 Costs and Effectiveness Breakdown Results

| Treatment | NN | ND | NT | SI | PE | TI | CA | SU | CN | CD | CT |
| --- | --- | --- | --- | --- | --- | --- | --- | --- | --- | --- | --- |
| Total cost/$ | 61497.63 | 9899.64 | 17764.81 | 15855.26 | 117368.77 | 16071.79 | 19026.16 | 80927.12 | 62227.29 | 9981.14 | 18130.87 |
| AE management costs/$ | 52.00 | 332.09 | 198.07 | 1542.64 | 518.60 | 465.82 | 496.77 | 510.29 | 686.05 | 966.14 | 832.12 |
| Cost for end-of-care/$ | 2162.76 | 2268.86 | 2183.76 | 2211.21 | 2120.09 | 2225.90 | 2154.27 | 2134.28 | 2168.71 | 2274.57 | 2186.14 |
| Drug costs/$ | 55233.20 | 4654.19 | 11631.98 | 8639.69 | 109967.32 | 10125.21 | 12102.32 | 73722.82 | 55419.30 | 4196.72 | 11400.11 |
| Follow-up costs/$ | 2089.69 | 1432.85 | 1929.97 | 1952.54 | 2777.07 | 1818.16 | 2467.38 | 2645.80 | 2030.18 | 1371.28 | 1896.11 |
| Disease management costs/$ | 1959.99 | 1211.64 | 1821.03 | 1509.19 | 1985.68 | 1436.70 | 1805.42 | 1913.94 | 1923.05 | 1172.42 | 1816.38 |
| Cost during progression-free stage/$ | 1670.48 | 1683.40 | 1682.44 | 7918.80 | 109796.81 | 7926.35 | 11268.66 | 73210.64 | 1803.71 | 1800.78 | 1800.27 |
| Cost during progression disease stage/$ | 59827.15 | 8216.24 | 16082.37 | 7936.46 | 7571.96 | 8145.43 | 7757.50 | 7716.48 | 60423.58 | 8180.36 | 16330.60 |
| Total utility/QALY | 1.3890 | 0.9057 | 1.3010 | 1.2660 | 1.8067 | 1.1789 | 1.6025 | 1.7211 | 1.3527 | 0.8657 | 1.2832 |
| Utility during progression-free stage/QALY | 0.3426 | 0.3469 | 0.3472 | 0.7268 | 1.2935 | 0.6257 | 1.0793 | 1.1972 | 0.3149 | 0.3133 | 0.3132 |
| Utility during progression disease stage/QALY | 1.0464 | 0.5587 | 0.9538 | 0.5392 | 0.5133 | 0.5532 | 0.5232 | 0.5239 | 1.0378 | 0.5524 | 0.9700 |
| Disutility of AE/QALY | 0.0011 | 0.0036 | 0.0012 | 0.0071 | 0.0055 | 0.0052 | 0.0056 | 0.0052 | 0.0046 | 0.0071 | 0.0048 |

NN, first-line nedaplatin-based chemotherapy followed by second-line nivolumab; NT, first-line nedaplatin-based chemotherapy followed by second-line tislelizumab; ND, first-line nedaplatin-based chemotherapy followed by second-line docetaxel; CN, first-line standard chemotherapy followed by second-line nivolumab; CT, first-line standard chemotherapy followed by second-line tislelizumab; CD, first-line standard chemotherapy followed by second-line docetaxel; TI, first-line tislelizumab combined with chemotherapy followed by second-line docetaxel; CA, first-line camrelizumab combined with chemotherapy followed by second-line docetaxel; PE, first-line pembrolizumab combined with chemotherapy followed by second-line docetaxel; SI, first-line sintilimab combined with chemotherapy followed by second-line docetaxel; SU, first-line sugemalimab combined with chemotherapy followed by second-line docetaxel; QALY, quality-adjusted life year; AE, adverse events.

### eTable 2 Efectiveness and Cost-effectiveness Analysis Results of First-line Treatment Regimens

| Treatment | N+C | SI+C | P+C | T+C | CA+C | SU+C | Standard chemotherapy |
| --- | --- | --- | --- | --- | --- | --- | --- |
| Cost/$ | 9899.64 | 15855.26 | 117368.77 | 16071.79 | 19026.16 | 80927.12 | 62227.29 |
| Utility/QALY | 0.91 | 1.27 | 1.81 | 1.18 | 1.60 | 1.72 | 1.35 |
| NMB/$^&^ | 7391 | 8315 | -82875 | 6436 | 11569 | -48068 | -36401 |
| ICER (VS N+C) | NA | 16530^**^ | 119270 | 22590^***^ | 13096^*^ | 87102 | 117048 |
| INMB (VS CA+C) ^#^ | -4178 | -3255 | -94444 | -5134 | NA | -59637 | -47971 |

* Close to 1 time the 2021 GDP per capita ($12,728);

** 1~1.5 times 2021 GDP per capita ($12,728-19,092);

*** 1.5~3 times 2021 Gross Domestic Product (GDP) per capita ($19,092-38,184);

& Cost-effective threshold = 1.5 times 2021 Gross Domestic Product per capita ($19,092)

# CA+C had the largest NMB

N+C, Nedaplatin in combination with standard chemotherapy; SI+C, Sintilimab in combination with standard chemotherapy; P+C, Pembrolizumab in combination with standard chemotherapy; T+C, Tislelizumab in combination with standard chemotherapy; CA+C, Camrelizumab in combination with standard chemotherapy; SU+C, Sugalimumab in combination with standard chemotherapy; NMB, net monetary benefit; INMB, incremental net monetary benefit; QALY, quality-adjusted life year; ICER, incremental cost-effectiveness ratio.

### eTable 3 Efectiveness and Cost-effectiveness Analysis Results of Second-line Treatment Regimens

| Treatment | After first-line treatment with Nedaplatin in combination with standard chemotherapy | | | After first-line treatment with standard chemotherapy | | |
| --- | --- | --- | --- | --- | --- | --- |
|  | Nivolumab | Docetaxel | Tislelizumab | Nivolumab | Docetaxel | Tislelizumab |
| Progression-free survival during second-line treatment (first-stage PD in the model) | | | | | | |
| Cost/$ | 48864.93 | 583.95 | 6870.49 | 49644.95 | 569.60 | 7054.25 |
| Utility/QALY | 0.37 | 0.17 | 0.43 | 0.38 | 0.17 | 0.44 |
| NMB/$^&^ | -36775 | 4916 | 6981 | -37362 | 4793 | 7168 |
| ICER (VS Docetaxel) | 237786 | NA | 24430^**^ | 230169 | NA | 23756^**^ |
| INB (VS Tislelizumab) ^#^ | -43756 | -2065 | NA | -44530 | -2375 | NA |
| Overall survival after first-line treatment (first- and second-stage PD, and death in the model) | | | | | | |
| Cost/$ | 59827.15 | 7936.14 | 15936.30 | 60423.58 | 7900.27 | 16184.53 |
| Utility/QALY | 1.05 | 0.56 | 0.95 | 1.04 | 0.55 | 0.97 |
| NMB/$^&^ | -25866 | 10280 | 15025 | -26741 | 10111 | 15302 |
| ICER (VS Docetaxel) | 106969 | NA | 20373^*^ | 108779 | NA | 19954^*^ |
| INB (VS Tislelizumab) ^#^ | -40891 | -4745 | NA | -42042 | -5190 | NA |

* About 1.5 times the 2021 Gross Domestic Product (GDP) per capita ($19,092);

** About 2 times the 2021 Gross Domestic Product (GDP) per capita ($25,456);

& Cost-effective threshold = 2.55 times the 2021 GDP ($32,456)

# Tislelizumab had the largest NMB

NMB, net monetary benefit; INMB, incremental net monetary benefit; QALY, quality-adjusted life year; ICER, incremental cost-effectiveness ratio.

### eTable 4 Results for Scenario Analysis

| Treatment | NN | ND | NT | SID | PED | TID | CAD | SUD | CN | CD | CT |
| --- | --- | --- | --- | --- | --- | --- | --- | --- | --- | --- | --- |
| Scenario 1: utility changed (PFS:0.804, PD:0.321) | | | | | | | | | | | |
| Cost/$ | 61497.63 | 9899.64 | 17764.81 | 15855.26 | 117368.77 | 16071.79 | 19026.16 | 80927.12 | 62227.29 | 9981.14 | 18130.87 |
| Utility/QALY | 0.9630 | 0.7015 | 0.9178 | 1.0717 | 1.6649 | 0.9708 | 1.4408 | 1.5675 | 0.9270 | 0.6600 | 0.8881 |
| NMB/$^&^ | -43112 | 3493 | -243 | 4605 | -85583 | 2462 | 8481 | -51000 | -44528 | 2619 | -1175 |
| INMB (VS CAD) ^#^ | -51593 | -4988 | -8724 | -3876 | -94064 | -6019 | NA | -59481 | -53009 | -5862 | -9656 |
| ICER (VS CD) | 170003 | dominant | 30192^***^ | 14268^*^ | 106860 | 19597^**^ | 11584* | 78174 | 195621 | NA | 35718^***^ |
| ICER (VS ND) | 197296 | NA | 36363^***^ | 16088^**^ | 111549 | 22921 | 12345* | 82015 | 231979 | dominated | 44097 |
| Scenario 2: utility changed (PFS for immutherapy:0.877, PFS for chemotherapy: 0.823, first-stage PD:0.768, end-stage PD:0.703) | | | | | | | | | | | |
| Cost/$ | 61497.63 | 9899.64 | 17764.81 | 15855.26 | 117368.77 | 16071.79 | 19026.16 | 80927.12 | 62227.29 | 9981.14 | 18130.87 |
| Utility/QALY | 1.6908 | 1.0933 | 1.5919 | 1.5121 | 2.1429 | 1.4102 | 1.9041 | 2.0431 | 1.6493 | 1.0464 | 1.5732 |
| NMB/$^&^ | -29216 | 10973 | 12627 | 13014 | -76457 | 10851 | 17327 | -41921 | -30738 | 9996 | 11904 |
| INMB (VS CAD) ^#^ | -46543 | -6354 | -4700 | -4313 | -93784 | -6476 | NA | -59248 | -48065 | -7331 | -5423 |
| ICER (VS CD) | 79937 | dominant | 14269^*^ | 12612^*^ | 97938 | 16742^**^ | 10545^*^ | 71183 | 86650 | NA | 15471^*^ |
| ICER (VS ND) | 86344 | NA | 15774^*^ | 14218^**^ | 102390 | 19476^**^ | 11255^*^ | 74782 | 94101 | dominated | 17152^**^ |
| Scenario 3: patient assistance programs considered | | | | | | | | | | | |
| Cost/$ | 29583.92 | 9899.64 | 17764.81 | 15855.26 | 31974.82 | 16071.79 | 19026.16 | 37701.85 | 30006.75 | 9981.14 | 18130.87 |
| Utility/QALY | 1.3890 | 0.9057 | 1.3010 | 1.2660 | 1.8067 | 1.1789 | 1.6025 | 1.7211 | 1.3527 | 0.8657 | 1.2832 |
| NMB/$^&^ | -3065 | 7391 | 7073 | 8315 | 2519 | 6436 | 11569 | -4842 | -4181 | 6548 | 6368 |
| INMB (VS CAD) ^#^ | -14635 | -4178 | -4496 | -3255 | -9050 | -5134 | 0 | -16412 | -15750 | -5022 | -5201 |
| ICER (VS CD) | 37463^***^ | dominant | 17884^**^ | 14677^*^ | 23373^***^ | 19450^**^ | 12276^*^ | 32408^***^ | 41122 | NA | 19522^**^ |
| ICER (VS ND) | 40726 | NA | 19897^**^ | 16530^**^ | 24499^***^ | 22590^***^ | 13096^*^ | 34094^***^ | 44976 | dominated | 21802^***^ |
| Scenario 4: study time frame changed | | | | | | | | | | | |
| 5 years | | | | | | | | | | | |
| Cost/$ | 61163.04 | 9624.97 | 16683.35 | 15380.24 | 116526.24 | 15592.50 | 18253.20 | 80738.23 | 58478.06 | 9829.96 | 16812.22 |
| Utility/QALY | 1.30 | 0.91 | 1.28 | 1.24 | 1.64 | 1.15 | 1.52 | 1.63 | 1.28 | 0.90 | 1.24 |
| NMB/$^&^ | -36259 | 7772 | 7757 | 8270 | -85185 | 6337 | 10727 | -49561 | -34136 | 7260 | 6885 |
| INMB (VS CAD) ^#^ | -46986 | -2955 | -2970 | -2457 | -95912 | -4390 | NA | -60288 | -44863 | -3467 | -3842 |
| ICER (VS CD) | 128333 | dominant | 18035^**^ | 16324^**^ | 144184 | 23050^***^ | 13586* | 97135 | 128021 | NA | 20536^***^ |
| ICER (VS ND) | 132149 | NA | 19077^**^ | 17440^**^ | 146440 | 24865^***^ | 14145* | 98768 | 132035 | dominated | 21780^***^ |
| 10 years | | | | | | | | | | | |
| Cost/$ | 61761.78 | 9710.91 | 17517.31 | 15726.96 | 117844.00 | 16120.53 | 18897.37 | 80922.79 | 61397.36 | 9827.42 | 17807.15 |
| Utility/QALY | 1.39 | 0.91 | 1.30 | 1.27 | 1.81 | 1.18 | 1.58 | 1.72 | 1.35 | 0.87 | 1.28 |
| NMB/$^&^ | -35243 | 7580 | 7321 | 8500 | -83350 | 6387 | 11290 | -48058 | -35571 | 6701 | 6692 |
| INMB (VS CAD) ^#^ | -46533 | -3710 | -3969 | -2790 | -94640 | -4903 | NA | -59348 | -46861 | -4589 | -4598 |
| ICER (VS CD) | 99874 | dominant | 17883^**^ | 14749^*^ | 114911 | 20300^***^ | 12775* | 83642 | 107437 | NA | 19463^**^ |
| ICER (VS ND) | 108439 | NA | 20016^**^ | 16711^**^ | 120148 | 23739^***^ | 13711* | 87916 | 117469 | dominated | 21882^***^ |
| 20 years | | | | | | | | | | | |
| Cost/$ | 61497.63 | 9899.64 | 17764.81 | 15855.26 | 117368.77 | 16071.79 | 19026.16 | 80927.12 | 62227.29 | 9981.14 | 18130.87 |
| Utility/QALY | 1.39 | 0.91 | 1.30 | 1.27 | 1.81 | 1.18 | 1.60 | 1.72 | 1.35 | 0.87 | 1.28 |
| NMB/$^&^ | -34979 | 7391 | 7073 | 8315 | -82875 | 6436 | 11569 | -48068 | -36401 | 6548 | 6368 |
| INMB (VS CAD) ^#^ | -46548 | -4178 | -4496 | -3254 | -94444 | -5133 | NA | -59637 | -47970 | -5021 | -5201 |
| ICER (VS CD) | 99070 | dominant | 18102^**^ | 14685^*^ | 114242 | 19647^**^ | 12390* | 83466 | 108846 | NA | 19877^**^ |
| ICER (VS ND) | 107496 | NA | 20167^**^ | 16543^**^ | 119410 | 22860^***^ | 13227* | 87688 | 118926 | dominated | 22247^***^ |

* About 1 time the 2021 Gross Domestic Product (GDP) per capita ($12,728);

** 1~1.5 times the 2021 GDP per capita ($12,728-19,092);

*** 1.5~3 times the 2021 Gross Domestic Product (GDP) per capita ($19,092-38,184);

# less than 3 times the 2021 GDP per capita ($38,184);

& Cost-effective threshold = 1.5 times the 2021 GDP ($19,092);

# CA+C had the largest NMB;

NN, first-line nedaplatin-based chemotherapy followed by second-line nivolumab; NT, first-line nedaplatin-based chemotherapy followed by second-line tislelizumab; ND, first-line nedaplatin-based chemotherapy followed by second-line docetaxel; CN, first-line standard chemotherapy followed by second-line nivolumab; CT, first-line standard chemotherapy followed by second-line tislelizumab; CD, first-line standard chemotherapy followed by second-line docetaxel; TID, first-line tislelizumab combined with chemotherapy followed by second-line docetaxel; CAD, first-line camrelizumab combined with chemotherapy followed by second-line docetaxel; PED, first-line pembrolizumab combined with chemotherapy followed by second-line docetaxel; SID, first-line sintilimab combined with chemotherapy followed by second-line docetaxel; SUD, first-line sugemalimab combined with chemotherapy followed by second-line docetaxel; NMB, net monetary benefit; INMB, incremental net monetary benefit; QALY, quality-adjusted life year; ICER, incremental cost-effectiveness ratio.

### eFigure 1 Net Monetary Benefits of All Treatments over Time

NN, first-line nedaplatin-based chemotherapy followed by second-line nivolumab; NT, first-line nedaplatin-based chemotherapy followed by second-line tislelizumab; ND, first-line nedaplatin-based chemotherapy followed by second-line docetaxel; CN, first-line standard chemotherapy followed by second-line nivolumab; CT, first-line standard chemotherapy followed by second-line tislelizumab; CD, first-line standard chemotherapy followed by second-line docetaxel; TID, first-line tislelizumab combined with chemotherapy followed by second-line docetaxel; CAD, first-line camrelizumab combined with chemotherapy followed by second-line docetaxel; PED, first-line pembrolizumab combined with chemotherapy followed by second-line docetaxel; SID, first-line sintilimab combined with chemotherapy followed by second-line docetaxel; SUD, first-line sugemalimab combined with chemotherapy followed by second-line docetaxel.

### eFigure 2 Net monetary benefits of All Treatments When Costs of Third-line treatment Changed

NN, first-line nedaplatin-based chemotherapy followed by second-line nivolumab; NT, first-line nedaplatin-based chemotherapy followed by second-line tislelizumab; ND, first-line nedaplatin-based chemotherapy followed by second-line docetaxel; CN, first-line standard chemotherapy followed by second-line nivolumab; CT, first-line standard chemotherapy followed by second-line tislelizumab; CD, first-line standard chemotherapy followed by second-line docetaxel; TID, first-line tislelizumab combined with chemotherapy followed by second-line docetaxel; CAD, first-line camrelizumab combined with chemotherapy followed by second-line docetaxel; PED, first-line pembrolizumab combined with chemotherapy followed by second-line docetaxel; SID, first-line sintilimab combined with chemotherapy followed by second-line docetaxel; SUD, first-line sugemalimab combined with chemotherapy followed by second-line docetaxel.
